# Structure–Activity Relationships, Molecular Mechanisms, and Ecotoxicological Evaluation Underlying Nucleoside-Mediated Antifouling Activity

**DOI:** 10.3390/biom16040584

**Published:** 2026-04-14

**Authors:** Sandra Pereira, Isabel B. Oliveira, Andreia Palmeira, Maria V. Turkina, Vitor Vasconcelos, Alexandre Campos, Joana R. Almeida

**Affiliations:** 1 CIIMAR/CIMAR LA, Interdisciplinary Centre of Marine and Environmental Research, University of Porto, Terminal de Cruzeiros do Porto de Leixões, 4450-208 Matosinhos, Portugal; sandra.pereira@ciimar.up.pt (S.P.); isabel.oliveira@ciimar.up.pt (I.B.O.); vmvascon@fc.up.pt (V.V.); acampos@ciimar.up.pt (A.C.); 2 Biology Department, Faculty of Sciences, University of Porto, Rua do Campo Alegre, 4169-007 Porto, Portugal; 3 Laboratory of Organic and Pharmaceutical Chemistry, Department of Chemical Sciences, Faculty of Pharmacy, University of Porto, Rua Viterbo Ferreira, 228, 4050-313 Porto, Portugal; apalmeira@ff.up.pt; 4 Department of Biomedical and Clinical Sciences, Faculty of Medicine and Clinical Sciences, Linköping University, 581 83 Linköping, Sweden; maria.turkina@liu.se

**Keywords:** antifouling alternatives, bio-based solutions, nucleosides, hypoxanthine arabinoside, 2′-deoxyinosine

## Abstract

Marine biofouling remains a major challenge for maritime industries, affecting submerged structures and vessels worldwide. The long-standing reliance on biocidal coatings, together with their documented environmental impacts, has led to increasingly restrictive regulations and an urgent demand for environmentally compatible antifouling (AF) solutions. This study evaluates the AF potential and toxicological profile of two nucleoside analogues, hypoxanthine arabinoside (**1′**) and 2′-deoxyinosine (**2′**), selected based on the previously reported non-lethal AF activity of the naturally occurring nucleosides adenosine and 2′-deoxyadenosine from cyanobacteria. Both analogues inhibited the growth of *Navicula* sp. by approximately 60% without inducing mortality and significantly reduced settlement of *Mytilus galloprovincialis* plantigrades, with EC_50_ values of 5.50 µM (**1′**) and 8.54 µM (**2′**), and no lethality detected (LC_50_ > 200 µM). At near-EC_50_ concentrations, both compounds increased acetylcholinesterase and tyrosinase activities, supported by molecular docking results, suggesting involvement of neurotransmission- and byssal formation-related pathways. Proteomic analysis revealed compound-specific molecular responses. No lethal effects were observed in non-target organisms (LC_50_ > 32 µM for *A. amphitrite* and LC_50_ > 50 µM for *A. salina*), and environmental fate modelling predicted low bioaccumulation and rapid degradation. Overall, substitution of the amino group by a carbonyl group preserved AF efficacy without increasing toxicity, highlighting nucleosides as promising low-toxicity AF agents.

## 1. Introduction

**Figure 1:**
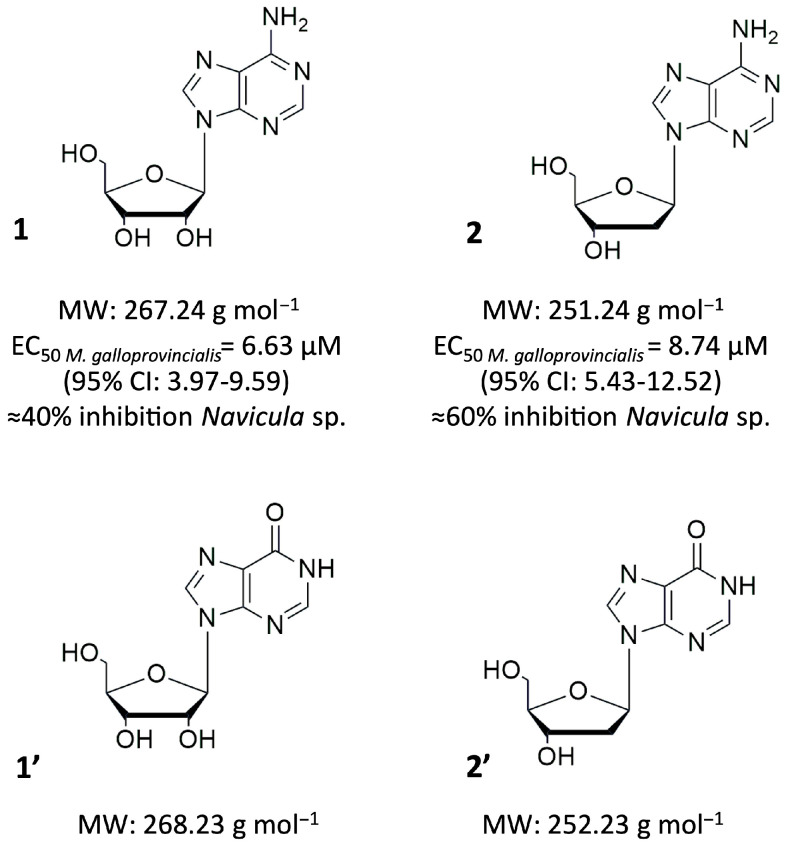
Chemical structure of the nucleosides. **1**: Adenosine; **1′**: Hypoxanthine arabinoside; **2**: 2′-Deoxyadenoside; **2′**: 2′-Deoxyinosine. MW: molecular weight. EC_50_ values obtained for *M. galloprovincialis* plantigrades. Growth inhibition against the marine biofilm-forming diatom *Navicula* sp. [9].

Marine biofouling—the accumulation of diverse micro- and macrofouling organisms such as bacteria, fungi, diatoms, barnacles, mussels, tubeworms, and algae on submerged surfaces [1]—is a naturally occurring process that has evolved into a significant global issue, particularly affecting maritime industries. It contributes to increased drag on vessels, elevated fuel consumption, and the degradation of marine infrastructure. Although biocidal coatings remain the predominant strategy for biofouling control [2], their effectiveness is coupled with environmental drawbacks due to the release of toxic substances into marine ecosystems, raising concerns for both environmental and human health [3,4]. In response to the European Union’s shift towards a sustainable blue economy [5], and the implementation of the Biocidal Products Regulation (BPR, EU 528/2012), there is growing pressure to replace persistent toxic biocides with environmentally safer alternatives. Significant recent efforts have been directed toward the discovery of novel antifouling (AF) compounds, with several comprehensive reviews highlighting advances in marine natural product-based AFagents [6,7,8]. However, new AF agents must meet stricter criteria, demonstrating target-specific efficacy, a well-defined mode of action, minimal toxicity to non-target species, and low potential for bioaccumulation and environmental persistence. Within this context, our investigation of the biotechnological potential of the cyanobacterium *Leptothoe* sp. LEGE 181152 led to the identification of two naturally occurring nucleosides, adenosine (**1**) and 2′-deoxyadenosine (**2**), as novel AF agents. These compounds inhibited the growth of *Navicula* sp. by approximately 40–60% and prevented the settlement of *Mytilus galloprovincialis* plantigrades (EC_50_ = 6.63 − 8.74 µM) while displaying no lethality toward fouling organisms (LC_50_ > 200 µM) or non-target species such as *Artemia salina* and *Amphibalanus amphitrite* nauplii. Mechanistic insights further suggested a modulation of acetylcholinesterase (AChE) activity by both compounds, characterized by a hormetic response. In addition, proteomic analysis of mussel plantigrades exposed to compound 2 revealed altered expression of von Willebrand factor (vWF) and epidermal growth factor (EGF)-like proteins, alongside effects on proteins associated with ciliary function and ATP metabolism [9]. Despite these promising results, nucleosides and their derivatives remain largely unexplored as AF agents. To date, only the nucleoside derivatives kipukasins—isolated from *Aspergillus versicolor* and *Ganoderma australe—*have been evaluated for AF activity against *Amphibalanus amphitrite* cyprids [10] and *Bugula neritina* larvae [11]. Among nature-inspired compounds, AF activity has been reported only for benzimidazole C-nucleosides, where selected acyclic gulo- and gluco-analogues reduced bacterial biofilm formation when incorporated into marine coatings [12]. Consequently, the structural elements regulating AF activity in nucleosides remain essentially unknown. Given that AF activity is a recently described property of nucleosides **1** and **2**, a critical next step is to identify the structural features responsible for their efficacy and selectivity. As reviewed in Seley-Radtke and Yates [13], simple modifications on nucleoside scaffold purine base can influence hydrogen bonding interactions, electronic distribution, and enzyme recognition, and therefore significantly impact biological activity. To test the contribution of amino functional group (-NH_2_) at the 6-position of the purine base, two closely related nucleoside analogues—hypoxanthine arabinoside (**1′**) and 2′-deoxyinosine (**2′**)—were selected. These analogues preserve the ribose or deoxyribose moiety and overall molecular scaffold of **1** and **2**, differing only by the substitution of the amino group with a carbonyl group (C=O) (Figure 1). This conservative modification allows for the evaluation of structure–AF activity relationships while minimizing confounding effects arising from major structural changes. Hypoxanthine arabinoside (**1′**) was originally developed as an antiviral agent [14] and is commonly encountered as a degradation product of vidarabine, whereas 2′-deoxyinosine (**2′**) is a naturally occurring product of 2′-deoxyadenosine (**2**) deamination, catalysed by adenosine deaminase during nucleotide metabolism [15]. Notably, 2′-deoxyinosine (**2′**) has also been associated with biotechnological potential, particularly in the context of anti-cancer and anti-inflammatory activities [16,17].

In this study, we investigated the structure–AF activity relationship of **1′** and **2′** by systematically evaluating their AF efficacy, molecular mechanisms, and predicted environmental impact. AF performance was assessed against representative micro- and macrofouling organisms. Molecular mechanisms were explored through *in vitro* enzymatic assays focusing on tyrosinase (TYR) and AChE, complemented by untargeted whole-organism shotgun proteomics and AChE-target docking simulations. Finally, the environmental safety profile of the compounds was evaluated using toxicity assays on non-target species and in silico predictions of bioaccumulation and degradation potential.

## 2. Materials and Methods

### 2.1. General

The compound hypoxanthine arabinoside (**1′**) (purity ≥98%, CAS: 7013-16-3) was procured from Biosynth Ltd. (Compton, UK), and 2′-deoxyinosine (**2′**) (purity ≥98%, CAS: 890-38-0) was acquired from Merck (Darmstadt, Germany).

### 2.2. Antifouling Activity

#### 2.2.1. Settlement Inhibition of *M. galloprovincialis* Plantigrades

The settlement inhibition assay followed the methodology previously reported by Almeida *et al*. [18]. Briefly, samples were obtained from intertidal rock pools at Memória Beach, Matosinhos, Portugal (41°13′59″ N; 8°43′28″ W), during periods of low neap tide. Active plantigrades of *M. galloprovincialis* were selected and distributed into 24-well microplates, using four replicates per conditions (five individuals per well). Organisms were exposed to the test solutions for 15 h at 18 ± 1 °C under dark conditions. Test compounds were evaluated at 25 and 50 µM. Each assay included a negative control (0.1% DMSO) and a positive control (5 μM CuSO_4_), with all solutions prepared in filtered seawater. Anti-settlement effects were determined by assessing the formation of byssal threads and their attachment to the substrate. Additionally, the half-maximal effective concentration (EC_50_), corresponding to 50% inhibition of larval settlement, and the median lethal dose (LC_50_) were calculated.

##### *M. galloprovincialis* Settlement Recovery

The reversibility of the AF effects on mussel plantigrades was assessed following short-term exposure (15 h), according to previously described procedures [19]. Initially, a settlement inhibition assay was conducted as outlined above, in which plantigrades were exposed to the compounds for 15 h, at 3.1 and 12.5 µM for **1′** and at 6.2 and 25 µM for **2′**. These concentration ranges were selected to include the respective EC_50_ values. Assays included both negative (0.1% DMSO) and positive (5 μM CuSO_4_) controls. The inhibitory effect on settlement was confirmed immediately after the exposure period. Subsequently, each individual was carefully transferred to a clean microplate containing only filtered natural seawater, with appropriate controls maintained. After an additional 15 h recovery period, settlement was re-evaluated under all conditions to determine the reversibility of the observed effects.

#### 2.2.2. Growth Inhibition of Marine Bacteria and Diatom

To further investigate the activity of the compounds against microfouling organisms, a panel of five marine biofilm-forming bacterial strains—*C. marina* CECT 4278, *V. harveyi* CECT 525, *H. aquamarina* CECT 5000, *P. atlantica* CECT 570, and *R. litoralis* CECT 5395—was acquired from the Spanish Type Culture Collection (CECT). Antibacterial activity was evaluated following previously reported procedures [18]. Briefly, bacterial strains were cultured in Marine Broth (Condalab, Madrid, Spain) and distributed into 96-well flat-bottom microplates, ensuring an initial optical density of 0.1 at 600 nm (OD600). Cells were exposed to compounds at 25 and 50 μM and incubated at 26 °C for 24 h, except for *R. litoralis*, which required 72 h. Each condition was tested in quadruplicate. A solution of Marine broth containing 1% DMSO served as the negative control, while a 1:100 penicillin-streptomycin-neomycin stabilized solution (Sigma P4083, Merck, Darmstadt, Germany) was used as the positive control. Bacterial growth inhibition was determined by measuring absorbance at 600 nm using a microplate reader (Biotek Synergy HT, Winooski, VT, USA).

For the evaluation of anti-microalgal activity, a benthic marine diatom (*Navicula* sp.) was obtained from the Spanish Collection of Algae and tested according to Antunes *et al*. [19]. Diatom cultures were prepared in Guillard’s (F/2) medium supplemented with silica (Sigma G9903), at an initial concentration of 2–4 × 10^4^ cells mL^−1^. The assays were conducted in 96-well flat-bottom microplates, where cells were exposed to the compounds at 25 and 50 µM over a 10-day period at 20 °C. All treatments were performed in quadruplicate. Guillard’s (F/2) medium containing 1% DMSO was used as negative control, while cycloheximide (3.55 μM) served as the positive control. Inhibition of diatom growth was assessed by comparing cell densities across different conditions.

### 2.3. Molecular Targets on M. galloprovincialis

#### 2.3.1. *In Vitro* TYR Activity

TYR inhibitory activity was evaluated using *Agaricus bisporus* tyrosinase (EC 1.14.18.1), following the method described by Adhikari *et al*. [20] with minor modifications [18]. For each assay condition, 50 µL of TYR (25 U mL^−1^) prepared in phosphate buffer (50 mM, pH 6.5) was added. Test compounds were assessed at 25 and 50 µM. The reaction was initiated by the addition of L-DOPA (25 mM), prepared in the same phosphate buffer (50 mM, pH 6.5). DMSO (0.2%) was used as negative control, while kojic acid (100 µM) was used as positive control. All experiments were performed in quadruplicate. Enzymatic activity was monitored by measuring absorbance at 475 nm over 5 min at 25 °C.

#### 2.3.2. *In Vitro* AChE Activity

AChE inhibitory activity of the compounds was evaluated using AChE Type V-S from *Electrophorus electric* (SIGMA C2888, E.C. 3.1.1.7), following the method originally described by Ellman *et al*. [21] with modifications reported by Almeida *et al*. [22]. Succinctly, the reaction solution consisted of 30 mL of phosphate buffer (0.1 M, pH = 7.2), supplemented with 1 mL of 5,5’-Dithiobis(2-nitrobenzoic acid) (DTNB, 10 nM), prepared in phosphate buffer (0.1 M, pH = 7.2) containing sodium hydrogen carbonate, and 200 µL of acetylthiocholine iodide (0.075 M). For each condition, 50 µL of pure AChE solution (0.25 U mL^−1^) was combined with 250 µL of the reaction solution. The tested compounds were evaluated at 25 µM and 50 µM. DMSO (0.2%) and eserine (200 µM) were used as negative and positive controls, respectively, while a blank was prepared using 50 µL of phosphate buffer instead of the enzyme. All conditions were conducted in quadruplicates. Enzymatic activity was monitored by measuring absorbance at 412 nm over 5 min at 25 °C.

##### Docking Study of AChE

The crystal structure of AChE from *Electrophorus electric* (PDB code: 1C2O) [23] was retrieved from the protein databank (PDB) and used as the receptor for the in silico molecular docking analyses. The chemical structures of the test compounds, along with two reference AF agents exhibiting AChE inhibitory activity—pulmonarins A and B [24]—were drawn and minimized. Geometry optimization was carried out using the semi-empirical Austin Model 1 (AM1) method, applying the HyperChem 7.5 software (Hypercube, FL, USA) [25], until the energy gradient between consecutive steps fell below 10^−1^ kcal mol^−1^ Å^−2^. Docking simulations were performed with Autodock Vina v1.2.x (Molecular Graphics Lab, The Scripps Research Institute, San Diego, CA, USA) [26], using an exhaustiveness parameter of 8. The search space was defined by a grid box of 15.0 × 20.0 × 15.0 Å, encircling the acetylcholine active site. For each ligand, nine binding conformations were generated. The poses exhibiting the lowest docking scores were selected for further evaluation of interaction patterns and visual inspection using PyMol 1.3 (Schrödinger, New York, NY, USA) [27].

#### 2.3.3. Untargeted Proteomic Analysis and Functional Enrichment of Mussel Plantigrades

*M. galloprovincialis* plantigrades were exposed to the tested compounds at 12.5 µM, a concentration close to the EC_50_ previously determined in plantigrade settlement assays. Following exposure, plantigrades from both negative control (0.1% DMSO) and treated groups were collected for proteomic analysis, using four replicates per condition (ten plantigrades per replicate), in accordance with the approach described by Pereira *et al*. [9]. Protein extraction and peptide preparation for liquid chromatography-mass spectrometry (LC-MS) were carried out as previously reported by Campos *et al*. [28]. Succinctly, defrosted samples were solubilized in lysis buffer (composed of 2% (*w*/*v*) SDS, 100 mM Tris-HCl at pH 7.6, 0.1 M DTT, and 1:50 protease inhibitor) and filter-aided sample preparation (FASP) was used for protein digestion [29]. After reconstitution in formic acid (0.1%, *v*/*v*), peptide separation was performed on an EASY nLC 1200 chromatographic system (Thermo Scientific, Waltham, MA, USA) equipped with C18 Acclaim PepMap 100, 75 μm × 2 cm pre-column (Thermo Scientific) and EASY-Spray C18 analytical column (PepMap RSLC C18, 2 μm, 100A 75 μm × 25 cm, Thermo Scientific). Elution used was achieved at a flow rate of 0.3 μL min^−1^ using a gradient of solvent A (0.1% formic acid in water) and solvent B (0.1% formic acid in 80% acetonitrile), progressing from 6% to 30% B over 65 min, followed by an increase to 100% B in 20 min and a final hold at 100% B for 5 min. The eluting peptides were analyzed online by a QExactive HF Hybrid Quadrupole-Orbitrap Mass Spectrometer (Thermo Scientific), operating in a positive ion mode under data-dependent acquisition mode. Full MS scans were acquired at a resolution of 120,000 across an *m*/*z* range of 380–1400. The 15 most intense multiple charged ions (isolation window of 1.2 *m*/*z*) were selected for fragmentation at a resolution of 30,000, with a dynamic exclusion of 30 s. Protein identification was performed using Sequest HT algorithm implemented in Proteome Discoverer (v. 2.5.0.400, Thermo Scientific), searching against the Mollusca UniProt Knowledgebase [30] (Taxon ID: 6447, March 2023, 753,986 entries). Search parameters included trypsin specificity, a parent ion tolerance of 10 ppm, and a fragment ion mass tolerance of 0.080 Da. Carbamidomethylation of cysteine residues was set as a fixed modification, while methionine oxidation was allowed as a variable modification. Peptide and protein identifications were further validated using Scaffold (v5.3.2). Acceptance criteria required peptide identifications with a probability greater than 95% (based on Percolator posterior error probability) [31], and protein identifications exceeding 99.0% probability with at least two identified peptides, as determined by the Protein Prophet algorithm [32]. Proteins sharing significant peptide evidence were grouped according to parsimony principles.

#### 2.3.4. Bioinformatic Analysis

The set of identified proteins was examined for entries previously described in the literature as being associated with mussel adhesion mechanisms. In parallel, broader functional insights were obtained through sequence homology analysis using BLASTp against *Magallana gigas* protein databases (UniProt KB 2025_01, Ensembl Metazoa 2024_08), implemented in Blast2GO (v6.0.3) [33,34,35], applying an e-value cutoff of 1 × 10^−3^. Given the more extensive annotation available for *M. gigas* compared to *M. galloprovincialis*, functional inference was based on homologous sequences identified in this species. Subsequently, functional enrichment and classification analyses were conducted using g:Profiler (e112_eg59_p19_25aa4782, updated 3 February 2025) [36]. The resulting functional interaction networks were then constructed and visualized with Cytoscape (v3.10.3) [37].

### 2.4. Environmental Behaviour

#### 2.4.1. *Artemia Salina* Bioassay

General marine ecotoxicity of the tested compounds was evaluated using the brine shrimp (*A. salina*) nauplii lethality assay, as previously described [18]. Cysts were hatched in nutrient-enriched seawater for approximately 24 h at 26 °C to obtain active nauplii. The compounds were tested at 25 and 50 μM. Assays were carried out in 96-well microplates, with 15–20 nauplii per well and eight replicates per condition. Exposure was conducted in the dark at 25 °C over a period of 48 h. DMSO (1%) was used as negative control, while potassium dichromate (13.6 μM) was used as positive control.

#### 2.4.2. *Amphibalanus amphitrite* Bioassay

The toxicity of the compounds was further evaluated using *A. amphitrite* nauplii, following the procedure described by Pereira *et al*. [38]. Newly released and actively swimming nauplii (<3 h post-release) were exposed to the test solutions at concentrations ranging from 0.125–8 µg mL^−1^ (0.5–32 µM). Exposures were conducted in glass tubes containing 3 mL of solution, with approximately 30–60 larvae per tube, in triplicate. The assay was carried out for 24 h at 28 °C under dark conditions, without feeding. Mortality was determined based on the absence of movement in the nauplii. A negative control consisting of DMSO (0.1%) was included. The assay was performed twice, and the results were combined for subsequent analysis.

#### 2.4.3. *In Silico* Environmental Fate Predictions

A set of physicochemical properties and environmental fate of the compounds was predicted using computational models available within the Estimation Programs Interface (EPI) Suite™, developed by the US Environmental Protection Agency’s Office of Pollution Prevention and Toxics and Syracuse Research Corporation (SRC) [39]. The analyses included bioconcentration potential (BCFBAF™ v3.01), partitioning behaviour (KOWWIN™ v1.68), biodegradability (BIOWIN™ v4.10), and soil adsorption capacity (KOCWIN™ v2.00).

### 2.5. Data Analysis

Prior to statistical analysis, all datasets were evaluated for normality using the Shapiro–Wilk test and for homogeneity of variances with Levene’s test. When required, data transformations were applied to meet test assumptions. The screening results from the different bioassays were analysed using one-way analysis of variance (ANOVA) followed by Dunnett’s test (*p* < 0.01). In cases where assumptions were not met, Welch’s ANOVA was applied, followed by Dunnett’s T3 test (*p* < 0.01). For the diatom growth inhibition assay, differences between concentrations were assessed using Tukey HSD test (*p* < 0.01). EC_50_ for *M. galloprovincialis* plantigrade settlement inhibition was estimated through Probit regression analysis. Model fit was evaluated using Pearson’s Goodness-of-Fit Chi-Squared test (*p* < 0.01), and 95% lower and upper confidence limits (LCL and UCL) were calculated. All ANOVA and Probit analyses were conducted using IBM SPSS Statistics (version 30). For mussel plantigrades, the compounds’ efficacy versus toxicity was expressed as the therapeutic ratio (LC_50_/EC_50_) [18,40]. Proteomic data analysis was performed in R: programming language (version 4.4.1) [41] using the “DEP” package [42]. Proteins with missing values were initially filtered, retaining only those consistently identified in all replicates of at least one experimental condition. Remaining missing values were subsequently imputed. Differently expressed proteins (DEPs) between treatment and control groups were defined based solely on a log_2_ fold change (log_2_FC = log_2_(1.5)).

## 3. Results and Discussion

### 3.1. AF Activity and Toxicity Against Micro and Macrofouling Organisms

**Figure 2:**
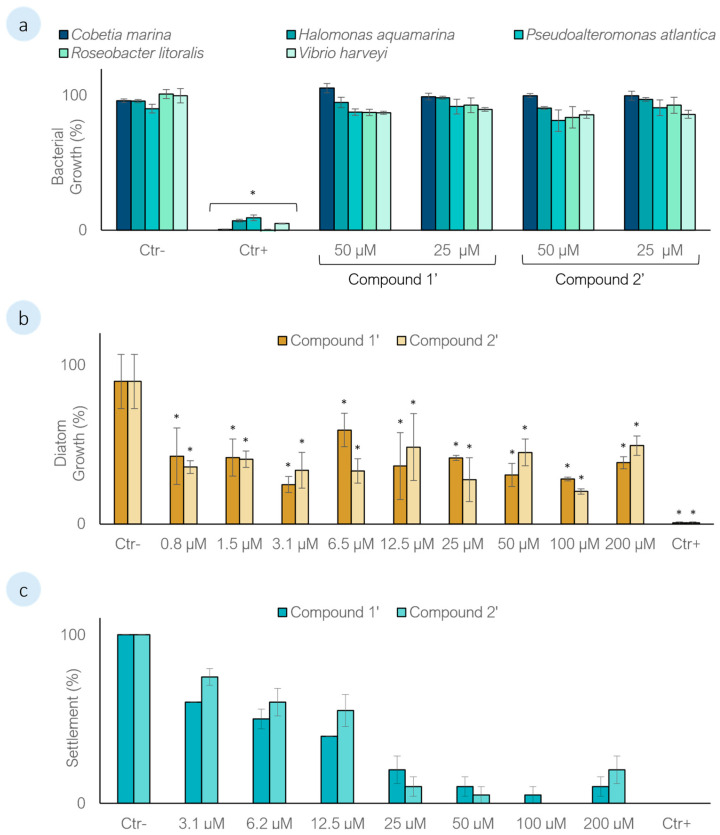
Antifouling activity of **1′** and **2′**. (**a**) Growth inhibition of five marine biofilm-forming bacteria screening bioassay. Compounds were screened at 25 and 50 µM. Ctr−: DMSO (1%); Ctr+: 1:100 penicillin-streptomycin-neomycin stabilized solution; (**b**) Dose–response against the marine biofilm-forming diatom *Navicula* sp. Ctr−: DMSO (1%); Ctr+: Cycloheximide (3.55 µM); (**c**) Dose–response against *M. galloprovincialis* plantigrades. Ctr−: DMSO (0.1%); Ctr+: CuSO_4_ (5 µM). * Significant differences compared to the negative control (Dunnett’s test, *p* < 0.01).

In this work, the nucleoside analogues hypoxanthine arabinoside (**1′**) and 2’-deoxyinosine (**2′**) were evaluated for their AF potential, following the previously reported AF activity of their structural counterparts **1** and **2**. As this represents the first study exploring the biotechnological AF potential of **1′** and **2′**, their activity was assessed against a panel of microfouling organisms, including five strains of marine biofilm-forming bacteria and a marine biofilm-forming diatom, as well as against a widely distributed macrofouling species. To evaluate microfouling inhibition, marine biofilm-forming bacterial strains and the diatom *Navicula* sp. were exposed to **1′** and **2′**. A growth inhibition threshold of ≥30% was established as the criterion for AF activity. In the initial screening against the five bacterial strains, neither compound induced significant growth inhibition at concentrations of 25 and 50 µM (Dunnett’s test, *p* > 0.01) (Figure 2a). In contrast, both nucleosides displayed significant inhibitory effects against the marine diatom *Navicula* sp., with growth inhibition ≥30% at the same concentrations (Dunnett’s test, *p* < 0.01), prompting further dose–response evaluation. Across the entire concentration range tested (0.8–200 µM), both **1′** and **2′** consistently inhibited diatom growth by approximately 60% compared to the control (Dunnett’s test, *p* < 0.01) (Figure 2b). This response contrasts with the differential inhibitory effect previously observed for **1** (≈40%) and **2** (≈60%), which were proposed to be associated with an increased lipophilicity resulting from the absence of hydroxyl group (-OH) at the 2′ position of the ribose moiety [9,43]. Since **2′** also lack this hydroxyl group, a plausible explanation for their comparable inhibitory profile is that the increased lipophilicity conferred by the carbonyl group (=O) at the position 6 of the purine system, substituting the amino group (-NH_2_), may outweigh the structural differences between **1′** and **2′**, resulting in a similar inhibitory response [43]. Nonetheless, no clear dose–response relationship could be established for either compound, suggesting that a threshold-like mechanism may underlie their AF activity against *Navicula* sp., consistent with observations reported for **1** and **2** [9].

The AF activity of the compounds was further evaluated against the macrofouling species *M. galloprovincialis*, one of the most widespread macrofoulers globally and a well-established model organism for settlement inhibition bioassays [44,45,46]. Mussel plantigrades were exposed to the compounds for 15 h, and, after the initial screening at concentrations of 25 and 50 µM, both compounds showed significant settlement inhibition (Dunnett’s test, *p* < 0.01). A dose–response bioassay was performed (Figure 2c), and the half-maximal effective concentration that inhibited 50% of mussel settlement was assessed for **1′** (EC_50_ = 5.50 µM) and **2′** (EC_50_ = 8.54 µM) (Table 1). The results showed EC_50_ values for **1′** and **2′** below the U.S. Navy’s program reference threshold (EC_50_ < 25 µg mL^−1^). The values were comparable to those reported for **1** and **2** (EC_50_ = 6.63 and 8.74 µM, respectively) suggesting that the AF activity may not be linked to the amino group. Other nucleosides, such as kipukasins (D, E, H, K and L) and their diacetylated derivatives (diacetylkipukasin D and E), have previously been evaluated for AF activity using different model organisms. Kipukasins D, E and H as well as diacetylkipukasin D and E exhibited low inhibitory effects on the settlement of *A. amphitrite* cyprids, with reported EC_50_ values ranging from 44.50 to 61.30 µM [10]. In contrast, kipukasins K and L were shown to reduce larval settlement of the bryozoan *Bugula neritina* at a concentration of 25.00 µg mL^−1^ [11]. In comparison, the inhibitory activity of compounds **1′** and **2′** against mussel plantigrades was considerably stronger. Additionally, the efficacy of compound **1′** can be comparable to the commercial AF agent ECONEA^®^ (EC_50_ = 1.40 µ mL^−1^) efficacy in preventing the settlement of mussel plantigrades [18].

Compounds exhibiting AF activity with EC_50_ values around ~2 µg mL^−1^, such as the nucleoside derivatives described in this study, fall within the range commonly reported for many effective marine AF natural products. Indeed, a wide variety of bioactive compounds—including terpenoids, alkaloids, and polyketides—typically display EC_50_ values in the range of 1–10 µg mL^−1^ against relevant fouling organisms [7,47,48]. Although some recently described metabolites, such as madeirone and related compounds [49], exhibit higher potency at sub-microgram levels, AF performance cannot be evaluated solely based on EC_50_ values. Other factors, including environmental compatibility, mode of action, chemical stability, and synthetic accessibility, are critical for practical applications. Therefore, despite not always reaching the lowest EC_50_ values reported for certain marine natural products, nucleosides constitute a valuable and competitive addition to the current AF chemical toolbox.

Overall, the AF profile **1′** and **2′** showed activity against *Navicula* sp. and *M. galloprovincialis* plantigrades, but no inhibitory effect on the growth of marine biofilm-forming bacteria, indicating a degree of biological specificity, with putative molecular mechanisms preferentially targeting eukaryotic processes rather than prokaryotes. This aligns with that observed for **1** and **2** and supports a more selective AF mode of action. Such selectivity is typically associated with safer and more environmentally compatible AF agents, particularly when compared to broad-spectrum biocides known for their high marine toxicity [8]. Future works should focus on testing other eukaryotic biofouling organisms to confirm this apparent selectivity.

### 3.2. Putative Molecular Targets of the AF Activity on M. galloprovincialis Plantigrades

Understanding the mechanisms underlying the AF action of compounds is becoming increasingly important for the development of new AF agents, particularly considering the regulatory demands set by the Biocidal Products Regulation (BPR, EU 528/2012). However, pinpointing a single molecular target or pathway remains a considerable challenge due to the complexity of the biological processes involved in biofouling [8]. In this study, the potential protein targets underlying the AF effects of compounds **1′** and **2′** were investigated in *M. galloprovincialis* plantigrades, with a focus on exploring multiple pathways potentially involved in settlement inhibition.

#### 3.2.1. AChE and TYR Pathways

**Figure 3:**
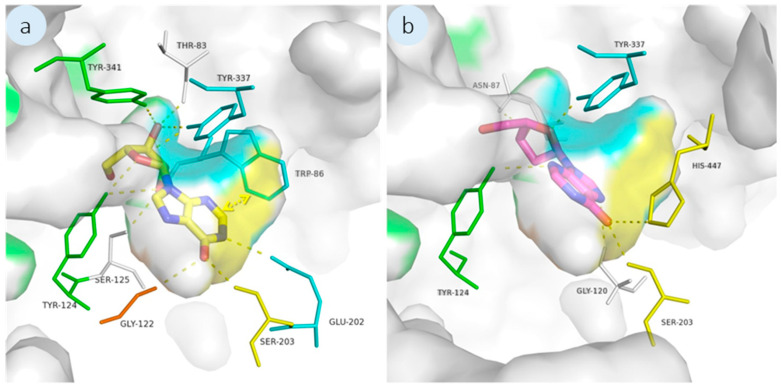
AChE docking study for **1′** (**a**) and **2′** (**b**). Detailed view of **1′** (yellow sticks) and **2′** (pink sticks) docked in AChE binding site (PDB code: 1C2O). Hydrogen interactions are represented with yellow broken lines. Π-Stacking and Π-hydrogen donor interactions are represented with a yellow and orange double arrow, respectively. Residues involved in those interactions are represented as lines and labelled. AChE is represented as transparent surface, where the catalytic triad, PAS, anionic sites and oxyanion hole are represented in yellow, green, blue and orange, respectively.

Enzymes such as AChE, are affected by AF compound exposure in fouling organisms, as AChE is implicated in cholinergic neural signalling during larval settlement [50]. In here, we explore the effects of the compounds on this pathway through docking studies and *in vitro* enzymatic assays. Recently, ligand- and structure-based computer-aided drug design (CADD) tools have been used to anticipate the AF activities of compounds as well as their molecular mechanism of action [51,52]. Furthermore, docking studies have already been employed in our research to anticipate how two hydroxyxanthones will bind to *Electrophorus electric* AChE [53]. As such, the molecular binding mode of the **1′** and **2′** to AChE was conducted by docking studies using AutoDock Vina. A long, tight, and hydrophobic cavity is visible in the crystallographic structure of the serine hydrolase *Electrophorus electric* AChE [23]. The catalytic triad (Ser-203, Glu-334, and His-447) that makes up the AChE active site is situated near the base of the gorge. Here, acetylcholine is converted into choline and acetate [54]. The oxyanion hole formed by Gly-121, Gly-122, and Ala-204 determines the reactive intermediates and transition states that are generated during the process [55]. The side chains of Trp-86, Glu-202, and Tyr-337 are combined to create the anionic subsite, which is primarily in charge of binding the quaternary trimethylammonium tail group of Ach [56]. Tyr-72, Asp-74, Tyr-124, Glu-285, Trp-286, and Tyr-341 are among the aromatic residues that make up the peripheral anionic site (PAS) near the gorge’s entrance. This PAS forms a hydrophobic region that binds acetylcholine and transfers it to the deep catalytic site [57]. Studies on AChE from a variety of species indicate that some AChE inhibitors can bind at the catalytic site and act as competitive inhibitors [58]. At the active site gorge, AChE inhibitors can also bind to PAS, thus hindering the catalysis by sterically blocking ligands from entering and leaving the active site portal and by allosterically altering the catalytic triad’s conformation [59]. As positive controls in the in silico docking study, pulmonarin A and B, two *Ascidian synoicum* AF compounds, were found to exhibit competitive AChE inhibition without any antibacterial or cytotoxic effect [24,60]. The top-ranked poses of pulmonarin A and B have docking scores of −5.9 and −6.8 kcal mol^−1^, respectively. Compared to positive controls, compounds **1′** and **2′** were predicted to have a higher affinity towards the AChE active site (docking score of −7.3 and −7.1 kcal mol^−1^, respectively) (Figure 3).

As previously described by our group, the interactions between known inhibitors pulmonarin A and B and the enzyme are characterized by several hydrogen bonds with residues from PAS, oxyanion hole, anionic site, and catalytic triad [53]. Both compounds dock inside the groove with the purinone ring fitting deeply into the catalytic groove and the oxolanyl ring blocking the entrance of the gorge (PAS) (Figure 3). Compound **1′** establishes hydrogen as a bond network that encloses it securely in the target cavity through residues from the catalytic triad (Ser-203), PAS (Tyr-124), anionic site (Glu-202 and Tyr-337), oxyanion hole (Gly-122) and other residues such as Thr-83, Ser-125 and Tyr-341 (Figure 3a), while **2′** establishes hydrogen interactions with residues from the catalytic triad (His-447 and Ser-203), PAS (Tyr-124), anionic site (Tyr-337), and with other residues such as Asn-87 and Gly-120 (Figure 3b). Most binding patterns are shared among **1′** and **2′**, differing mainly in the number and distribution of hydrogen bonds, particularly the interaction with the oxyanion hole, which was exclusively observed for **1′**. These differences are likely influenced by subtle structural variations between the two nucleosides, including the presence or absence of hydroxyl groups in their sugar moieties. When compared with previously reported docking studies for **1** and **2**, additional distinctions emerge. Specifically, π-hydrogen donor interactions were observed for **1**, whereas π-stacking interactions characterized the binding of **2**, interactions that were not prominent for **1′** and **2′**. These differences may be associated with the substitution of the amino group in the purine ring by a carbonyl group in the hypoxanthine derivatives, highlighting how small modifications in the nucleobase can influence interaction patterns and, ultimately, structure–activity relationships. Crystallographic structures of AChE have shown that hydrogen bonds are established between the inhibitor and residue Ser-203 [61]. Moreover, computational studies have revealed that the most stable conformations of AChE: inhibitor complexes show Ser-203 and His-447 residues close to the inhibitor [62]. Amino acid residues Tyr-337 and Tyr-341 are involved in the binding of reversible known AChE inhibitors [63,64]. Moreover, Trp-86 has been reported as relevant in stabilizing irreversible inhibitors in the AChE binding pocket [65]. Gly-122 is thought to be involved in the formation of acetylcholine reactive intermediates and transition states that are formed during the reaction [66] and it is predicted as being in contact with most AChE ligands [67]. It has previously been reported that Tyr-124, which integrates PAS, plays a crucial role in the binding of *Electrophorus electricus* AChE inhibitors [59,68,69]. Other residues such as Thr-83, Glu-202 and Ser-125 have also been described as important in the interaction of inhibitors with AChE [70,71,72,73]. Taken together, the docking analyses suggest that **1′** and **2′** establish multiple interactions within the AChE catalytic gorge, with predicted binding affinities surpassing those of known inhibitors, reinforcing the relevance of this target and reflecting the trends previously observed for **1** and **2**.

**Figure 4:**
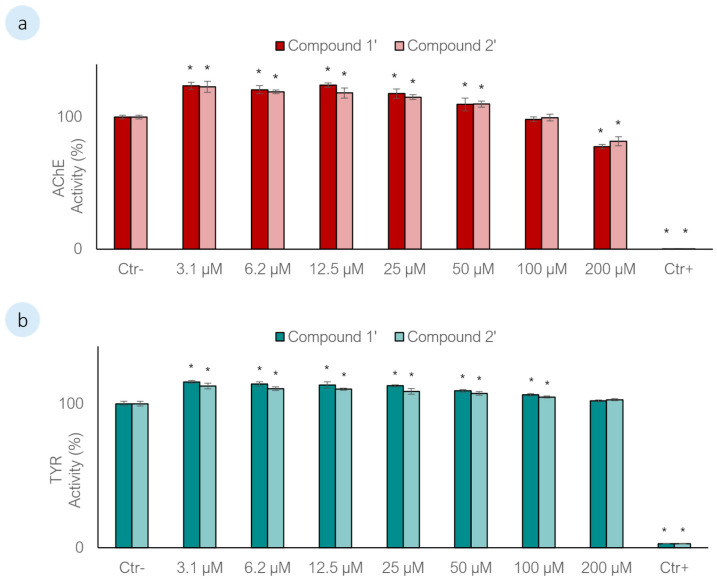
*In vitro* enzymatic activity assays of **1′** and **2′**. (**a**) AChE activity. Ctr−: DMSO (0.2%); Ctr+: Eserine (200 µM). (**b**) TYR activity. Ctr-: DMSO (0.2%); Ctr+: Kojic acid (200 µM). * Significant differences compared to the negative control (Dunnett’s test, *p* < 0.01).

The effects of compounds **1′** and **2′** on AChE were further evaluated through *in vitro* enzymatic assays. An initial screening was performed at concentrations of 25 µM and 50 µM (four replicates per treatment) revealed that both nucleosides significantly increased AChE activity following exposure (Dunnett’s test, *p* < 0.01). To better characterize this response, a wider concentration range (3.1–200 µM) was subsequently tested (Figure 4a), revealing a biphasic effect. At lower concentrations, both nucleosides significantly enhanced AChE activity by approximately 23% (Dunnett’s test, *p* < 0.01), whereas at higher concentrations a significant inhibition of enzymatic activity (≈20%) was observed (Dunnett’s test, *p* < 0.01), consistent with a hormetic response [74]. This concentration-dependent modulation of AChE activity is in line with the high binding affinities predicted by the docking studies. At low concentrations, the stabilization of residues within the catalytic triad and anionic site may promote a more favourable active-site conformation, enhancing catalytic efficiency. Conversely, at higher concentrations, excessive target occupancy or subtle conformational constraints may impair enzyme turnover, resulting in reduced activity. Functionally, enhanced AChE activity at low concentrations may accelerate acetylcholine hydrolysis, reduce neurotransmitter availability and potentially disrupt cholinergic signalling pathways. Given the known role of cholinergic regulation in byssal thread production in mussels, such modulation could impair attachment processes and settlement efficiency, providing a plausible mechanistic link to the observed AF activity at concentrations close to the EC_50_ values. From a structure–activity relationship perspective, the similar enzymatic responses produced by **1′** and **2′** suggest that their effects on AChE activity are not primarily driven by the presence or absence of the amino group in the purine moiety. This interpretation is further supported by comparable biphasic responses previously reported for **1** and **2**, reinforcing the notion that nucleoside-based structures can act as modulators rather than classical inhibitors of AChE. However, there is limited information about the effects of AChE activity modulation in mussels, since most existing studies focus on enzyme inhibition rather than activation [38,75], restricting the interpretation of these early findings. Considering this, future studies should focus on validating the significance of the effect of these nucleosides on the AChE activity through *in vivo* assays on exposed mussel plantigrades.

TYR is another well-known molecular target for investigating the effects of AF agents. This enzyme plays a pivotal role in mussel adhesion by catalysing two key reactions: the oxidation of tyrosine residues to reactive DOPA residues in mussel foot protein-1 within the byssal plaques, and the conversion of DOPA to o-quinone, which promotes the curing and hardening of byssal threads. Both processes are essential for strong attachment [76]. TYR is also involved in various physiological processes, including immune responses, cuticle sclerotization, wound healing, and shell biomineralization [77]. To evaluate the effects of compounds **1′** and **2′** on TYR, *in vitro* assays were conducted at concentrations of 25 µM and 50 µM, with four replicates per treatment. An initial screening revealed a significant increase in TYR activity following exposure to both compounds (Dunnett’s test, *p* < 0.01). A broader concentration range (3.1–200 µM) was then tested (Figure 4b), showing a significant 15% increase in TYR activity at lower concentrations (Dunnett’s test, *p* < 0.01). However, no significant effects were observed at higher concentrations (Dunnett’s test, *p* > 0.01). These findings align with previous observations by Almeida *et al*. [53], in which a nature-inspired xanthone also enhanced TYR activity *in vitro*. The authors suggested that this may be linked to the upregulation of immune responses upon exposure or to increased demand for proteins involved in cytoskeletal dynamics and adhesive structure formation. Supporting this hypothesis, the proteomic analysis in the present study revealed changes in the expression of cytoskeleton-associated and TYR-related proteins, particularly in response to compound **1′**. Interestingly, this outcome contrasts with previous results for compounds **1** and **2**, which did not induce any changes in TYR activity, suggesting that minor structural modifications in nucleosides may differentially influence enzymatic pathways involved in mussel adhesion.

#### 3.2.2. Mussel Plantigrades’ Untargeted Proteome Analysis

**Figure 5:**
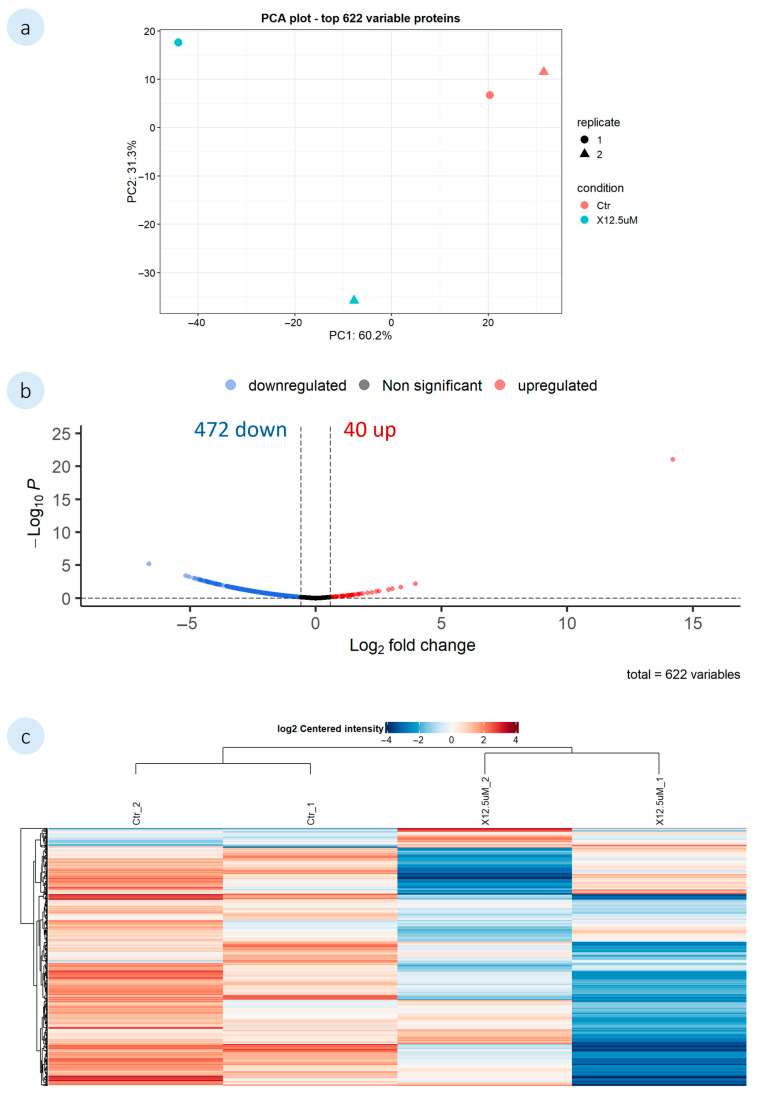
Proteomic analysis of *M. galloprovincialis* plantigrades exposed to **1′**. Analysis was performed by pairwise comparisons between the exposed group (12.5 µM) and the control (0.1% DMSO). (**a**) Principal component analysis (PCA) of the 622 proteins included in the analysis; (**b**) Volcano plot of the DEPs in response to the compounds, when compared to the control (log_2_FC = log_2_(1.5)). Downregulated DEPs are represented by blue dots, and the upregulated by red dots. Non significant proteins are displayed as grey dots; (**c**) The heatmap illustrates relative expression of DEPs across treatments (red: upregulated; blue: downregulated). The rows represent the DEPs. The columns represent the tested groups.

**Figure 6:**
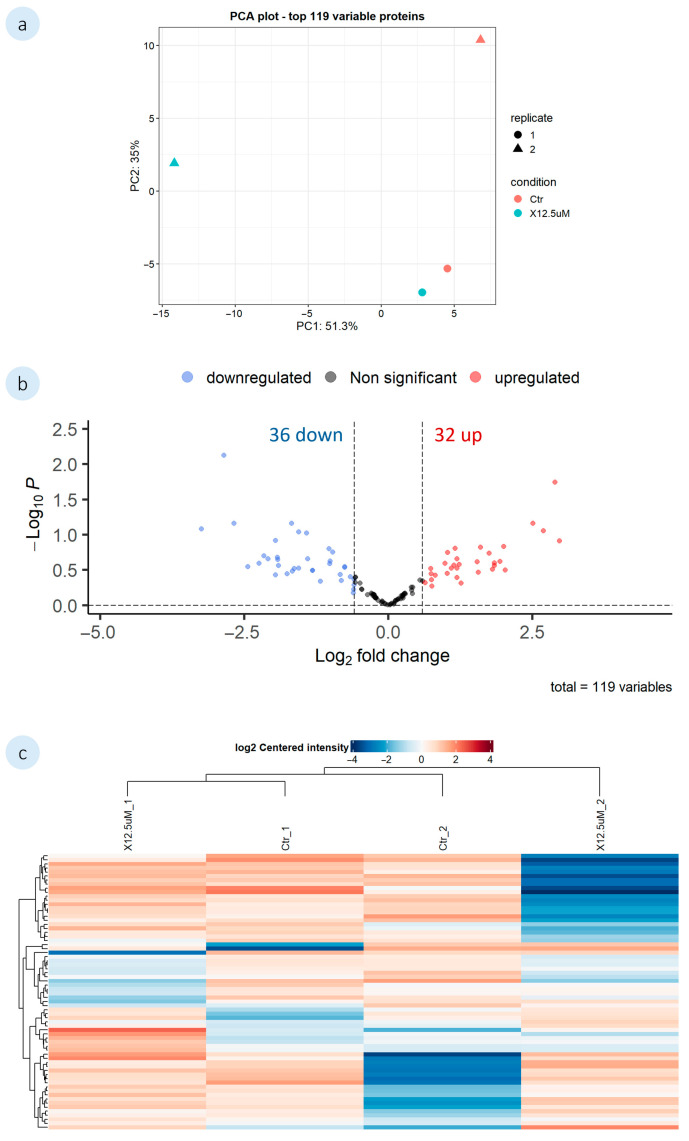
Proteomic analysis of *M. galloprovincialis* plantigrades exposed to **2′**. Analysis was performed by pairwise comparisons between the exposed group (12.5 µM) and the control (0.1% DMSO). (**a**) Principal component analysis (PCA) of the 119 proteins included in the analysis; (**b**) Volcano plot of the DEPs in response to the compounds, when compared to the control (log_2_FC = log_2_(1.5)). Downregulated DEPs are represented by blue dots, and the upregulated by red dots. Non significant proteins are displayed as grey dots; (**c**) The heatmap illustrates relative expression of DEPs across treatments (red: upregulated; blue: downregulated). The rows represent the DEPs. The columns represent the tested groups.

The action of **1′** and **2′** on mussel plantigrades was investigated using shotgun proteomics to ascertain the effects on the AF mechanisms. The mussel plantigrades were exposed to 12.5 µM of each compound, and a control group (0.1% DMSO) also included in the analysis. During the DEP analysis, proteomic data missing values were filtered and only proteins identified in all replicates of at least one condition were considered. A list of all the DEPs identified for both compounds is described in the Supplementary Tables S1 and S2. The proteomic data analysis of *M. galloprovincialis* plantigrades exposed to both compounds were represented in the principal component analysis (PCA), Venn diagrams, volcano plots and heatmaps (Figure 5 and Figure 6). The PCA shows the variations in the proteins between the control and the exposed groups, as well as the variations among replicates. Furthermore, volcano plots highlight the differential expression of proteins in the mussel plantigrades in response to the compounds, when compared to the control (log_2_FC = log_2_(1.5)). In the heatmaps, the rows represent the altered proteins and their relative expression levels under different conditions (control and 12.5 µM for **1′** and **2′**). The heatmap columns correspond to the three experimental conditions, and the colour gradient indicates variations in protein expression across different conditions.

For compound **1′**, proteomic analysis identified a total of 1562 proteins grouped into 1267 clusters. Out of the 1267 proteins, 622 were present in all replicates of at least one condition and, therefore, were considered for the analysis. A comparison between the exposed groups and the control group identified a total of 512 differentially expressed proteins (DEPs). The PCA (Figure 5a) of the 622 proteins included in the analysis, revealed a distinct separation between the control and the exposed group of compound **1′**. Although some variability could be observed among the replicates within the exposed group. The expression profile of the mussel plantigrades proteins altered after exposure to **1′**, showed a prevalence of downregulated DEPs, as illustrated in the volcano plot (Figure 5b). Furthermore, the volcano plot also highlighted two proteins with most altered expression, enolase (A0A8S3VIN2) with a FC = −6.64 and von Willbrand factor D (vWFD) domain-containing protein (A0A8B6G6F1) with a FC = 14.2. Among the DEPs were also identified other von Willebrand factors (vWF), epidermal growth factor-like (EGF) domains and TYR. Exposure to compound **1′** lead to the downregulation of three EGF-like forms (A0A8B6CPN1, A0A8B6DVI4, A0A8S3RV51), and TYR (EC 1.14.18.1) (A0A8B6FHC2). As mentioned before, **1′** upregulated one form of vWFD (A0A8B6G6F1), and altered the expression of two forms of vWFA (A0A8B6BU43, A0A8B6C9L4). Furthermore, a manual search was conducted on the DEPs for proteins associated with mussel adhesion, specifically mussel foot proteins, precollagens, and proximal thread matrix proteins. Nonetheless, none of the described proteins directly involved in the adhesion process were found among the DEPs.

A total of 791 proteins grouped into 480 clusters were identified through proteomic analysis of mussel plantigrades exposed to compound **2′**. Out of the 480 proteins, 119 were present in all replicates of at least one condition and, therefore, were considered for the analysis. The comparison between the exposed group and the control identified a total of 68 DEPs. A high variability was observed among the samples, particularly on 12.5 µM replicates, as seen in PCA which can be further verified by the proximity of replicate 1 from the exposed group to the control replicates on the heatmap (Figure 6a,c). The volcano plot showed a similar number of proteins upregulated and downregulated by compound **2′** (Figure 6b). The DEPs identified for **2′** included several proteins, among which were vWF proteins. The results showed the upregulation of one form of vWFA domain-containing protein (A0A8B6BV40). However, as observed for **1′**, no proteins directly involved in the adhesion process were found among the DEPs.

Among the identified DEPs, several were altered following exposure to both compounds. Notably, von Willebrand factor (vWF) domain-containing proteins were modulated by both **1′** and **2′**. Specifically, compound **1′** affected the expression of vWFD and vWFA proteins, while **2′** upregulated vWFA proteins. In vertebrates, vWF is a glycoprotein involved in haemostasis, promoting platelet adhesion at sites of vascular injury [78]. It contains four conserved domains: A, B, C, and D [79]. In mussels, however, vWF domain-containing proteins are believed to contribute to underwater adhesion [80,81], and shell mineralization [82]. Of particular interest are the two vWFA domains present in proximal thread matrix protein-1 (PTMP1), a non-collagenous matrix protein that plays a central role in the structural integrity of byssal threads [76,79]. PTMP likely contributes to thread stiffening through interactions between its vWFA domains and collagen [79,80,83]. Dysregulation of vWF domain-containing proteins upon exposure to the compounds may impair PTMP production, thereby compromising thread strength and potentially inhibiting mussel settlement.

Additionally, certain proteins were downregulated only by compound **1′**, such as EGF-like proteins and TYR. EGF-like domains are known components of mussel foot protein-2 (Mfp-2), a polyphenolic protein found across several mussel species [81,84,85,86,87]. Mfp-2 enhances adhesion resistance to enzymatic degradation and contributes to the stability of byssal plaques [76]. According to Hwang *et al*. [87], the EGF-like domains in Mfp-2 may also promote cohesion between the plaque and substratum. Therefore, the downregulation of these EGF-like proteins by **1′** could interfere with Mfp-2 production, potentially weakening plaque adhesion. TYR, as previously discussed, is a key enzyme involved in multiple physiological processes, including mussel adhesion. Its downregulation may reduce DOPA availability, thus impairing byssal thread curing and settlement. However, given that TYR activity was found to increase *in vitro* at the same concentrations, this may reflect a compensatory response by mussel plantigrades to elevated enzymatic activity. It is important to note that the enzymatic and proteomic approaches are complementary but not directly comparable. Interestingly, although both compounds induced similar increases in the *in vitro* TYR activity, TYR was not differentially expressed in the proteome of mussels exposed to compound **2′**. This discrepancy may suggest compound-specific regulatory effects at the transcriptional or post-translational level.

In summary, modulation of vWF domain-containing proteins was shared between **1′** and **2′**, whereas **1′** additionally affected EGF-like proteins. These proteins can be considered potential effectors of their AF activity on mussel plantigrades, as both are key constituents of adhesive proteins (PTMP and Mfp-2, respectively). Their modulation may therefore indirectly interfere with byssal thread formation and adhesion, supporting a putative mechanistic basis for the AF activity of **1′** and **2′**. In addition, TYR was identified as a DEP exclusively in samples exposed to **1′**. Collectively, these findings suggest that the structural modifications in **1′** and **2′** enhance their biological potency, enabling measurable proteomic disruption at lower concentrations. The results reinforce a structure–activity relationship in which subtle chemical substitutions modulate the specificity of AF mechanisms. However, a direct comparison of the molecular AF effects between carbonyl-substituted nucleosides (**1′** and **2′**) and their amino-substituted counterparts (**1** and **2**) is constrained by limitations in the proteomic datasets of the latter compounds. Specifically, compound **1** did not yield detectable DEPs at 12.5 µM, likely due to high biological variability, while proteomic analysis of compound **2** was conducted at a substantially higher concentration (50 µM versus 12.5 µM for **1′** and **2′**), limiting direct quantitative comparisons.

##### Functional Enrichment Analysis

A functional enrichment analysis was performed on the identified DEPs in *M. galloprovincialis* plantigrades exposed to **1′** and **2′**, using homologous sequences from *Magallana gigas* (formerly *Crassostrea gigas*) due to the absence of an annotated genome for this non-model organism in current g:Profiler databases. This approach enabled exploration of common molecular functions and biological processes impacted by compound exposure. The results of the BLASTp analysis for both compounds are presented in the (see Supplementary Tables S3 and S4). Homologous proteins were classified using gene ontology (GO) terminology and grouped according to their functions (see Supplementary Tables S5 and S6), using the g:OST function.

**Figure 7:**
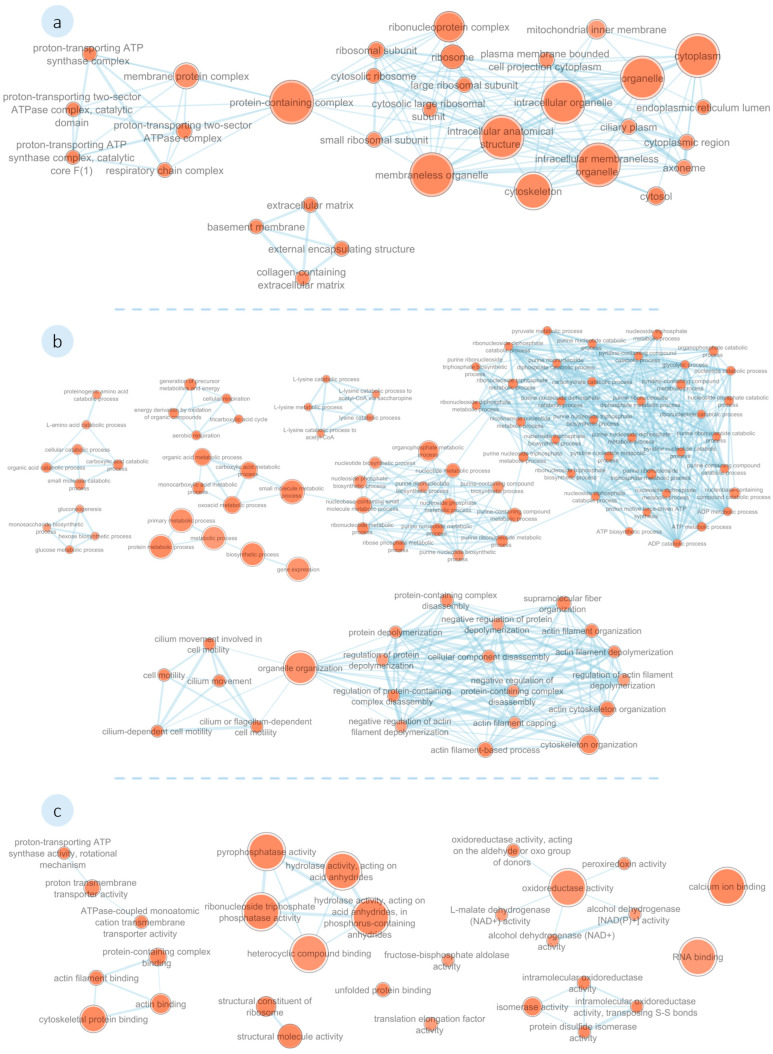
Functional network of differentially expressed proteins in the *M. galloprovincialis* plantigrades exposed to **1′**. Represented gene ontology (GO) categories are (**a**) Cellular Components, (**b**) Biological Processes, and (**c**) Molecular Functions. Node sizes increase with the number of proteins associated with the GO term and colour intensifies with significance (Node cut-off, *p* = 0.01).

The resulting functional network for **1′** is illustrated in Figure 7. The analysis yielded 257 GO terms that describe the functions of the differentially expressed proteins. Several proteins were found associated with ribosome constituents (e.g., Small ribosomal subunit protein uS15 (A0A8W8N791), Large ribosomal subunit protein uL13 (A0A8W8J403), 60S ribosomal protein L7a (A0A8W8L109), RRM domain-containing protein (A0A8W8J5R5), 40S ribosomal protein S3 (A0A8W8LEA1), Ribosomal protein L37 (A0A8W8IQU1), Small nuclear ribonucleoprotein Sm D2 (A0A8W8IE99), and 60 kDa SS-A/Ro ribonucleoprotein (K1QRQ0)), whereas other proteins were associated with cytoskeleton (e.g., Villin-1 (K1RGK4), Alpha-actinin, sarcomeric (K1RH58), Filamin-A (A0A8W8J8A2), Actin-depolymerizing factor 6 (D7EZG8), Fascin (K1QEZ3), Myosin-VI (K1Q1S3), Vinculin (A0A8W8MX28), and Collagen alpha-1(IV) chain (K1R1A5). Among these, were proteins involved in cilium movement (e.g., Tektin (A0A8W8IGH4), Radial spoke head protein 4-like protein A (A0A8W8IBB4), and Cilia- and flagella-associated protein 52 (K1RDT2)). Proteins putatively involved in ATP metabolism (e.g., ATP synthase subunit beta (A0A8W8MB85), 2-oxoglutarate dehydrogenase, mitochondrial (A0A8W8HLX2), Citrate synthase (A0A8W8MWR9), Cytochrome b-c1 complex subunit 6, mitochondrial (K1QKV1), Cytochrome c oxidase subunit 5B, mitochondrial (K1PZT2), Isocitrate dehydrogenase (A0A8W8HL81), Malate dehydrogenase (Fragment) (K1PU26), and Succinate dehydrogenase (A0A8W8N2K5)) and oxidoreductase activity (e.g., Acyl-coenzyme A oxidase (A0A8W8KG37), glutamate dehydrogenase (K1QRQ2), Glutathione S-transferase omega (A0A8W8K6J4), Heat shock 70 kDa protein 4L (A0A8W8KS31), Peroxidase (A0A8W8J0I5), Sulfide:quinone oxidoreductase, mitochondrial (K1QTY5), and Thioredoxin (A0A8W8L209)) were also identified.

**Figure 8:**
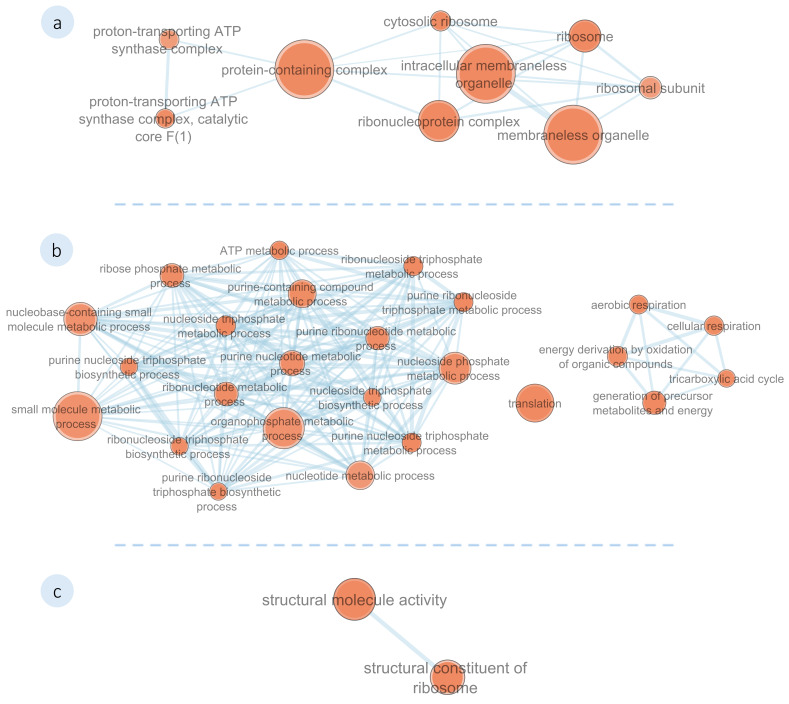
Functional network of differentially expressed proteins in the *M. galloprovincialis* plantigrades exposed to **2′**. Represented gene ontology (GO) categories are (**a**) Cellular Components, (**b**) Biological Processes, and (**c**) Molecular Functions. Node sizes increase with the number of proteins associated with the GO term and colour intensifies with significance (Node cut-off *p* = 0.01).

Figure 8 illustrates the functional enrichment of the mussel plantigrades exposed to compound **2′**. This analysis identified 140 GO terms associated with the functions of DEPs. Most of the identified proteins affected by **2′** have shown to be related to ribosome constituents (e.g., Histone H2B (K1R2L4), Large ribosomal subunit protein P1 (A0A8W8N7H9), Lamin Dm0 (K1RFA3), Ubiquitin-ribosomal protein eL40 fusion protein (K1PD36), 40S ribosomal protein S26 (A0A8W8LH25), Small ribosomal subunit protein uS2 (A0A8W8MQU1), and 60S acidic ribosomal protein P0 (K1QWX2)) and ATP metabolism (e.g., ATP synthase subunit gamma, mitochondrial (A0A8W8LM86), Nucleoside diphosphate kinase (K1RHA5), ATP-dependent 6-phosphofructokinase (A0A8W8NW87), Citrate synthase (A0A8W8MWR9), Cytochrome b-c1 complex subunit 6, mitochondrial (K1QKV1), Dihydrolipoyllysine-residue succinyltransferase component of 2-oxoglutarate dehydrogenase complex, mitochondrial (A0A8W8I7C9), Glucose-6-phosphate isomerase (K1PTI6), and Malate dehydrogenase, mitochondrial (A0A8W8K8K1)).

The analysis revealed a shared stress response to both compounds, with altered expression of proteins involved in metabolism, cell structure, and cellular stress responses. ATP metabolism was notably affected by both compounds. Compound **1′** downregulated key enzymes involved in ATP production, such as ATP synthase, citrate synthase, 2-oxoglutarate dehydrogenase, and malate dehydrogenase. In contrast, compound **2′** modulated their expression. Enzymes like citrate synthase, 2-oxoglutarate dehydrogenase, and malate dehydrogenase are essential for the citric acid cycle (Krebs cycle), generating electron carriers necessary for the mitochondrial electron transport chain [88]. ATP synthase, a critical component of the electron transport chain on the inner mitochondrial membrane, facilitates ATP synthesis through oxidative phosphorylation [88]. The downregulation of these proteins by **1′**, and the dysregulation observed with **2′**, suggests potential compensation mechanisms in response to mitochondrial dysfunction, increased oxidative stress, and metabolic dysregulation. Additionally, ciliary motility appeared to be affected by compound **1′**, which downregulated cilia-related proteins such as tektins and radial spoke proteins. Radial spoke proteins are involved in regulating ciliary motility [89], while tektins are structural components vital for the ciliary microtubule cytoskeleton. In mussels, cilia are essential for directing water flow for respiration and feeding [90,91], control the biointerface strength and stem release of the byssus [92]. Furthermore, in *Limnoperna fortune* foot, cilia have been suggested to assist in temporary adhesion [93]. Therefore, the downregulation of cilia-related proteins may indicate a defensive response of mussels to the compound, potentially affecting their overall health and physiological processes.

Taken together, these findings indicate that the AF activity of the tested compounds likely arises from a multifaceted mode of action involving both metabolic disruption and impairment of structural and adhesion-related functions. The observed interference with key components of energy metabolism suggests a reduction in ATP availability, which may compromise essential cellular processes and limit the energetic capacity required for active behaviors such as attachment, byssus production, and maintenance of cellular homeostasis. Concurrently, the downregulation of proteins associated with ciliary structure and function points to a direct impact on motility and surface interaction mechanisms, which are critical for effective substrate colonization and stable adhesion in fouling organisms. Importantly, these combined effects may not necessarily induce acute toxicity but rather weaken the organism’s physiological performance and its ability to successfully attach and persist on surfaces. This mode of action is particularly relevant in the context of environmentally friendly AF strategies, as it suggests a non-lethal interference with key biological processes rather than broad-spectrum toxicity. Overall, the proteomic alterations provide valuable mechanistic insight, highlighting how targeted disruption of energy metabolism and adhesion-related pathways can synergistically contribute to AF efficacy at the molecular level.

### 3.3. Predicted Environmental Behavior of the Compounds

#### 3.3.1. Toxicity Assessment of Target and Non-Target Organisms

In addition to effectively preventing the attachment and growth of fouling organisms, candidate AF agents must display a favourable environmental safety profile. Accordingly, the toxicity of compounds **1′** and **2′** was evaluated in both target and non-target species (Table 1). A therapeutic ratio (LC_50_/EC_50_) greater than 15 was used as a benchmark to assess efficacy relative to toxicity in mussel plantigrades [40]. For target organisms, including marine biofilm-forming bacteria, microalgae, and mussel plantigrades, no lethal effects were observed following exposure to either compound across the tested concentration ranges. In *Navicula* sp., although significant growth inhibition was detected, no evidence of cellular disruption or mortality was observed at any concentration. Consequently, LC_50_ values exceeding 200 µM were obtained for both compounds (**1′**: LC_50_ > 200 µM; >53.6 µg mL^−1^; **2′**: LC_50_ > 200 µM; > 50.4 µg mL^−1^), confirming their low toxicity towards this diatom. Similarly, mussel plantigrades exhibited no lethal effects upon exposure to either nucleoside, with LC_50_ values above 200 µM and therapeutic ratios well above the established threshold (LC_50_/EC_50_ > 33.99 for **1′** and >23.59 for **2′**). From a structure–activity relationship perspective, this non-toxic profile mirrors that previously reported for **1** and **2** (Table 2), indicating that substitution of the amino group by a carbonyl group within the purine moiety does not compromise environmental safety. This suggests that the conserved nucleoside scaffold, rather than specific substituents on the purine ring, is the dominant determinant of low toxicity.

**Figure 9:**
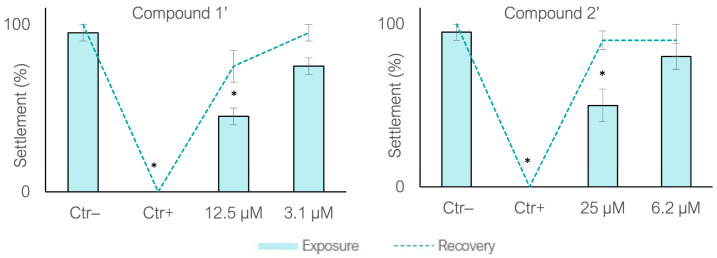
Settlement recovery bioassay. Solid bars indicate the exposure phase to the tested compounds, whereas dashed lines correspond to the subsequent recovery period in filtered natural seawater. Compound **1′** was assessed at concentrations of 3.1 and 12.5 µM. Compound **2′** was tested at concentrations of 6.2 and 25 µM. Ctr−: DMSO (0.1%). Ctr+: CuSO_4_ (5 µM). * Significant differences compared to the negative control (Dunnett’s test, *p* < 0.01).

To further investigate whether the observed settlement inhibition reflected acute toxicity or reversible biological effects, settlement recovery assays were performed using concentrations spanning the EC_50_ values (3.1–12.5 µM for **1′** and 6.2–25 µM for **2′**). Initial exposure resulted in significant settlement inhibition at the higher concentrations for both compounds (Dunnett’s test, *p* < 0.01; Figure 9). However, following transfer to clean filtered natural seawater, settlement levels were fully restored, with no significant differences relative to the control (Dunnett’s test, *p* < 0.01). Notably, settlement increased by approximately 30% for **1′** and 40% for **2′** at the higher concentrations. These results indicate the absence of acute toxicity and suggest that settlement inhibition is mediated by a reversible mechanism, consistent with previous observations for cyanobacterial portoamides [19]. Such reversibility further supports a mode of action based on temporary physiological modulation rather than irreversible cellular damage.

The effects of **1′** and **2′** on non-target organisms were also assessed using nauplii of the marine brine shrimp *A. salina* and the barnacle *A. amphitrite*. No mortality was observed in *A. amphitrite* nauplii across the tested concentration range (0.5–32 µM; 0.125–8 µg mL^−1^) or in *A. salina* nauplii at 25 and 50 µM (Dunnett’s test, *p* > 0.01) for either compound. These results support LC_50_ values greater than 32 µM for *A. amphitrite* and greater than 50 µM for *A. salina*, both well above the EC_50_ values determined for mussel plantigrades. Comparable toxicity thresholds were previously reported for compounds **1** and **2** (Table 2) in the same non-target species, further reinforcing the conclusion that nucleoside-based AF agents combine biological efficacy with a high degree of environmental compatibility.

#### 3.3.2. Persistence, Bioaccumulation and Adsorption Predictions

As complementary information to the ecotoxicological assessment, the physicochemical properties and predicted environmental fate of the four nucleosides (**1**, **2**, **1′**, and **2′**) were estimated using EPI Suite™ (Table 3). The predicted octanol–water partition coefficients (Log K_ow_) for **1′** and **2′** were well below the threshold of 4.5 established by European Chemicals Agency’s (ECHA) [94], indicating a low potential for bioaccumulation and sorption to organic matter. Notably, both compounds exhibited negative Log K_ow_, reflecting high water solubility, which is consistent with their overall polar nucleoside structures. Predicted soil adsorption coefficients (Log K_oc_) were also negative for all compounds, with values of −1.00 and −0.79 for **1′** and **2′**, respectively, further supporting low affinity for soils and sediments and, consequently, low environmental persistence in benthic compartments. Similar trends were observed for **1** and **2**, indicating that modifications at the purine 6-position (amino versus carbonyl group) do not substantially alter sorption behaviour, reinforcing a consistent SAR across the nucleoside series. Bioconcentration factors (BCF) were low for **1′** and **2′** (BCF = 3.16 L kg^−1^), remaining several orders of magnitude below the REACH Annex XIII threshold for bioaccumulative substances (BCF ≥ 2000 L kg^−1^) [94], which was in accordance with the observed for **1** and **2**. These values confirm the negligible potential for bioconcentration in aquatic organisms. Biodegradation potential was assessed using BIOWIN models, revealing distinct SAR trends linked to functional group substitutions. Compounds **1′** and **2′** were predicted to undergo rapid aerobic biodegradation (within days), whereas **1** and **2** were predicted to degrade over slightly longer timescales (days to weeks). This enhanced aerobic biodegradability of **1′** and **2′** may be associated with the presence of a carbonyl group at the 6-position of the purine ring, which could facilitate enzymatic attack. Under anaerobic conditions, compounds **1′** and **1** were predicted to degrade rapidly, while **2′** and **2** were not, suggesting that the presence of the 2′-hydroxyl group may play a role in degradation pathways under oxygen-limited conditions. Collectively, these predictions highlight how subtle structural differences within the nucleoside framework can modulate degradation behaviour without compromising overall environmental safety. Taken together, the negative Log K_ow_ and Log K_oc_ values indicate high mobility in aquatic environments; however, the rapid predicted biodegradation suggests a short environmental residence time, particularly in biological systems. Consequently, **1′** and **2′** present a low concern in terms of persistence, bioaccumulation, and long-term ecological impact. While expanding ecotoxicological testing to additional non-target species would further strengthen risk assessment, the predicted PBT profiles strongly support the suitability of these nucleosides as environmentally compatible AF agents for marine coating applications.

## 4. Conclusions

Two nucleoside analogues, hypoxanthine arabinoside (**1′**) and 2’-deoxyinosine (**2′**), derived from the AF-active nucleosides adenosine (**1**) and 2’-deoxyadenosine (**2**), were investigated to elucidate the influence of substituting the amino group on the purine base with a carbonyl group on AF performance and environmental behaviour. The analogues were assessed for AF efficacy against representative micro- and macrofouling organisms, molecular mechanisms of action in *M. galloprovincialis* plantigrades, and ecotoxicological effects and environmental fate.

Both compounds retained AF efficacy, significantly inhibiting diatom growth and mussel plantigrade settlement, without inducing lethality in target fouling organisms. Neither compound affected marine biofilm-forming bacteria, indicating that the structural modification preserved the selectivity of nucleosides toward eukaryotic fouling taxa. Mechanistic studies revealed modulation of AChE and TYR activity *in vitro*, suggesting involvement of neurotransmission and byssal thread formation-related pathways in the observed AF effects. Molecular docking studies confirmed high binding affinity to AChE, while TYR modulation—absent for compounds **1** and **2**—appears to be associated with the carbonyl substitution on the purine base.

Proteomic analysis of exposed mussel plantigrades revealed modulation of vWF domain-containing proteins for both analogues, together with compound-specific molecular responses. Compound **1′** additionally induced downregulation of EGF-like proteins and TYR, whereas **2′** selectively upregulated vWFA proteins. Functional enrichment analysis further indicated shared effects on ATP metabolism, while **1′** exerted additional influence on proteins associated with ciliary function and oxidoreductase activity. These results demonstrate that while overall AF efficacy is preserved, and subtle chemical modifications can alter the magnitude and specificity of molecular responses. From an ecotoxicological perspective, both compounds exhibited a favourable environmental profile. No lethal effects were observed in non-target species (*A. salina* and *A. amphitrite* nauplii), and environmental fate modelling predicted low persistence, bioaccumulation, and bioconcentration potential, combined with high water solubility and rapid degradation. This benign profile closely mirrors that of the parent nucleosides, indicating that carbonyl substitution does not compromise environmental safety.

Overall, this study demonstrates that nucleoside-mediated AF activity is not dependent on the presence of an amino functional group on the purine base, although structural modifications can influence the underlying molecular mechanisms. By integrating chemical structure–activity relationships with mechanistic and ecotoxicological endpoints, these findings support nucleosides as a promising class of environmentally compatible AF agents and provide a framework for their further development as low-impact alternatives to conventional biocidal coatings.

## Figures and Tables

**Table 1:** Antifouling effectiveness *versus* toxicity of compounds **1′** and **2′** against biofouling species. The half maximum effective concentration (EC_50_), the median lethal concentration (LC_50_), and the therapeutic ratio (LC_50_/EC_50_) are represented. The EC_50_ and LC_50_ are provided in both µM and µg mL^−1^ for comparative purposes. Confidence intervals for EC_50_ values are in µM (in parentheses). **1′**: hypoxanthine arabinoside; **2′**: 2’-deoxyinosine. n.a.—not assessed.

	Target Species	EC_50_ (µM; µg mL^−1^)	LC_50_ (µM; µg mL^−1^)	LC_50_/EC_50_
**1′**	*M. galloprovincialis*	5.50 (95% CI: 2.85—8.50); 1.47	>200; >53.60	>36.38
	*Navicula* sp.	n.a.		n.a.
**2′**	*M. galloprovincialis*	8.54 (95% CI: 3.54–14.95); 2.15	>200; >50.40	>23.41
	*Navicula* sp.	n.a.		n.a.

**Table 2:** Comparative toxicity of compounds **1′** and **2′** with **1** and **2** against target and non-target organisms. The median lethal concentration (LC_50_) is represented in both µM and µg mL^−1^ for comparative purposes. **1′**: hypoxanthine arabinoside; **1**: adenosine; **2′**: 2’-deoxyinosine; **2**: 2’deoxyadenosine.

Compounds	LC_50_ (µM; µg mL^−1^)				
	Target Species			Non-Target Species	
	*Marine biolfilm-forming bacteria*	*Navicula* sp.	*M. galloprovincialis*	*A. salina*	*A. amphithrite*
**1′**	>50; 13.40	>200; >53.60	>200; >53.60	>50; 13.40	>32; >8
**2′**	>50; 12.60	>200; >50.40	>200; >50.40	>50; 12.60	>32; >8
**1** [9]	>50; 13.36	>200; 53.45	>200; 53.45	>50; 13.36	>32; >8
**2** [9]	>50; 12.56	>200; 50.25	>200; 50.25	>50; 12.56	>32; >8

**Table 3:** Predicted data from EPI Suite™. Includes biodegradability, sediment-water partition (Log K_oc_), octanol-water partition (Log K_ow_), bioconcentration factor (BCF), and bioaccumulation factor (BAF) of **1′** (hypoxanthine arabinoside) and **2′** (2’-deoxyinosine). BIOWIN™ criteria: (3) predicted time for ultimate biodegradation; (4) primary biodegradation in aerobic conditions; (5) MITI linear model prediction; and (7) predicted probability for fast biodegradation in anaerobic conditions. Results for (3) and (4): values 5.00 = hours, 4.00 = days, 3.00 = weeks, 2.00 = months, 1.00 = recalcitrant. Results for (5) and (7): values < 0.5 = does not biodegrade fast. ^a^ RB: readily biodegradable; ^b^ BF: Biodegrades Fast; ^c^ DNBF: Does not biodegrade fast.

	BIOWIN™				KOCWIN™	KOWWIN™	BCFBAF™
Compound	Ultimate Biodegradation (3)	Aerobic Conditions (4)	MITI Linear Model (5)	Anaerobic Conditions (7)	Log K_oc_	Log K_ow_	BCF (LKg/Wet-Wt)
**1′**	Weeks (3.02)	Days (4.04)	RB ^a^ (0.64)	BF ^b^ (0.54)	−1.00	−1.84	3.16
**2′**	Weeks (2.90)	Days (3.94)	RB ^a^ (0.54)	DNBF ^c^ (0.24)	−0.79	−1.18	3.16

## Data Availability

The original contributions presented in this study are included in the article/Supplementary Material. Further inquiries can be directed to the corresponding author.

## Supplementary Materials

The following supporting information can be downloaded at: https://www.mdpi.com/article/10.3390/biom16040584/s1, Table S1. List of differentially expressed proteins (DEPs) in *Mytilus galloprovincialis* plantigrades exposed to hypoxanthine arabinoside (**1′**). Table S2. List of differentially expressed proteins (DEPs) in *Mytilus galloprovincialis* plantigrades exposed to 2’-deoxyinosine (**2′**). Table S3. Results of BLASTp analysis for DEPs identified in response to hypoxanthine arabinoside (**1′**). Table S4. Results of BLASTp analysis for DEPs identified in response to 2’-deoxyinosine (**2′**). Table S5. Functional analysis of DEPs identified in response to hypoxanthine arabinoside (**1′**). Table S6. Functional analysis of DEPs identified in response to 2’-deoxyinosine (**2′**).
