# Supplementary material for: Structure–Activity Relationships, Molecular Mechanisms, and Ecotoxicological Evaluation Underlying Nucleoside-Mediated Antifouling Activity
Source: Biomolecules. 2026 Apr 14;16(4):584. doi: 10.3390/biom16040584 (PMC13114020; doi:10.3390/biom16040584)
Supplement: Supplementary file 1 [file biomolecules-16-00584-s001.zip › biomolecules-4211347-supplementary.pdf]

biomolecules

MDPI

Ariticle

Structure–Activity Relationships. Molecular Mechanisms. and Ecotoxicological Evaluation Underlying Nucleoside-Mediated Antifouling Activity

Sandra Pereira [1,2], Isabel B. Oliveira [1], Andreia Palmeira [3], Maria Turkina [4], Vitor Vasconcelos [1,2], Alexandre Campos [1] and Joana R. Almeida [1*]

[1] CIIMAR/CIMAR LA. Interdisciplinary Centre of Marine and Environmental Research. University of Porto. Terminal de Cruzeiros do Porto de Leixões. 4450-208 Matosinhos. Portugal.
[2] Biology Department. Faculty of Sciences. University of Porto. Rua do Campo Alegre. 4169-007 Porto. Portugal
[3] Laboratory of Organic and Pharmaceutical Chemistry. Department of Chemical Sciences. Faculty of Pharmacy. University of Porto. Rua Viterbo Ferreira. 228. 4050-313. Porto. Portugal
[4] Department of Biomedical and Clinical Sciences. Faculty of Medicine and Clinical Sciences. Linköping University. 581 83. Linköping. Sweden
Correspondence: jalmeida@ciimar.up.pt

Table S1. List of differentially expressed proteins (DEPs) in *Mytilus galloprovincialis* plantigrades exposed to hypoxanthine arabinoside (**1′**). DEPs were obtained from pairwise comparisons between the control group (0.1% DMSO) and exposed group at 12.5 µM. using a threshold of lfc = $log_2(1.5)$. Represented are the relative expression of DEPs on the control group (Ctr-) and the exposed group (12.5 µM). and the obtained fold change of each DEP.

Reference sequence	Protein name	Relative expression		Fold Change
		Ctr-	12.5 µM	
A0A8B6C5G7	Acetyl-CoA acyltransferase 2 (EC 2.3.1.16)	1.56	-1.56	-3.12
A0A8B6F498	Acyl-coenzyme A oxidase	0.64	-0.64	-1.28
A0A8B6DSL8	Adenosylhomocysteinase (EC 3.13.2.1)	0.61	-0.61	-1.21
A0A8S3SJS5	CAP1_2	1.24	-1.24	-2.47
A0A8S3S1H7	Adk (EC 2.7.4.3)	0.50	-0.50	-1.00
A0A8S3TZ88	Adk (EC 2.7.4.3)	0.87	-0.87	-1.73
A0A8B6D0Q7	ADP-ribosylation factor 1	0.35	-0.35	-0.70
A0A8B6C281	Advillin	1.26	-1.26	-2.53
A0A8B6EIW3	alanine transaminase (EC 2.6.1.2)	1.13	-1.13	-2.26
A0A8B6BGG4	Alpha-aminoadipic semialdehyde synthase (EC 1.5.1.8)	0.64	-0.64	-1.28
A0A8B6D196	Aminopeptidase (EC 3.4.11.-)	1.05	-1.05	-2.10
A0A8S3QGC9	Annexin	0.44	-0.44	-0.87
A0A8S3VH93	Antistasin-like domain-containing protein	2.05	-2.05	-4.09
A0A8S3QFL2	AP complex subunit beta	0.34	-0.34	-0.69
A0A8S3S184	Apextrin C-terminal domain-containing protein	1.07	-1.07	-2.14
A0A8B6BZ74	Apoptosis inhibitor 5	0.47	-0.47	-0.94
A0A8B6D0T5	Apple domain-containing protein	0.81	-0.81	-1.63
A0A8S3TG22	AQP4	1.37	-1.37	-2.74
A0A8S3VBZ6	ARHGDI	0.48	-0.48	-0.96
A0A8B6H8N6	Asparaginyl-tRNA synthetase (EC 6.1.1.22)	1.15	-1.15	-2.29

A0A8S3SXM2	ATP-dependent 6-phosphofructokinase (ATP-PFK) (Phosphofructokinase) (EC 2.7.1.11) (Phosphohexokinase)	-0.34	0.34	0.68
A0A8B6GBE6	ATP synthase-coupling factor 6. mitochondrial	1.19	-1.19	-2.37
A0A8B6H7T7	ATP synthase subunit beta (EC 7.1.2.2)	1.39	-1.39	-2.78
A0A077GYU5	ATP synthase subunit d. mitochondrial	1.10	-1.10	-2.19
A0A8B6GU32	ATP synthase subunit gamma	0.55	-0.55	-1.09
A0A6J8F3Y7	ATP synthase subunit O. mitochondrial (Oligomycin sensitivity conferral protein)	-0.71	0.71	1.41
A0A6J8C7Z5	ATPeF0F	2.34	-2.34	-4.67
A0A8S3Q2E4	ATPeF1D	1.74	-1.74	-3.47
A0A6J8DY85	ATPeV1E	1.29	-1.29	-2.57
A0A8B6BPI1	B box-type domain-containing protein	1.40	-1.40	-2.79
A0A8B6F754	BPTI/Kunitz inhibitor domain-containing protein	1.40	-1.40	-2.81
A0A8B6DQ42	C-type lectin domain-containing protein	1.46	-1.46	-2.91
A0A8B6GW86	C1q domain-containing protein	1.78	-1.78	-3.55
A0A6J8E3N6	C1q domain-containing protein	1.72	-1.72	-3.44
A0A8B6HA48	C2 domain-containing protein	-0.54	0.54	1.08
A0A8S3SGA7	Cadherin domain-containing protein	-1.03	1.03	2.06
A0A8B6FBT9	Calmodulin	1.01	-1.01	-2.03
A0A8S3R7D4	Calreticulin	0.74	-0.74	-1.48
A0A8B6DWN7	cAMP-dependent protein kinase regulator	0.93	-0.93	-1.85
A0A8B6D440	Cathepsin D (EC 3.4.23.5)	0.50	-0.50	-1.01
A0A8B6GVE1	CD80-like immunoglobulin C2-set domain-containing protein	0.61	-0.61	-1.22
A0A8S3URK5	CDY (EC 2.3.1.48)	1.76	-1.76	-3.53
A0A8B6FIN1	Chitin-binding type-2 domain-containing protein	1.46	-1.46	-2.92
A0A8B6DBL3	Chitin-binding type-2 domain-containing protein	1.38	-1.38	-2.77
A0A8S3T4F4	Choline transporter-like protein	0.51	-0.51	-1.02
A0A8B6F3H7	Cilia- and flagella-associated protein 161	1.31	-1.31	-2.62
A0A8B6GX94	Clathrin heavy chain	0.73	-0.73	-1.45
A0A6J8ESY4	Small ribosomal subunit protein uS15 (40S ribosomal protein S13)	1.34	-1.34	-2.67
A0A6J8EHM6	Small ribosomal subunit protein uS5 (40S ribosomal protein S2)	0.98	-0.98	-1.95
A0A8B6G7J5	Large ribosomal subunit protein uL13 (60S ribosomal protein L13a)	1.54	-1.54	-3.08
A0A077H0N7	60S ribosomal protein L7a	1.08	-1.08	-2.16
Q9Y0D6	Actin	-0.52	0.52	1.04
A0A8S3Q5J1	ACTN1_4	1.05	-1.05	-2.10
A0A8B6GV47	ADP/ATP translocase (ADP.ATP carrier protein)	1.56	-1.56	-3.12
A0A8B6D2T4	ADP-ribosylation factor	2.09	-2.09	-4.19
A0A8B6GKE0	Aldehyde dehydrogenase (NAD+) (EC 1.2.1.3)	0.87	-0.87	-1.73
A0A8S3T4E0	ALDH (EC 1.2.1.3)	1.03	-1.03	-2.07
A0A8B6CW94	Alpha-1.4 glucan phosphorylase (EC 2.4.1.1)	0.34	-0.34	-0.68
A0A8B6DXX6	Apextrin C-terminal domain-containing protein	1.17	-1.17	-2.34
A0A8B6HT02	Armadillo repeat-containing protein 4	-0.34	0.34	0.67
A0A8B6CRC1	ATP synthase subunit alpha	1.56	-1.56	-3.12
A0A6J8EBJ6	Band 7 domain-containing protein	1.37	-1.37	-2.73
A0A8B6GBK5	C-1-tetrahydrofolate synthase. cytoplasmic (EC 1.5.1.5) (EC 3.5.4.9) (EC 6.3.4.3)	0.48	-0.48	-0.95
A0A8B6FVI3	C1q domain-containing protein	0.81	-0.81	-1.61
A0A8B6G4E0	Calcium-transporting ATPase (EC 7.2.2.10)	0.69	-0.69	-1.38
A0A8B6E763	Calmodulin	0.82	-0.82	-1.63
A0A8S3QR75	CAPNN (EC 3.4.22.-)	1.00	-1.00	-2.00
A0A8B6DHE3	Cathepsin B (EC 3.4.22.1)	0.53	-0.53	-1.06
A0A6J8A856	CEP135	0.61	-0.61	-1.22

A0A8B6EZW6	Cilia- and flagella-associated protein 45	1.16	-1.16	-2.32
A0A8B6C153	Citrate synthase	0.95	-0.95	-1.90
A0A8B6CP95	Collagen. type IV. alpha	0.34	-0.34	-0.68
A0A8B6FHM0	Collagen. type XIII. alpha	-0.68	0.68	1.35
A0A8S3UY90	CROCC	0.64	-0.64	-1.28
A0A8B6C6M2	Crystallin. alpha B	0.90	-0.90	-1.81
A0A6J8D0X9	Fibropellin-1	-0.93	0.93	1.86
A0A8S3QII9	CTSL (EC 3.4.22.15)	0.93	-0.93	-1.86
A0A8B6D685	Dihydrolipoyllysine-residue succinyltransferase component of 2-oxoglutarate dehydrogenase complex. mitochondrial (EC 2.3.1.61) (2-oxoglutarate dehydrogenase complex component E2) (E2K)	0.51	-0.51	-1.01
A0A8S3RHZ5	DLG1	0.54	-0.54	-1.08
A0A8B6DX16	Dolichyl-diphosphooligosaccharide--protein glycosyltransferase subunit 2 (Ribophorin-2)	0.47	-0.47	-0.95
A0A8B6FGA8	Dynein heavy chain. axonemal	-1.52	1.52	3.04
A0A8S3RF13	Transketolase (EC 2.2.1.1)	1.43	-1.43	-2.86
A0A8S3R8F7	E2.7.3.3 (EC 2.7.3.3)	1.15	-1.15	-2.31
A0A6J8A1B7	EEF1A	1.02	-1.02	-2.04
A0A8B6EQ50	EF-hand domain-containing protein	0.62	-0.62	-1.24
A0A8B6CPN1	EGF-like domain-containing protein	1.08	-1.08	-2.15
A0A8B6FH66	Elongation factor 1-gamma	2.40	-2.40	-4.79
A0A8B6EYD8	Elongation factor 2	0.98	-0.98	-1.97
A0A8B6FQU1	Erythrocyte membrane protein band 4.1	-0.85	0.85	1.69
A0A8B6FTA2	Far upstream element-binding protein	1.48	-1.48	-2.96
A0A8B6GX54	Fibulin-1	0.44	-0.44	-0.89
A0A8B6H907	Filamin	-1.98	1.98	3.96
A0A8B6DL75	Filamin	0.59	-0.59	-1.17
A0A8B6E885	Gelsolin-like domain-containing protein	1.40	-1.40	-2.80
A0A8B6BF20	Glutathione S-transferase (EC 2.5.1.18)	1.52	-1.52	-3.04
A0A8B6DZ81	Glyceraldehyde-3-phosphate dehydrogenase (EC 1.2.1.12)	0.79	-0.79	-1.57
A0A8B6DYX8	Heat shock 70kDa protein 5	0.43	-0.43	-0.86
C0Z203	Heat shock protein 90	1.07	-1.07	-2.13
A0A8B6BR27	Heat shock protein 90kDa beta	0.77	-0.77	-1.53
A0A8B6GHQ6	Hemicentin	0.88	-0.88	-1.75
A0A8B6H9V2	Hemicentin	0.39	-0.39	-0.79
A0A0K0YAY3	Heparan sulfate proteoglycan-like protein-1	0.58	-0.58	-1.15
A0A8B6EPK5	Heterogeneous nuclear ribonucleoprotein R	1.51	-1.51	-3.02
A0A0L8GYE0	Histone H4	1.37	-1.37	-2.73
A0A8S3VDI8	HSD17B4 (EC 1.1.1.-. EC 4.2.1.107. EC 4.2.1.119)	0.55	-0.55	-1.11
A0A8B6CAW3	K Homology domain-containing protein	0.63	-0.63	-1.25
A0A8B6BT14	Large subunit ribosomal protein L4e	1.38	-1.38	-2.76
A0A8S3QSD1	LDB3	1.23	-1.23	-2.46
A0A8B6EFR5	Leucyl aminopeptidase (EC 3.4.11.1)	0.99	-0.99	-1.98
A0A8B6GXC9	Uncharacterized protein	0.74	-0.74	-1.47
A0A8S3VFX6	LIM zinc-binding domain-containing protein	0.66	-0.66	-1.32
A0A8S3PRL2	LLGL	0.45	-0.45	-0.90
A0A8S3Q6R0	LMNB	1.20	-1.20	-2.40
A0A8S3TFK4	Malate dehydrogenase (EC 1.1.1.37)	0.85	-0.85	-1.71
A0A8B6C2Y2	Malate dehydrogenase (EC 1.1.1.37)	1.24	-1.24	-2.48
A0A8B6DBF4	Metalloendopeptidase (EC 3.4.24.-)	0.93	-0.93	-1.86
A0A8B6FW38	Molecular chaperone DnaK	0.96	-0.96	-1.92

A0A8B6F2T3	Nesprin-1	-1.21	1.21	2.41
A0A8B6EEA4	Neural cell adhesion molecule	-0.31	0.31	0.62
A0A8B6HFZ0	non-specific serine/threonine protein kinase (EC 2.7.11.1)	0.37	-0.37	-0.75
A0A8B6ETB5	Nucleolin	1.33	-1.33	-2.66
A0A8B6DGA0	Outer dense fiber protein 3	0.99	-0.99	-1.98
A0A8B6D9K2	P-type domain-containing protein	1.54	-1.54	-3.08
A0A8S3RZQ4	Parkin coregulated gene protein homolog.Parkin coregulated gene protein	2.29	-2.29	-4.58
A0A8B6DTM2	PDZ domain-containing protein	0.66	-0.66	-1.32
A0A6J8B435	peptidylglycine monooxygenase (EC 1.14.17.3)	-0.47	0.47	0.95
A0A8B6F8H6	Peroxiredoxin 6. 1-Cys peroxiredoxin (EC 1.11.1.7)	1.13	-1.13	-2.25
A0A8B6FIW7	Solute carrier family 25 (Mitochondrial phosphate transporter). member 3	1.98	-1.98	-3.96
A0A8B6CG53	phosphoenolpyruvate carboxykinase (GTP) (EC 4.1.1.32)	0.60	-0.60	-1.19
A0A8B6HP01	phosphoenolpyruvate mutase (EC 5.4.2.9)	1.31	-1.31	-2.61
A0A8B6C763	Plastin-3	0.81	-0.81	-1.62
A0A8B6CYA7	Poly [ADP-ribose] polymerase (PARP) (EC 2.4.2.-)	-0.73	0.73	1.47
A0A8S3QR29	thioredoxin-dependent peroxiredoxin (EC 1.11.1.24)	1.35	-1.35	-2.70
A0A8B6BJW5	Prohibitin	1.12	-1.12	-2.25
A0A8B6DIX4	Prostaglandin-H2 D-isomerase / glutathione transferase (EC 5.3.99.2)	1.48	-1.48	-2.96
A0A8B6EJ02	Protein disulfide-isomerase (EC 5.3.4.1)	0.67	-0.67	-1.33
A0A8B6G1S8	Protein disulfide-isomerase (EC 5.3.4.1)	-0.40	0.40	0.80
A0A6J8BIG8	Small ribosomal subunit protein RACK1	0.79	-0.79	-1.58
A0A8B6D4L6	Radixin	1.30	-1.30	-2.60
A0A3G1CJN6	Ribosomal protein S3a	0.74	-0.74	-1.49
A0A8B6C4T1	RNA helicase (EC 3.6.4.13)	1.36	-1.36	-2.71
A0A8B6DTX0	Rootletin	0.85	-0.85	-1.71
A0A8B6BF70	SHSP domain-containing protein	0.90	-0.90	-1.80
A0A8B6DCE2	40S ribosomal protein S3	-0.39	0.39	0.78
A0A8S3QQF6	Sodium/potassium-transporting ATPase subunit alpha	1.26	-1.26	-2.52
A0A126Q9D5	Staphylococcal nuclease domain-containing protein 1	0.93	-0.93	-1.86
A0A6J8B610	Stress-induced-phosphoprotein 1	0.61	-0.61	-1.21
A0A6J8DUP3	STOM	0.73	-0.73	-1.45
A0A8S3TEG9	Succinate dehydrogenase [ubiquinone] flavoprotein subunit. mitochondrial (EC 1.3.5.1)	1.92	-1.92	-3.84
A0A6J8ESE0	TAGLN	1.07	-1.07	-2.14
A0A8B6GHP0	Tektin	0.84	-0.84	-1.68
A0A8B6DFA7	Thymosin beta	0.41	-0.41	-0.83
Q966V3	Transgelin	0.52	-0.52	-1.03
A0A8B6H439	Transglutaminase-like domain-containing protein	0.83	-0.83	-1.65
A0A6J8BFQ0	Trichohyalin-plectin-homology domain-containing protein	0.30	-0.30	-0.60
A0A8B6BTY3	Triosephosphate isomerase (EC 5.3.1.1)	2.12	-2.12	-4.25
A0A8B6F8B1	Tropomyosin	0.79	-0.79	-1.57
A0A3S1A1H2	Tubulin alpha chain	0.67	-0.67	-1.33
A0A8B6BWE2	Tubulin beta chain	0.44	-0.44	-0.87
A0A6J8CNX6	Uncharacterized protein	1.33	-1.33	-2.66
A0A8B6BL03	Ubiquinol-cytochrome c reductase core subunit 2	0.81	-0.81	-1.63
A0A8B6HIV7	Uncharacterized protein	1.02	-1.02	-2.04
A0A8B6EIG0	Uncharacterized protein	1.25	-1.25	-2.49
A0A8B6DFD7	Uncharacterized protein	0.60	-0.60	-1.20
A0A8B6G096	Uncharacterized protein	0.52	-0.52	-1.04
A0A8B6E104	Uncharacterized protein	2.01	-2.01	-4.01
A0A8B6EM75	Uncharacterized protein	1.52	-1.52	-3.04

A0A8B6C479	Uncharacterized protein	0.32	-0.32	-0.63
A0A6J8CV26	Uncharacterized protein	0.43	-0.43	-0.86
A0A8B6E647	Uncharacterized protein	-1.44	1.44	2.89
A0A8B6GX65	Uncharacterized protein	-0.62	0.62	1.24
A0A6J8AGA3	Uncharacterized protein	0.96	-0.96	-1.91
A0A8S3SZC2	Choice-of-anchor I domain-containing protein	0.43	-0.43	-0.86
A0A8B6DPQ0	SCO-spondin	0.33	-0.33	-0.67
A0A8B6DC97	MAM domain-containing protein	0.70	-0.70	-1.39
A0A6J8BUN8	Uncharacterized protein	0.79	-0.79	-1.57
A0A8B6GIV6	Mammalian ependymin-related protein 1	0.75	-0.75	-1.50
A0A8B6G9E5	Uncharacterized protein	0.48	-0.48	-0.96
A0A8B6CB58	Uncharacterized protein	1.25	-1.25	-2.50
A0A8B6BI55	Vinculin	1.94	-1.94	-3.88
A0A8B6BU43	VWFA domain-containing protein	-1.69	1.69	3.38
A0A8S3TBN4	YBX1	2.18	-2.18	-4.36
A0A8S3PR18	YWHAB_Q_Z	0.75	-0.75	-1.49
A0A8B6EBM4	Coatomer subunit alpha	0.43	-0.43	-0.87
A0A8S3VPZ7	Coatomer subunit delta	-0.56	0.56	1.12
A0A8B6CVU1	Cofilin	1.04	-1.04	-2.07
A0A8B6BSQ3	Coiled-coil domain-containing protein 170	-0.33	0.33	0.66
A0A8B6FD83	Collagen. type II. alpha	-0.40	0.40	0.79
A0A8B6EZ83	Collagen. type IV. alpha	0.56	-0.56	-1.12
A0A8B6CQJ1	Copper acquisition factor BIM1-like domain-containing protein	0.87	-0.87	-1.74
A0A8B6CAW5	Copper acquisition factor BIM1-like domain-containing protein	0.40	-0.40	-0.81
A0A8S3Q3L3	CRISPLD	0.71	-0.71	-1.42
A0A6J8BMS7	CTTN	0.51	-0.51	-1.02
A0A8S3RLA1	Uncharacterized protein	1.00	-1.00	-1.99
A0A8S3RUL4	cystathionine gamma-lyase (EC 4.4.1.1) (Gamma-cystathionase)	0.64	-0.64	-1.29
A0A8B6HEV7	Cysteine and glycine-rich protein	2.29	-2.29	-4.57
A0A6J8AEC3	Cysteine-rich with EGF-like domain protein 2-B.Cysteine-rich with EGF-like domain protein 2-A.Cysteine-rich with EGF-like domain protein 2.Cysteine-rich with EGF-like domain protein 1	1.08	-1.08	-2.17
A0A8B6F031	Cytochrome b-c1 complex subunit 6	0.48	-0.48	-0.97
A0A8B6G281	Cytochrome c oxidase subunit 5b	2.29	-2.29	-4.58
A0A8B6EWU5	Cytosolic fatty-acid binding proteins domain-containing protein	0.79	-0.79	-1.57
A0A8B6H151	Death-associated protein 1	1.52	-1.52	-3.03
A0A077H0R0	Deltamethrin resistance protein prag01 domain-containing protein	0.78	-0.78	-1.56
A0A8B6GTY8	Trans-1.2-dihydrobenzene-1.2-diol dehydrogenase (EC 1.1.1.179) (EC 1.3.1.20) (D-xylose 1-dehydrogenase) (D-xylose-NADP dehydrogenase) (Dimeric dihydrodiol dehydrogenase)	1.46	-1.46	-2.93
A0A8B6DQH5	Dihydrolipoyl dehydrogenase (EC 1.8.1.4)	0.76	-0.76	-1.52
A0A6J8BTF0	DNALI	0.94	-0.94	-1.88
A0A8B6BL12	Dolichyl-diphosphooligosaccharide--protein glycosyltransferase subunit DAD1 (Oligosaccharyl transferase subunit DAD1)	2.33	-2.33	-4.65
A0A8B6H0X9	Drebrin-like protein	0.87	-0.87	-1.74
A0A8B6FMV8	Secreted protein	0.51	-0.51	-1.01
A0A6J8DUW4	Uncharacterized protein	0.83	-0.83	-1.67
A0A6J8BZ13	Dynein light intermediate chain	0.89	-0.89	-1.77
A0A8B6G9Z7	E1 ubiquitin-activating enzyme (EC 6.2.1.45) (Ubiquitin-activating enzyme E1)	0.77	-0.77	-1.53
A0A8B6EYR0	EF-hand domain-containing family member C2	1.58	-1.58	-3.17
A0A8B6CHC6	EF-hand domain-containing protein	0.45	-0.45	-0.90

A0A3G1CJM7	EF-hand domain-containing protein	0.85	-0.85	-1.70
A0A3R5Q145	EF-hand domain-containing protein	1.49	-1.49	-2.98
A0A8B6GTM1	EF-hand domain-containing protein	0.70	-0.70	-1.39
A0A8B6BHU6	EF-hand domain-containing protein	1.13	-1.13	-2.26
A0A8B6EJE1	EF-hand domain-containing protein	1.46	-1.46	-2.91
A0A8B6DVI4	EGF-like domain-containing protein	0.94	-0.94	-1.87
A0A8S3RV51	EGF-like domain-containing protein	0.87	-0.87	-1.74
A0A6J8CPT2	Electron transfer flavoprotein subunit alpha (Alpha-ETF)	1.09	-1.09	-2.18
A0A8S3V871	EML1_2	1.06	-1.06	-2.13
A0A8B6C964	Endonuclease/exonuclease/phosphatase domain-containing protein	0.98	-0.98	-1.95
A0A8B6H228	Endoplasmic reticulum resident protein 44	0.79	-0.79	-1.59
A0A8S3VIN2	Enolase	3.32	-3.32	-6.64
A0A8B6CMG1	enoyl-CoA hydratase (EC 4.2.1.17)	0.79	-0.79	-1.57
A0A8B6GL66	Epidermal growth factor receptor kinase substrate 8	0.33	-0.33	-0.66
A0A6J8D3C7	ERLIN	0.61	-0.61	-1.22
A0A6J8EG86	Eukaryotic translation initiation factor 3 subunit B (eIF3b) (Eukaryotic translation initiation factor 3 subunit 9)	0.62	-0.62	-1.25
A0A8S3RJH3	Eukaryotic translation initiation factor 3 subunit I (eIF3i)	1.23	-1.23	-2.46
A0A8S3PS95	Eukaryotic translation initiation factor 5A (eIF-5A)	1.50	-1.50	-3.00
A0A8S3VLQ5	F-actin-capping protein subunit alpha	2.22	-2.22	-4.43
A0A8B6DPU0	Cytosolic fatty-acid binding proteins domain-containing protein	1.75	-1.75	-3.51
A0A8B6DQX4	Lipocalin/cytosolic fatty-acid binding domain-containing protein	1.65	-1.65	-3.31
A0A8B6BMA1	Uncharacterized protein	1.23	-1.23	-2.45
A0A8B6E270	FAS1 domain-containing protein	1.64	-1.64	-3.28
A0A8B6F6T7	FAS1 domain-containing protein	1.18	-1.18	-2.36
A0A6J8D3U3	Fascin	1.23	-1.23	-2.47
A0A8B6G0F8	Fibronectin type-III domain-containing protein	-0.72	0.72	1.44
A0A8B6EE15	Formyltetrahydrofolate dehydrogenase (EC 1.5.1.6)	1.97	-1.97	-3.94
A0A8S3UCU2	FrmA (EC 1.1.1.1. EC 1.1.1.284)	0.79	-0.79	-1.58
A0A8S3TR72	Fructose-bisphosphate aldolase (EC 4.1.2.13)	1.25	-1.25	-2.51
A0A8B6BVI8	Fructose-bisphosphate aldolase (EC 4.1.2.13)	1.29	-1.29	-2.58
A0A8S3Q6H6	Fumarylacetoacetase (EC 3.7.1.2) (Fumarylacetoacetate hydrolase)	1.62	-1.62	-3.25
A0A0C5Q4G0	Galectin	0.83	-0.83	-1.65
A0A8B6CKU6	Gasdermin pore forming domain-containing protein	0.62	-0.62	-1.23
A0A8B6D0L8	Gelsolin	1.03	-1.03	-2.05
A0A8S3QNI2	Glucose-6-phosphate isomerase (EC 5.3.1.9)	0.94	-0.94	-1.87
A0A8B6BHJ8	Glucosidase 2 subunit beta	0.39	-0.39	-0.78
A0A8B6DQP9	glutamate dehydrogenase [NAD(P)(+)] (EC 1.4.1.3)	0.87	-0.87	-1.74
A0A8S3Q5G9	Glutathione S-transferase omega (GSTO) (EC 1.20.4.2) (EC 1.8.5.1) (EC 2.5.1.18) (Glutathione-dependent dehydroascorbate reductase) (Monomethylarsonic acid reductase)	1.65	-1.65	-3.30
A0A6J8BZR7	Glyoxalase 1.Glyoxalase domain-containing protein 4	1.19	-1.19	-2.37
A0A8S3VGU6	GMP reductase (GMPR) (EC 1.7.1.7) (Guanosine 5'-monophosphate oxidoreductase) (Guanosine monophosphate reductase)	1.39	-1.39	-2.79
A0A6J8ENV8	GNB1	0.68	-0.68	-1.35
A0A8S3R8I4	Golgi reassembly-stacking protein 2.Golgi reassembly-stacking protein 1	0.38	-0.38	-0.77
A0A6J8F5H8	aspartate transaminase (EC 2.6.1.1)	0.66	-0.66	-1.32
A0A2H4WCW9	Guanine nucleotide-binding protein subunit alpha	1.43	-1.43	-2.86
A0A6J8CA81	GSK3B (EC 2.7.11.26)	0.99	-0.99	-1.98
A0A0L8I0K2	small monomeric GTPase (EC 3.6.5.2)	1.09	-1.09	-2.18
A0A8B6HQH7	Heat shock 70kDa protein 4	0.55	-0.55	-1.11

A0A411G5T5	Hemicentin-like protein	0.31	-0.31	-0.62
A0A8B6D225	Heterogeneous nuclear ribonucleoprotein A1/A3	0.62	-0.62	-1.24
A0A8B6ETA9	Histone H1/5	0.84	-0.84	-1.68
Q6WV85	Histone H2B	0.37	-0.37	-0.74
A0A8S3RBA2	HNRNPA1_3	1.74	-1.74	-3.48
A0A6J8ESL8	HNRNPABD	1.07	-1.07	-2.14
A0A8S3U136	hydroxyacid-oxoacid transhydrogenase (EC 1.1.99.24)	0.54	-0.54	-1.09
A0A8B6GQQ8	Ig-like domain-containing protein	1.60	-1.60	-3.19
A0A8B6DGI1	Insulin-like growth factor 2 mRNA-binding protein 1	0.59	-0.59	-1.17
A0A8B6CIE6	Isocitrate dehydrogenase [NAD] subunit. mitochondrial	0.71	-0.71	-1.42
A0A8B6ET27	Kazal-like domain-containing protein	1.29	-1.29	-2.58
A0A6J8A1Y0	KCTD8_12_16	0.70	-0.70	-1.40
A0A8B6FVU2	Kinesin-like protein	1.42	-1.42	-2.84
A0A8B6G9Q8	Laminin. alpha 3/5	0.43	-0.43	-0.86
A0A8B6CXT1	Laminin. beta 1	0.77	-0.77	-1.53
A0A8B6CGB4	Large subunit ribosomal protein L10Ae	1.35	-1.35	-2.69
A0A8B6F9E7	Large ribosomal subunit protein eL22 (60S ribosomal protein L22)	-0.52	0.52	1.04
A0A8B6BGD5	Large subunit ribosomal protein L37e	1.73	-1.73	-3.46
A0A8B6GGC2	Large subunit ribosomal protein L5e	0.83	-0.83	-1.67
A0A8B6E6I0	Large subunit ribosomal protein L7e	0.68	-0.68	-1.36
A0A8B6BTE6	Large ribosomal subunit protein P1 (60S acidic ribosomal protein P1)	0.63	-0.63	-1.25
A0A6J8B8C6	LHFPL	1.14	-1.14	-2.28
A0A8B6GHC2	Lipocalin/cytosolic fatty-acid binding domain-containing protein	2.17	-2.17	-4.34
A0A0K0YB29	LKD-rich protein-1	0.35	-0.35	-0.70
A0A8S3U3J8	LRP2	1.62	-1.62	-3.23
A0A8S3UA31	LRP2	1.54	-1.54	-3.08
A0A8B6GPG7	Uncharacterized protein	0.81	-0.81	-1.62
A0A8B6G660	Lupus La protein	1.28	-1.28	-2.55
A0A8B6DCW7	MAGUK p55 subfamily member 6	1.15	-1.15	-2.30
A0A6J8CXM9	Malectin-B.Malectin-A.Malectin	1.93	-1.93	-3.85
A0A8B6D8H6	Mammalian ependymin-related protein 1	0.69	-0.69	-1.37
A0A6J8EFL5	Mammalian ependymin-related protein 1	1.50	-1.50	-3.00
A0A8S3RY54	MAPRE	1.28	-1.28	-2.57
A0A8B6BF41	MARVEL domain-containing protein	1.62	-1.62	-3.23
A0A8B6CZ83	Choice-of-anchor I domain-containing protein	1.45	-1.45	-2.89
Q697L7	Metallothionein 10-III	2.04	-2.04	-4.09
A0A8S3QX71	methylmalonate-semialdehyde dehydrogenase (CoA acylating) (EC 1.2.1.27)	1.38	-1.38	-2.76
A0A6J8F1I0	MTCH	1.06	-1.06	-2.12
A0A8B6H0Z6	Myosin VI	-0.66	0.66	1.31
A0A8B6EU84	Na(+)/H(+) exchange regulatory cofactor NHx10-RF1	1.14	-1.14	-2.28
A0A8S3U4W4	NADP-dependent oxidoreductase domain-containing protein	0.53	-0.53	-1.06
A0A6J8BDN2	NADP-dependent oxidoreductase domain-containing protein	0.47	-0.47	-0.94
A0A6J8CRB1	NHP2	1.49	-1.49	-2.97
A0A8S3QGN5	NME8	0.64	-0.64	-1.27
A0A8S3PMK0	Nuclear migration protein nudC (Nuclear distribution protein C homolog)	0.94	-0.94	-1.89
A0A8B6C4N8	protein-disulfide reductase (EC 1.8.1.8)	0.43	-0.43	-0.85
A0A8S3ULS9	Nucleoside diphosphate kinase (EC 2.7.4.6)	1.12	-1.12	-2.24
A0A8S3UGN8	Obg-like ATPase 1	0.42	-0.42	-0.83
A0A8B6EVV0	OTU domain-containing protein	-0.29	0.29	0.59
A0A8B6BMW1	Pancreatic elastase II (EC 3.4.21.71)	1.46	-1.46	-2.92
A0A6J8DW13	PDIA5 (EC 5.3.4.1)	0.65	-0.65	-1.29

A0A8S3TBS5	PDZ domain-containing protein	1.34	-1.34	-2.68
A0A8B6DFV3	Peptidase M24 domain-containing protein	1.52	-1.52	-3.03
A0A8B6D8S4	Peptidase metallopeptidase domain-containing protein	0.36	-0.36	-0.72
A0A8B6BIH5	Peptidyl-prolyl cis-trans isomerase (PPIase) (EC 5.2.1.8)	0.89	-0.89	-1.78
A0A161I1V8	Peptidyl-prolyl cis-trans isomerase (PPIase) (EC 5.2.1.8)	0.35	-0.35	-0.69
A0A6J8DKH3	Peptidyl-prolyl cis-trans isomerase (PPIase) (EC 5.2.1.8)	0.92	-0.92	-1.83
A0A8S3SEX5	Peptidyl-prolyl cis-trans isomerase (PPIase) (EC 5.2.1.8)	1.89	-1.89	-3.78
A0A8B6EBJ6	peptidylprolyl isomerase (EC 5.2.1.8)	1.51	-1.51	-3.01
A0A8S3RID5	peptidylprolyl isomerase (EC 5.2.1.8)	0.65	-0.65	-1.30
A0A8B6D5H2	Uncharacterized protein	0.40	-0.40	-0.80
A0A8B6GKK6	Peroxiredoxin-5 (EC 1.11.1.24)	1.11	-1.11	-2.22
A0A8S3R7F6	phosphoglycerate mutase (2.3-diphosphoglycerate-dependent) (EC 5.4.2.11)	0.43	-0.43	-0.86
A0A8S3S8I1	phosphoglucomutase (alpha-D-glucose-1.6-bisphosphate-dependent) (EC 5.4.2.2)	0.32	-0.32	-0.64
A0A6J7ZU18	Phosphoglycerate kinase (EC 2.7.2.3)	0.87	-0.87	-1.75
A0A6J8AQ45	Phospholipid scramblase	0.68	-0.68	-1.36
A0A6J8EPJ2	Phospholipid scramblase	1.42	-1.42	-2.84
A0A8B6DQL6	Enolase (EC 4.2.1.11) (2-phospho-D-glycerate hydro-lyase) (2-phosphoglycerate dehydratase)	0.80	-0.80	-1.60
A0A8S3SL70	PMPCB (EC 3.4.24.64)	0.42	-0.42	-0.84
A0A6J8E914	PRKAR	1.08	-1.08	-2.15
A0A8B6FT25	Profilin	1.28	-1.28	-2.57
A0A8B6CHE6	Profilin	1.73	-1.73	-3.46
A0A8B6D5R1	Programmed cell death 6-interacting protein	-0.41	0.41	0.82
A0A8B6G5S6	Prohibitin	2.03	-2.03	-4.06
A0A8B6CYK1	Proteasome subunit alpha type	1.66	-1.66	-3.33
A0A8S3UDK6	Proteasome subunit alpha type	1.09	-1.09	-2.18
A0A8B6DC08	Protein DEK	1.02	-1.02	-2.04
A0A8B6FXU3	Protein disulfide-isomerase A6 (EC 5.3.4.1)	0.66	-0.66	-1.32
A0A6J8DCE2	Protein RCC2 homolog.Protein RCC2	1.30	-1.30	-2.60
A0A8B6EIQ6	Protein sleepless	2.52	-2.52	-5.04
A0A8S3S9K8	Uncharacterized protein	1.33	-1.33	-2.65
A0A8B6CNR6	Protocadherin Fat 4	-0.88	0.88	1.76
A0A8S3UA94	PSAP	-0.77	0.77	1.54
A0A0G2YLG5	PTPRF (EC 3.1.3.48) (Shell protein-6)	0.76	-0.76	-1.52
A0A6J8BU32	PURA	0.31	-0.31	-0.61
A0A8B6BR62	Pyruvate dehydrogenase E1 component subunit alpha (EC 1.2.4.1)	1.47	-1.47	-2.95
A0A8S3QRM1	QDPR (EC 1.5.1.34)	0.60	-0.60	-1.19
A0A8S3RSD5	Queuosine 5'-phosphate N-glycosylase/hydrolase (EC 3.2.2.-) (Queuosine-nucleotide N-glycosylase/hydrolase)	-0.40	0.40	0.81
A0A6J8CI59	RAB1A	1.39	-1.39	-2.79
A0A0B7AP29	Ras-related protein Rab-2A	0.32	-0.32	-0.64
A0A8B6FT60	Radial spoke head protein 4A	0.75	-0.75	-1.50
A0A8B6GLW0	Radial spoke head protein 9 homolog	0.40	-0.40	-0.81
A0A2C9JE50	Ras-related protein Rab-11B-like	1.72	-1.72	-3.44
A0A6J8CUS4	Ras-related protein Rab^{-14} (EC 3.6.5.2)	0.66	-0.66	-1.33
A0A8B6G941	Ras-related protein Rab-35	0.87	-0.87	-1.73
A0A6J8F240	RBM4	1.43	-1.43	-2.86
A0A8B6C295	Receptor expression-enhancing protein	1.44	-1.44	-2.89
A0A6J8DY17	RIB43A-like with coiled-coils protein 2	1.16	-1.16	-2.32
A0A077H3K4	Ribosomal protein L23a	1.78	-1.78	-3.56
A0A077H3J9	Small ribosomal subunit protein uS9 (40S ribosomal protein S16)	1.55	-1.55	-3.10

A0A8S3QYE0	Uncharacterized protein	0.84	-0.84	-1.68
A0A8B6FIE8	RNA-binding protein 4	1.36	-1.36	-2.73
A0A6J8A0F1	Large ribosomal subunit protein eL24 (60S ribosomal protein L24)	0.44	-0.44	-0.88
A0A8S3UK82	RP-L26e	1.98	-1.98	-3.97
A0A8B6EXY4	RRM domain-containing protein	0.92	-0.92	-1.83
A0A8B6HNM7	S-(Hydroxymethyl)glutathione dehydrogenase / alcohol dehydrogenase (EC 1.1.1.284)	1.10	-1.10	-2.19
A0A8B6CEB0	Saposin	0.55	-0.55	-1.11
A0A6J8DVF9	SEC61A	0.45	-0.45	-0.91
A0A8B6G4G0	Selenoprotein F/M domain-containing protein	2.15	-2.15	-4.30
A0A8S3RKC5	Serine hydroxymethyltransferase (EC 2.1.2.1)	1.00	-1.00	-2.00
A0A8B6H826	SH3 domain-containing protein	0.69	-0.69	-1.38
A0A8B6GPC3	SHSP domain-containing protein	1.06	-1.06	-2.13
A0A8S3RCR9	SLC25A23S	0.59	-0.59	-1.17
A0A8B6F648	Small nuclear ribonucleoprotein Sm D2 (Sm-D2) (snRNP core protein D2)	2.22	-2.22	-4.44
A0A8B6CSI5	Small subunit ribosomal protein S17e	2.16	-2.16	-4.33
A0A8B6HL62	Small subunit ribosomal protein S19e	-0.42	0.42	0.83
A0A6J8BWT3	SNX1_2	1.24	-1.24	-2.49
A0A8S3VFI8	SPATA6	0.93	-0.93	-1.86
A0A6J8D7K5	SQOR (EC 1.8.5.8)	1.01	-1.01	-2.02
A0A6J8E460	Succinate--CoA ligase [ADP-forming] subunit beta. mitochondrial (EC 6.2.1.5) (Succinyl-CoA synthetase beta chain) (SCS-beta)	1.09	-1.09	-2.18
A0A8S3R089	Succinate--CoA ligase [ADP/GDP-forming] subunit alpha. mitochondrial (EC 6.2.1.4) (EC 6.2.1.5) (Succinyl-CoA synthetase subunit alpha) (SCS-alpha)	0.90	-0.90	-1.80
A0A8B6CGG6	Suppressor of G2 allele of SKP1	0.66	-0.66	-1.31
A0A8B6EYT5	Synaptotagmin-1	0.84	-0.84	-1.67
A0A8B6DIG2	T-complex protein 1 subunit alpha (CCT-alpha)	0.42	-0.42	-0.84
A0A8B6E6D8	T-complex protein 1 subunit gamma	2.11	-2.11	-4.21
A0A8B6F9J1	T-complex protein 1 subunit zeta	0.65	-0.65	-1.30
A0A6J8BSI9	Tektin	1.34	-1.34	-2.67
A0A6J8BBL7	Tektin	1.02	-1.02	-2.04
A0A8S3SXV7	Tetraspanin	0.83	-0.83	-1.66
A0A8B6CJW7	Tetraspanin	1.54	-1.54	-3.09
A0A8B6DN29	Tetraspanin	1.29	-1.29	-2.59
A0A6J8AMT5	Tetratricopeptide repeat protein 29	0.67	-0.67	-1.35
A0A8B6H5W0	Thioredoxin	1.97	-1.97	-3.95
A0A8S3QJ24	Thioredoxin-like protein 1	0.57	-0.57	-1.15
A0A8B6FN66	Thyroglobulin type-1 domain-containing protein	1.77	-1.77	-3.55
A0A8B6C4T2	Thyroglobulin type-1 domain-containing protein	1.43	-1.43	-2.87
A0A8S3R738	TIMP4	0.81	-0.81	-1.61
A0A8S3R9Y2	TIMP4	1.59	-1.59	-3.17
A0A8B6FQW6	Transaldolase (EC 2.2.1.2)	1.22	-1.22	-2.44
A0A8B6FLR4	Transcription elongation factor	0.68	-0.68	-1.36
A0A8B6CWL2	Transgelin	1.54	-1.54	-3.08
A0A6J7ZVG6	Transgelin	1.05	-1.05	-2.11
A0A8B6H8C4	Tripeptidyl-peptidase 2 (EC 3.4.14.10) (Tripeptidyl aminopeptidase)	-0.84	0.84	1.67
A0A8B6F7C4	Tropomyosin	0.80	-0.80	-1.59
A0A6J8DNP0	TROVE2	0.58	-0.58	-1.16
A0A0K2D7M8	TSP_1 domain containing protein-1	1.12	-1.12	-2.24
A0A0B6Z2B0	Tubulin beta chain	0.47	-0.47	-0.94
A0A8B6DWZ2	Tumor necrosis factor ligand superfamily member 10	1.20	-1.20	-2.39

A0A8S3TTF4	Tumor protein D54	1.77	-1.77	-3.53
A0A8B6FHC2	Tyrosinase (EC 1.14.18.1)	1.31	-1.31	-2.62
A0A8B6FCM7	Tyrosine-protein kinase ephrin type A/B receptor-like domain-containing protein	0.97	-0.97	-1.95
A0A8S3SPC1	UBA domain-containing protein	-0.53	0.53	1.05
A0A8S3QZQ4	UBE2N (EC 2.3.2.23)	2.09	-2.09	-4.18
A0A8B6CGQ6	Ubiquinol-cytochrome c reductase iron-sulfur subunit (EC 1.10.2.2)	1.10	-1.10	-2.19
A0A0B6Y8N5	Ubiquitin-ribosomal protein eL40 fusion protein	1.92	-1.92	-3.84
A0A6J8CSZ8	Uncharacterized oxidoreductase C513.07	1.58	-1.58	-3.15
A0A8B6FG82	Uncharacterized protein	-0.70	0.70	1.39
A0A3L5TR65	Uncharacterized protein	1.28	-1.28	-2.55
A0A8B6FNN6	Uncharacterized protein	1.67	-1.67	-3.35
A0A8B6G8E3	SCO-spondin	0.91	-0.91	-1.82
A0A8B6GHG6	Uncharacterized protein	2.00	-2.00	-4.01
A0A8B6CU91	Uncharacterized protein	1.47	-1.47	-2.95
A0A8B6CEC3	Metallothionein	0.47	-0.47	-0.95
A0A8B6HNQ5	Uncharacterized protein	1.23	-1.23	-2.45
A0A8B6FSV1	Uncharacterized protein	0.57	-0.57	-1.13
A0A8B6CTG8	Uncharacterized protein	1.69	-1.69	-3.38
A0A8B6ENM5	Uncharacterized protein	1.61	-1.61	-3.21
A0A8B6CCA0	Uncharacterized protein	1.45	-1.45	-2.90
A0A8B6DTT6	Uncharacterized protein	1.49	-1.49	-2.98
A0A6J8CD73	Uncharacterized protein	0.81	-0.81	-1.61
A0A8S3T9B8	Uncharacterized protein	1.55	-1.55	-3.10
A0A8S3RZM2	Kyphoscoliosis peptidase	2.43	-2.43	-4.86
A0A8B6F5H6	Uncharacterized protein	1.85	-1.85	-3.70
A0A8B6H837	Uncharacterized protein	0.99	-0.99	-1.97
A0A8B6FQ26	Uncharacterized protein	2.14	-2.14	-4.28
A0A8B6DR40	EF-hand domain-containing protein 1	0.55	-0.55	-1.10
A0A8B6BSD4	Counting factor associated protein D	-1.26	1.26	2.52
A0A8B6CRI3	Uncharacterized protein	0.85	-0.85	-1.70
A0A8B6CM77	Uncharacterized protein	0.33	-0.33	-0.65
A0A8B6DHU3	Uncharacterized protein	0.45	-0.45	-0.90
A0A8B6CG35	Uncharacterized protein	0.46	-0.46	-0.92
A0A8S3RSI4	Uncharacterized protein	2.29	-2.29	-4.58
A0A8B6DI09	Uncharacterized protein	1.45	-1.45	-2.89
A0A8B6CVX7	Uncharacterized protein	1.03	-1.03	-2.06
A0A8B6HFY8	Uncharacterized protein	2.13	-2.13	-4.26
A0A8B6F299	Mucin-2-like	0.44	-0.44	-0.89
A0A8B6EZW5	Uncharacterized protein	1.64	-1.64	-3.28
A0A8B6C5E2	Uncharacterized protein	2.59	-2.59	-5.18
A0A6J8CCF5	NodB homology domain-containing protein	-0.30	0.30	0.61
A0A8S3U5X7	Uncharacterized protein	0.81	-0.81	-1.62
A0A8B6C9P4	Uncharacterized protein	0.80	-0.80	-1.61
A0A8B6H7C0	Uncharacterized protein	1.07	-1.07	-2.13
A0A8B6G6F1	VWFD domain-containing protein	-7.11	7.11	14.20
A0A8B6FPE6	Uncharacterized protein	1.66	-1.66	-3.33
A0A8S3S991	Uncharacterized protein	2.28	-2.28	-4.57
A0A8B6BE54	RIIa domain-containing protein	0.47	-0.47	-0.95
A0A8B6GK87	Uncharacterized protein	-1.11	1.11	2.22
A0A8S3SVU1	Sperm microtubule inner protein 1 C-terminal domain-containing protein	1.44	-1.44	-2.88
A0A8B6D1E0	Uncharacterized protein	1.25	-1.25	-2.50

A0A8B6CXZ8	Ciliary microtubule inner protein 2C	1.25	-1.25	-2.50
A0A8B6HI74	Uncharacterized protein	0.93	-0.93	-1.86
A0A6J8EM21	Phosphatidylinositol transfer protein N-terminal domain-containing protein	0.83	-0.83	-1.66
A0A8S3UJ52	Uncharacterized protein	1.25	-1.25	-2.49
A0A8B6D1P6	Uncharacterized protein	1.61	-1.61	-3.23
A0A8B6EZY7	CTCK domain-containing protein	1.45	-1.45	-2.91
A0A8S3QP16	Uncharacterized protein	0.47	-0.47	-0.93
A0A8S3UR85	Uncharacterized protein	1.11	-1.11	-2.23
A0A8S3SPV4	Uncharacterized protein	1.20	-1.20	-2.40
A0A8S3TKR9	Uncharacterized protein	1.04	-1.04	-2.07
A0A8B6FV73	Uncharacterized protein	1.19	-1.19	-2.38
A0A8S3S0N2	Uncharacterized protein	-0.62	0.62	1.24
A0A8S3QG66	Uncharacterized protein	1.29	-1.29	-2.59
A0A8B6GYD1	Uncharacterized protein	0.80	-0.80	-1.60
A0A8S3U726	Uncharacterized protein	0.46	-0.46	-0.92
A0A8S3QIJ9	UspA domain-containing protein	0.64	-0.64	-1.27
A0A8B6G9T0	UspA domain-containing protein	0.77	-0.77	-1.54
A0A8B6ESB0	UTP--glucose-1-phosphate uridylyltransferase (EC 2.7.7.9)	0.97	-0.97	-1.93
A0A8S3QEM3	Vacuolar proton pump subunit B (V-ATPase subunit B) (Vacuolar proton pump subunit B)	0.57	-0.57	-1.15
A0A6J8DNW3	VDAC2	1.11	-1.11	-2.22
A0A8B6E1P9	Vitelline membrane outer layer 1-like protein	2.29	-2.29	-4.58
A0A8B6DLG6	Vitellogenic carboxypeptidase-like protein (EC 3.4.16.-)	0.72	-0.72	-1.44
A0A8B6C9L4	VWFA domain-containing protein	0.58	-0.58	-1.17
A0A8B6HDB4	Actin-interacting protein 1	1.11	-1.11	-2.21
A0A8B6F1U6	Cilia- and flagella-associated protein 52	1.22	-1.22	-2.43
A0A8B6CY00	2-oxoglutarate dehydrogenase E1 component (EC 1.2.4.2)	0.66	-0.66	-1.31
A0A210Q521	26S protease regulatory subunit 6B	0.82	-0.82	-1.64
A0A8B6GS06	26S proteasome regulatory subunit N2	1.17	-1.17	-2.34
A0A8B6GHX0	26S proteasome regulatory subunit N3	0.62	-0.62	-1.23
A0A8S3VGU8	4-hydroxyphenylpyruvate dioxygenase	1.20	-1.20	-2.39
A0A8S3QVD1	Small ribosomal subunit protein uS17 (40S ribosomal protein S11)	1.09	-1.09	-2.17
A0A6J8EWE8	40S ribosomal protein S12	0.84	-0.84	-1.69
A0A2C9JRX0	Small ribosomal subunit protein uS19 (40S ribosomal protein S15)	1.11	-1.11	-2.22
A0A8B6G0N3	Small ribosomal subunit protein uS13 (40S ribosomal protein S18)	1.13	-1.13	-2.26
V4AS57	40S ribosomal protein S25	1.67	-1.67	-3.34
A0A8B6EK07	40S ribosomal protein S26	0.56	-0.56	-1.13
A0A8B6CVW2	40S ribosomal protein S27	1.29	-1.29	-2.57
A0A210PU23	40S ribosomal protein S5	1.13	-1.13	-2.27
A0A3G1CJL1	40S ribosomal protein S8	1.20	-1.20	-2.41
A0A8B6BQ82	5'-AMP-activated protein kinase subunit beta-1	1.64	-1.64	-3.28
A0A8S3RZZ6	60S acidic ribosomal protein P0	1.64	-1.64	-3.29
B6ZCB1	Large ribosomal subunit protein P2 (60S acidic ribosomal protein P2)	0.31	-0.31	-0.62
A0A8S3V934	60S ribosomal protein L18a	0.83	-0.83	-1.67
A0A077GYU2	Large ribosomal subunit protein uL14 (60S ribosomal protein L23)	1.22	-1.22	-2.44
A0A077H3N0	60S ribosomal protein L27	1.07	-1.07	-2.15
A0A077GZK7	Large ribosomal subunit protein eL28 (60S ribosomal protein L28)	1.28	-1.28	-2.55
A0A8B6G8D8	Large ribosomal subunit protein eL31 (60S ribosomal protein L31)	2.26	-2.26	-4.51
A0A8B6DEA0	Large ribosomal subunit protein eL34 (60S ribosomal protein L34)	2.18	-2.18	-4.36
A0A8S3R313	Large ribosomal subunit protein eL36 (60S ribosomal protein L36)	0.58	-0.58	-1.16
A0A8B6EI40	Large ribosomal subunit protein eL6 (60S ribosomal protein L6)	0.55	-0.55	-1.09

A0A6J8A5K2	Large ribosomal subunit protein uL2 (60S ribosomal protein L8)	1.59	-1.59	-3.18
A0A8B6FK68	Xylose isomerase (EC 5.3.1.5)	1.37	-1.37	-2.73
A0A8S3UF93	YWHAE	1.02	-1.02	-2.04

Table S1. List of differentially expressed proteins (DEPs) in *Mytilus galloprovincialis* plantigrades exposed to 2′-deoxyinosine (**2′**). DEPs were obtained from pairwise comparisons between the control group (0.1% DMSO) and exposed group at 12.5 μM. using a threshold of lfc = $\log_2(1.5)$. Represented are the relative expression of DEPs on the control group (Ctr-) and the exposed group (12.5 μM). and the obtained fold change of each DEP.

**Reference Sequence**	**Protein Name**	**Relative Expression**		**Fold Change**
		**Ctr-**	**12.5 μM**	
A0A8B6DSL8	Adenosylhomocysteinase (EC 3.13.2.1)	0.70	-0.70	-1.40
A0A8B6GV47	ADP/ATP translocase (ADP.ATP carrier protein)	-0.63	0.63	1.26
A0A8B6GU32	ATP synthase subunit gamma	0.98	-0.98	-1.96
A0A8S3Q2E4	ATPeF1D	-1.44	1.44	2.89
A0A6J8EBJ6	Band 7 domain-containing protein	0.33	-0.33	-0.66
A0A8B6GW86	C1q domain-containing protein	1.34	-1.34	-2.68
A0A8B6G4E0	Calcium-transporting ATPase (EC 7.2.2.10)	-0.61	0.61	1.23
A0A8B6FIN1	Chitin-binding type-2 domain-containing protein	-1.02	1.02	2.03
A0A8B6C153	Citrate synthase	-0.54	0.54	1.09
A0A8B6ENY2	Cellular nucleic acid-binding protein	-0.30	0.30	0.59
A0A8S3R8F7	E2.7.3.3 (EC 2.7.3.3)	0.59	-0.59	-1.18
D9IWS1	Elongation factor 1-alpha	0.71	-0.71	-1.42
A0A8B6EYD8	Elongation factor 2	-0.58	0.58	1.16
A0A8S3QB23	H3	-0.37	0.37	0.74
A0A8B6DBF4	Metalloendopeptidase (EC 3.4.24.-)	1.04	-1.04	-2.09
A0A8B6G1S8	Protein disulfide-isomerase (EC 5.3.4.1)	0.31	-0.31	-0.61
A0A8S3Q9N6	SHSP domain-containing protein	0.82	-0.82	-1.64
A0A8B6D810	Spectrin alpha	-0.41	0.41	0.81
Q966V3	Transgelin	-0.77	0.77	1.54
A0A076FIR9	Tubulin beta chain	0.52	-0.52	-1.03
A0A8B6CEW4	Uncharacterized protein	-0.80	0.80	1.60
A0A8B6DC97	MAM domain-containing protein	-0.88	0.88	1.75
A0A8B6BV40	VWFA domain-containing protein	-1.25	1.25	2.51
A0A8B6FD83	Collagen. type II. alpha	-0.49	0.49	0.98
A0A8B6G747	Collagen. type V/XI/XXIV/XXVII. alpha	-0.60	0.60	1.19
A0A8S3QII9	CTSL (EC 3.4.22.15)	0.51	-0.51	-1.02
A0A8B6HEV7	Cysteine and glycine-rich protein	-0.52	0.52	1.03
A0A8B6F031	Cytochrome b-c1 complex subunit 6	1.12	-1.12	-2.25
A0A8B6D685	Dihydrolipoyllysine-residue succinyltransferase component of 2-oxoglutarate dehydrogenase complex. mitochondrial (EC 2.3.1.61) (2-oxoglutarate dehydrogenase complex component E2) (E2K)	0.96	-0.96	-1.92
A0A409V740	Dna-binding a protein	-1.35	1.35	2.69
A0A3L5TQZ0	Fc-e cd23 receptor	0.66	-0.66	-1.32
A0A6J8ECB1	Glucose-6-phosphate isomerase (EC 5.3.1.9)	0.41	-0.41	-0.82
A0A8B6DQP9	glutamate dehydrogenase [NAD(P)(+)] (EC 1.4.1.3)	0.96	-0.96	-1.93
A0A8B6H497	Granulins domain-containing protein	-0.38	0.38	0.76

A0A411G5T5	Hemicentin-like protein	1.43	-1.43	-2.86
K1Q324	Heterogeneous nuclear ribonucleoprotein K	0.30	-0.30	-0.60
A0A0B7AUT8	Histone H2A	1.08	-1.08	-2.16
A0A8B6DFJ0	Insulin-like growth factor 2 mRNA-binding protein 1	-0.59	0.59	1.19
A0A8B6BTE6	Large ribosomal subunit protein P1 (60S acidic ribosomal protein P1)	0.38	-0.38	-0.76
A0A8S3Q6R0	LMNB	-0.37	0.37	0.75
A0A8B6C2Y2	Malate dehydrogenase (EC 1.1.1.37)	-0.78	0.78	1.56
A0A8B6FW38	Molecular chaperone DnaK	0.30	-0.30	-0.60
A0A6J8APW2	NID	0.42	-0.42	-0.84
A0A8S3ULS9	Nucleoside diphosphate kinase (EC 2.7.4.6)	0.88	-0.88	-1.76
A0A8B6D9K2	P-type domain-containing protein	0.96	-0.96	-1.91
A0A6J8DKH3	Peptidyl-prolyl cis-trans isomerase (PPIase) (EC 5.2.1.8)	0.84	-0.84	-1.67
A0A8S3TQI9	Sarcoplasmic calcium-binding protein	-1.49	1.49	2.97
A0A6J8EGE4	TRAF4	-0.57	0.57	1.14
A0A8S3QSQ2	Ubiquitin-ribosomal protein eL40 fusion protein	-0.32	0.32	0.64
A0A3L5TR65	Uncharacterized protein	-0.92	0.92	1.84
A0A8B6E104	Uncharacterized protein	-0.92	0.92	1.84
A0A8B6GUE8	WAP domain-containing protein	1.22	-1.22	-2.44
A0A8B6CB58	Uncharacterized protein	0.84	-0.84	-1.68
A0A8B6DFD7	Uncharacterized protein	-0.51	0.51	1.02
A0A8B6BNI7	Uncharacterized protein	0.98	-0.98	-1.96
A0A8B6DPQ0	SCO-spondin	0.51	-0.51	-1.01
A0A8B6FV73	Uncharacterized protein	0.48	-0.48	-0.97
A0A8B6CCQ9	Uncharacterized protein	-0.37	0.37	0.74
A0A8B6H3D0	Vesicle-associated membrane protein-associated protein A	-0.97	0.97	1.94
A0A8B6GXU1	Vitelline membrane outer layer 1-like protein	1.63	-1.63	-3.25
A0A8B6EK07	40S ribosomal protein S26	0.30	-0.30	-0.61
A0A3G1CJL1	40S ribosomal protein S8	0.78	-0.78	-1.56
A0A8S3QVR3	Small ribosomal subunit protein uS2	-1.00	1.00	2.00
A0A6J8B1B1	6-phosphofructokinase (EC 2.7.1.11) (Phosphohexokinase)	0.78	-0.78	-1.56
A0A8S3RZZ6	60S acidic ribosomal protein P0	-0.90	0.90	1.81
A0A6J8DYJ1	Large ribosomal subunit protein uL11 (60S ribosomal protein L12)	0.38	-0.38	-0.76
A0A8S3V934	60S ribosomal protein L18a	0.66	-0.66	-1.32
A0A077H0N7	60S ribosomal protein L7a	-0.59	0.59	1.19

Table S2. Results of BLASTp analysis for DEPs identified in response to hypoxanthine arabinoside (**1′**). The table lists homologous proteins from *Magallana gigas*. along with their corresponding accession numbers retrieved from the UniProt and Ensembl Metazoa databases.

	UniProt			Ensembl	
**Reference Sequence**	**Accession number**	**Protein name**	**e-Value**	**Accession number**	**e-Value**
A0A8B6C5G7	A0A8W8N8B2	3-ketoacyl-CoA thiolase. mitochondrial	0.00	G4782.3	0.00
A0A8B6F498	A0A8W8KG37	Acyl-coenzyme A oxidase	$7.45x10^{-75}$	G23748.2	$5.82x10^{-75}$
A0A8B6DSL8	K1RW85	Adenosylhomocysteinase	0.00	G22300.4	0.00
A0A8S3SJS5	A0A8W8NE65	Adenylyl cyclase-associated protein	$1.88x10^{-178}$	G5795.12	$1.47x10^{-178}$
A0A8S3S1H7	A0A8W8KME6	Adenylate kinase isoenzyme 5	0.00	G24232.1	0.00
A0A8S3TZ88	K1PRS8	Adenylate kinase 8	0.00	G25200.4	0.00
A0A8B6D0Q7	A0A8W8LYD4	ADP-ribosylation factor	$6.42x10^{-98}$	G29686.9	$5.02x10^{-98}$

A0A8B6C281	K1RGK4	Villin-1	0.00	G27734.1	$1.32x10^{-114}$
A0A8B6EIW3	K1RGF4	alanine transaminase	0.00	G30434.4	0.00
A0A8B6BGG4	A0A8W8LL21	Saccharopine dehydrogenase (NAD(+). L-glutamate-forming)	0.00	G28495.1	0.00
A0A8B6D196	A0A8W8IJS1	Aminopeptidase	0.00	G14427.1	0.00
A0A8S3QGC9	K1RTF1	Annexin	0.00	G26210.8	0.00
A0A8S3VH93	A0A8W8I0T9	Cysteine-rich motor neuron 1 protein	$1.72x10^{-10}$	G11972.1	$1.34x10^{-10}$
A0A8S3QFL2	K1QQ16	AP complex subunit beta	0.00	G35149.7	0.00
A0A8S3S184	A0A8W8LLL1	Apextrin C-terminal domain-containing protein	$2.78x10^{-85}$	G28650.5	$2.17x10^{-85}$
A0A8B6BZ74	A0A8W8N9W6	Apoptosis inhibitor 5	0.00	G4455.1	0.00
A0A8B6D0T5	A0A8W8N549	Apple domain-containing protein	0.00	G432.1	0.00
A0A8S3TG22	A0A8W8JWW8	Aquaporin-4	$1.34x10^{-143}$	G20854.9	$1.05x10^{-143}$
A0A8S3VBZ6	A0A8W8LUT3	Rho GDP-dissociation inhibitor 1	$2.68x10^{-76}$	G297.6	$2.09x10^{-76}$
A0A8B6H8N6	A0A8W8IFX6	Asparagine--tRNA ligase. cytoplasmic	0.00	G13717.6	0.00
A0A8S3SXM2	A0A8W8NW87	ATP-dependent 6-phosphofructokinase	0.00	G7024.1	0.00
A0A8B6GBE6	A0A8W8L2N3	ATP synthase-coupling factor 6. mitochondrial	$1.28x10^{-05}$	G26087.10	$1.00x10^{-05}$
A0A8B6H7T7	A0A8W8MB85	ATP synthase subunit beta	0.00	G31998.34	0.00
A0A077GYU5	A0A8W8MGJ9	ATP synthase subunit d. mitochondrial	$3.25x10^{-12}$	G3298.5	$2.54x10^{-12}$
A0A8B6GU32	A0A8W8LM86	ATP synthase subunit gamma. mitochondrial	$4.16x10^{-124}$	G28739.12	$3.25x10^{-124}$
A0A6J8F3Y7	K1R9E2	EF-hand calcium-binding domain-containing protein 6	0.00	G16976.1	0.00
A0A6J8C7Z5	A0A8W8NDX9	ATP synthase subunit f. mitochondrial	$1.49x10^{-13}$	G650.2	$1.17x10^{-13}$
A0A8S3Q2E4	K1PQJ9	ATP synthase subunit delta. mitochondrial	$2.28x10^{-45}$	G269.2	$1.78x10^{-45}$
A0A6J8DY85	K1PIH2	V-type proton ATPase subunit E	$3.90x10^{-114}$	G19302.3	$3.05x10^{-114}$
A0A8B6BPI1	K1REC7	Tripartite motif-containing protein 56	$3.30x10^{-25}$	G23372.1	$4.95x10^{-25}$
A0A8B6F754	K1PKN2	Papilin	$1.75x10^{-49}$	G11186.1	$9.91x10^{-49}$
A0A8B6DQ42	A0A8W8HZ39	C-type lectin domain-containing protein	$4.20x10^{-23}$	G10490.1	$3.28x10^{-23}$
A0A8B6GW86	A0A8W8J8Q2	C1q domain-containing protein	$1.57x10^{-20}$	G17851.1	$1.23x10^{-20}$
A0A6J8E3N6	A0A8W8M3J3	C1q domain-containing protein	$5.55x10^{-06}$	G31159.1	$4.34x10^{-06}$
A0A8B6HA48	A0A8W8K425	C2 domain-containing protein	0.00	G22340.8	0.00
A0A8S3SGA7	A0A8W8LX26	Cadherin domain-containing protein	0.00	G2999.15	0.00
A0A8B6FBT9	K1Q3D9	Calmodulin	$1.89x10^{-23}$	G32596.6	$3.67x10^{-23}$
A0A8S3R7D4	A0A8W8KPL5	Calreticulin	0.00	G2451.27	0.00
A0A8B6DWN7	A0A8W8MDX4	Cyclic nucleotide-binding domain-containing protein	0.00	G3254.14	0.00
A0A8B6D440	A0A8W8NL52	Peptidase A1 domain-containing protein	0.00	G5738.16	0.00
A0A8B6GVE1	A0A8W8ME56	Ig-like domain-containing protein	$2.35x10^{-19}$	G32348.2	$1.84x10^{-19}$
A0A8S3URK5	A0A8W8N0M4	Chromo domain-containing protein	$8.72x10^{-21}$	G35434.7	$6.81x10^{-21}$
A0A8B6FIN1	K1QY92	Chitin-binding type-2 domain-containing protein	$5.43x10^{-49}$	G22619.1	$4.24x10^{-49}$
A0A8B6DBL3	A0A8W8KZ55	Chitin-binding type-2 domain-containing protein	$1.18x10^{-48}$	G25920.6	9.25x10-49
A0A8S3T4F4	A0A8W8L7P8	Choline transporter-like protein	0.00	G2630.12	0.00
A0A8B6F3H7	K1RQ81	Uncharacterized protein C15orf26-like protein	$8.94x10^{-155}$	G11651.1	$6.98x10^{-155}$
A0A8B6GX94	A0A8W8KCV2	Clathrin heavy chain	0.00	G23360.1	0.00
A0A6J8ESY4	A0A8W8N791	Small ribosomal subunit protein uS15	$6.61x10^{-106}$	G4635.1	$5.16x10^{-106}$
A0A6J8EHM6	A0A8W8HYQ8	Small ribosomal subunit protein uS5	$1.77x10^{-160}$	G1172.7	$1.39x10^{-160}$

https://doi.org/10.3390/xxxxx

A0A8B6G7J5	A0A8W8J403	Large ribosomal subunit protein uL13	4.84×10^{-119}	G16773.14	3.78×10^{-119}
A0A077H0N7	A0A8W8L109	60S ribosomal protein L7a	1.38×10^{-155}	G25420.3	1.08×10^{-155}
Q9Y0D6	Q8TA69	Actin 2	0.00	G27522.32	0.00
A0A8S3Q5J1	K1RH58	Alpha-actinin. sarcomeric	0.00	G13016.1	2.24×10^{-112}
A0A8B6GV47	K1R0Y9	ADP/ATP translocase	0.00	G730.11	0.00
A0A8B6D2T4	A0A8W8MRV1	ADP-ribosylation factor	5.31×10^{-124}	G34817.29	4.15×10^{-124}
A0A8B6GKE0	K1QNT7	Aldedh domain-containing protein	0.00	G12763.28	0.00
A0A8S3T4E0	K1QNT7	Aldedh domain-containing protein	0.00	G12763.28	0.00
A0A8B6CW94	A0A8W8I5S2	Alpha-1.4 glucan phosphorylase	0.00	G12639.2	0.00
A0A8B6DXX6	A0A8W8LM76	Apextrin C-terminal domain-containing protein	1.68×10^{-95}	G28654.4	1.31×10^{-95}
A0A8B6HT02	A0A8W8L239	Armadillo repeat-containing protein 4	0.00	G26323.3	0.00
A0A8B6CRC1	K1R6Z7	ATP synthase subunit alpha	0.00	G11926.23	0.00
A0A6J8EBJ6	A0A8W8L8X3	Flotillin-1	0.00	G26928.23	0.00
A0A8B6GBK5	A0A8W8I7B5	C-1-tetrahydrofolate synthase. cytoplasmic	0.00	G12935.2	0.00
A0A8B6FVI3	A0A8W8JHD6	C1q domain-containing protein	6.20×10^{-27}	G18610.6	4.84×10^{-27}
A0A8B6G4E0	A0A8W8KCX1	Calcium-transporting ATPase	0.00	G23365.9	0.00
A0A8B6E763	K1PMY9	Calmodulin	0.00	G11730.2	2.12×10^{-126}
A0A8S3QR75	A0A8W8HQW9	Calpain catalytic domain-containing protein	0.00	G10641.20	0.00
A0A8B6DHE3	A0A8W8NDX0	Peptidase C1A papain C-terminal domain-containing protein	1.20×10^{-176}	G5397.18	9.41×10^{-177}
A0A6J8A856	K1PGZ1	Testis-specific gene 10 protein	0.00	G35361.3	0.00
A0A8B6EZW6	A0A8W8KMV0	Cilia- and flagella-associated protein 45	0.00	G24574.5	0.00
A0A8B6C153	A0A8W8MWR9	Citrate synthase	0.00	G35409.40	0.00
A0A8B6CP95	K1R1A5	Collagen alpha-1(IV) chain	0.00	G15188.2	9.46×10^{-120}
A0A8B6FHM0	K1RH30	Collagen alpha-1(IV) chain	1.65×10^{-39}	G9790.1	1.61×10^{-37}
A0A8S3UY90	A0A8W8K042	Rootletin	0.00	G21075.1	0.00
A0A8B6C6M2	K1R702	Body wall muscle protein HR-29	1.07×10^{-48}	G26545.1	9.92×10^{-49}
A0A6J8D0X9	A0A8W8JHD7	Fibropellin-1	0.00	G1940.1	0.00
A0A8S3QII9	K1Q7M2	Cathepsin L	0.00	G9706.23	0.00
A0A8B6D685	A0A8W8I7C9	Dihydrolipoyllysine-residue succinyltransferase component of 2-oxoglutarate dehydrogenase complex. mitochondrial	1.36×10^{-159}	G12941.5	1.06×10^{-159}
A0A8S3RHZ5	A0A8W8IR75	Disks large-like protein 1	0.00	G15080.9	0.00
A0A8B6DX16	A0A8W8N7H8	Dolichyl-diphosphooligosaccharide--protein glycosyltransferase subunit 2	0.00	G4985.2	0.00
A0A8B6FGA8	K1PAG1	Dynein beta chain. ciliary	0.00	G761.1	0.00
A0A8S3RF13	A0A8W8LWE6	Transketolase	0.00	G30122.32	0.00
A0A8S3R8F7	A0A8W8JPQ6	Arginine kinase	1.14×10^{-178}	G19705.1	8.89×10^{-179}
A0A6J8A1B7	A0A8W8N948	Elongation factor 1-alpha	0.00	G4818.20	0.00
A0A8B6EQ50	K1PY28	Sarcoplasmic calcium-binding protein	1.97×10^{-39}	G32504.6	8.87×10^{-39}
A0A8B6CPN1	K1R0G7	Fibropellin-1 (Fragment)	7.73×10^{-83}	G5363.1	4.06×10^{-80}
A0A8B6FH66	A0A8W8I0T1	Elongation factor 1-gamma	0.00	G11860.32	0.00
A0A8B6EYD8	A0A8W8HXZ3	Elongation factor 2	0.00	G11483.21	0.00
A0A8B6FQU1	A0A8W8N1K3	FERM domain-containing protein	1.09×10^{-163}	G3572.64	8.55×10^{-164}

A0A8B6FTA2	K1RKC1	Far upstream element-binding protein 3	$2.43x10^{-169}$	G12312.1	$2.51x10^{-12}$
A0A8B6GX54	A0A8W8M883	Fibulin-1	0.00	G31035.19	0.00
A0A8B6H907	A0A8W8J8A2	Filamin-A	0.00	G1715.4	0.00
A0A8B6DL75	A0A8W8IQJ7	Calponin-homology (CH) domain-containing protein	0.00	G15398.3	0.00
A0A8B6E885	A0A8W8LLG1	Gelsolin-like domain-containing protein	$2.41x10^{-160}$	G28864.12	$1.88x10^{-160}$
A0A8B6BF20	A0A8W8NBH2	glutathione transferase	$4.33x10^{-86}$	G5106.4	$3.38x10^{-86}$
A0A8B6DZ81	K1Q350	Glyceraldehyde-3-phosphate dehydrogenase	0.00	G4037.3	0.00
A0A8B6DYX8	K1QIR8	78 kDa glucose-regulated protein	0.00	G29360.3	0.00
C0Z203	A0A8W8HTN3	Histidine kinase/HSP90-like ATPase domain-containing protein	0.00	G10994.4	0.00
A0A8B6BR27	A5LGG7	Glucose-regulated protein 94	0.00	G30100.1	0.00
A0A8B6GHQ6	A0A8W8KZ99	Ig-like domain-containing protein	0.00	G25939.1	0.00
A0A8B6H9V2	A0A8W8KZ99	Ig-like domain-containing protein	0.00	G25939.1	0.00
A0A0K0YAY3	A0A8W8K0M3	Basement membrane-specific heparan sulfate proteoglycan core protein	0.00	G21617.9	0.00
A0A8B6EPK5	A0A8W8J5R5	RRM domain-containing protein	0.00	G16953.11	0.00
A0A0L8GYE0	A0A8W8K8G0	Histone H4	$6.13x10^{-49}$	G22554.1	$4.79x10^{-49}$
A0A8S3VDI8	A0A8W8HNN7	Peroxisomal multifunctional enzyme type 2	0.00	G10373.3	0.00
A0A8B6CAW3	A0A8W8MGF9	K Homology domain-containing protein	0.00	G32958.1	0.00
A0A8B6BT14	A0A8W8I0U1	Large ribosomal subunit protein uL4 C-terminal domain-containing protein	0.00	G11965.6	0.00
A0A8S3QSD1	K1PQ23	PDZ and LIM domain protein 5	$1.24x10^{-172}$	G2996.3	$8.11x10^{-110}$
A0A8B6EFR5	A0A8W8IRY8	Cytosol aminopeptidase domain-containing protein	0.00	G15308.19	0.00
A0A8B6GXC9	K1QNP7	LIM and SH3 domain protein Lasp	$1.01x10^{-109}$	G3888.9	$5.23x10^{-52}$
A0A8S3VFX6	K1QT91	LIM domain and actin-binding protein 1	0.00	G26782.9	$4.98x10^{-13}$
A0A8S3PRL2	A0A8W8IU92	Lethal(2) giant larvae protein-like protein 1	0.00	G1588.7	0.00
A0A8S3Q6R0	K1RFA3	Lamin Dm0	0.00	G11465.2	0.00
A0A8S3TFK4	K1PU26	Malate dehydrogenase (Fragment)	0.00	G13589.13	$1.84x10^{-62}$
A0A8B6C2Y2	A0A8W8K8K1	Malate dehydrogenase. mitochondrial	$2.45x10^{-162}$	G22856.16	$1.91x10^{-162}$
A0A8B6DBF4	A0A8W8NN09	Metalloendopeptidase	$8.22x10^{-113}$	G6474.29	$6.42x10^{-113}$
A0A8B6FW38	A0A8W8J1A5	Stress-70 protein. mitochondrial	0.00	G16610.7	0.00
A0A8B6F2T3	A0A8W8HZQ8	Calponin-homology (CH) domain-containing protein	0.00	G11852.2	0.00
A0A8B6EEA4	A0A8W8L2H7	Neural cell adhesion molecule 1	$1.40x10^{-144}$	G26071.5	$1.09x10^{-144}$
A0A8B6HFZ0	A0A8W8LQI9	non-specific serine/threonine protein kinase	0.00	G29219.1	0.00
A0A8B6ETB5	K1PQP2	Serine/arginine-rich splicing factor 2	$7.07x10^{-41}$	G23736.5	$2.45x10^{-14}$
A0A8B6DGA0	K1PSE7	Outer dense fiber protein 3	$2.64x10^{-159}$	G18379.13	$2.06x10^{-159}$
A0A8B6D9K2	---NA---	---NA---	---NA---	---NA---	---NA---
A0A8S3RZQ4	A0A8W8J283	Parkin coregulated gene protein	$2.25x10^{-154}$	G16920.1	$1.76x10^{-154}$
A0A8B6DTM2	A0A8W8IGZ9	PDZ domain-containing protein	$3.91x10^{-59}$	G1399.61	$3.05x10^{-59}$

A0A6J8B435	A0A8W8HZM2	Fibrillin-2	0.00	G11837.1	0.00
A0A8B6F8H6	A0A8W8LA78	Thioredoxin domain-containing protein	$2.77x10^{-115}$	G27091.1	$2.17x10^{-115}$
A0A8B6FIW7	K1QYT5	Phosphate carrier protein. mitochondrial	0.00	G30148.5	0.00
A0A8B6CG53	K1QEA6	phosphoenolpyruvate carboxykinase (GTP)	0.00	G23781.36	0.00
A0A8B6HP01	A0A8W8KGB3	phosphoenolpyruvate mutase	0.00	G23366.8	0.00
A0A8B6C763	A0A8W8NS22	Plastin-2	0.00	G6498.41	0.00
A0A8B6CYA7	K1Q639	Receptor-type tyrosine-protein phosphatase T	0.00	G12173.1	0.00
A0A8S3QR29	A0A8W8JX77	thioredoxin-dependent peroxiredoxin	$1.32x10^{-124}$	G2146.6	$1.03x10^{-124}$
A0A8B6BJW5	A0A8W8K0N9	Prohibitin	$1.81x10^{-141}$	G21620.10	$1.42x10^{-141}$
A0A8B6DIX4	A0A8W8LQN5	Glutathione S-transferase 1	$1.40x10^{-55}$	G29525.1	$1.09x10^{-55}$
A0A8B6EJ02	K1Q6X5	Protein disulfide-isomerase	0.00	G1339.29	0.00
A0A8B6G1S8	A0A8W8I1E8	Protein disulfide-isomerase	0.00	G11932.5	0.00
A0A6J8BIG8	K1RV41	Small ribosomal subunit protein RACK1	0.00	G3645.44	0.00
A0A8B6D4L6	K1PUJ1	Moesin/ezrin/radixin homolog 1	0.00	G17750.31	0.00
A0A3G1CJN6	A0A8W8MQ51	Small ribosomal subunit protein eS1	$6.02x10^{-169}$	G34618.1	$4.71x10^{-169}$
A0A8B6C4T1	A0A8W8MU34	RNA helicase	0.00	G35350.2	0.00
A0A8B6DTX0	A0A8W8K042	Rootletin	0.00	G21075.1	0.00
A0A8B6BF70	K1Q8F7	Alpha-crystallin B chain	$1.40x10^{-56}$	G34560.2	$1.67x10^{-55}$
A0A8B6DCE2	A0A8W8LEA1	40S ribosomal protein S3	$3.11x10^{-161}$	G27592.12	$2.43x10^{-161}$
A0A8S3QQF6	K1R0L4	Sodium/potassium-transporting ATPase subunit alpha	0.00	G25027.37	0.00
A0A126Q9D5	A0A8W8JHY1	Staphylococcal nuclease domain-containing protein 1	0.00	G18611.7	0.00
A0A6J8B610	A0A8W8I6N1	Stress-induced-phosphoprotein 1	0.00	G12646.14	0.00
A0A6J8DUP3	A0A8W8KYL1	Band 7 domain-containing protein	$6.16x10^{-142}$	G25137.36	$4.81x10^{-142}$
A0A8S3TEG9	A0A8W8N2K5	Succinate dehydrogenase	0.00	G5034.22	0.00
A0A6J8ESE0	A0A8W8N942	Calponin-homology (CH) domain-containing protein	$1.11x10^{-85}$	G4815.29	$8.66x10^{-85}$
A0A8B6GHP0	A0A8W8IGH4	Tektin	0.00	G1416.3	0.00
A0A8B6DFA7	A0A8W8KM53	Thymosin beta-4	$1.19x10^{-62}$	G24500.65	$9.33x10^{-63}$
Q966V3	A0A8W8LCL2	Transgelin	0.00	G27652.16	0.00
A0A8B6H439	A0A8W8ILL9	Transglutaminase-like domain-containing protein	0.00	G14594.1	0.00
A0A6J8BFQ0	A0A8W8MB90	Trichohyalin-plectin-homology domain-containing protein	0.00	G31463.4	0.00
A0A8B6BTY3	A0A8W8JZQ8	Triosephosphate isomerase	$4.27x10^{-150}$	G21794.2	$3.34x10^{-150}$
A0A8B6F8B1	A0A8W8HL87	Tropomyosin	$3.39x10^{-125}$	G10110.138	$2.65x10^{-125}$
A0A3S1A1H2	A0A8W8MF40	Tubulin alpha chain	0.00	G32475.58	0.00
A0A8B6BWE2	K1R278	Tubulin beta chain	0.00	G24506.8	0.00
A0A6J8CNX6	A0A8W8MKL6	Tubulin polymerization-promoting protein family member 3	$4.05x10^{-39}$	G3391.16	$3.17x10^{-39}$
A0A8B6BL03	K1QLS3	Cytochrome b-c1 complex subunit 2. mitochondrial	$2.36x10^{-45}$	G331.9	$2.51x10^{-42}$

A0A8B6HIV7	K1PBC0	Non-neuronal cytoplasmic intermediate filament protein	0.00	G24263.8	2.20x10^{-168}
A0A8B6EIG0	K1QUX9	Prolow-density lipoprotein receptor-related protein 1	5.24x10^{-26}	G27135.2	2.22x10^{-25}
A0A8B6DFD7	A0A8W8HVW8	Papilin	3.74x10^{-21}	G11186.1	2.92x10^{-21}
A0A8B6G096	A0A8W8KRU1	Uncharacterized protein	7.14x10^{-16}	G2504.5	5.58x10^{-16}
A0A8B6E104	K1PSR5	Fatty acid-binding protein. heart	3.26x10^{-34}	G21524.4	2.90x10^{-34}
A0A8B6EM75	K1QPB7	Uncharacterized protein	2.03x10^{-50}	G23239.2	4.28x10^{-21}
A0A8B6C479	A0A8W8LS81	Ependymin-related protein 1	5.49x10^{-24}	G294.3	4.29x10^{-24}
A0A6J8CV26	A0A8W8NFT4	Extended synaptotagmin-2	0.00	G6772.4	0.00
A0A8B6E647	A0A8W8J1Z0	Kielin/chordin-like protein	0.00	G16891.13	0.00
A0A8B6GX65	K1QTV9	Collagen alpha-4(VI) chain	0.00	G21248.1	0.00
A0A6J8AGA3	---NA---	---NA---	---NA---	---NA---	---NA---
A0A8S3SZC2	A0A8W8IAW2	Mesenchyme-specific cell surface glycoprotein	8.33x10^{-160}	G13376.4	6.51x10^{-160}
A0A8B6DPQ0	K1R459	SCO-spondin	0.00	G35504.1	1.30x10^{-133}
A0A8B6DC97	A0A8W8NN09	Metalloendopeptidase	6.29x10^{-128}	G6474.29	4.91x10^{-128}
A0A6J8BUN8	A0A8W8LRT0	Tenascin-X	2.74x10^{-06}	G29424.4	2.14x10^{-06}
A0A8B6GIV6	K1QJ28	Mammalian ependymin-related protein 1	1.09x10^{-89}	G12752.5	7.87x10^{-63}
A0A8B6G9E5	K1QT65	Collagen alpha-3(VI) chain	1.36x10^{-49}	G21249.19	3.83x10^{-49}
A0A8B6CB58	A0A8W8JRD3	Apextrin C-terminal domain-containing protein	2.36x10^{-08}	G20570.1	1.84x10^{-08}
A0A8B6BI55	A0A8W8MX28	Vinculin	0.00	G35429.17	0.00
A0A8B6BU43	K1PKC1	Collagen alpha-6(VI) chain	2.30x10^{-88}	G4540.1	2.12x10^{-79}
A0A8S3TBN4	A0A8W8JCM3	CSD domain-containing protein	3.06x10^{-15}	G18521.3	2.39x10^{-15}
A0A8S3PR18	K1P9N7	14-3-3 protein zeta	7.96x10^{-117}	G23866.50	3.06x10^{-101}
A0A8B6EBM4	K1QET2	Coatomer subunit alpha	0.00	G3968.1	0.00
A0A8S3VPZ7	A0A8W8KS23	Coatomer subunit delta	0.00	G25063.1	0.00
A0A8B6CVU1	D7EZG8	Actin-depolymerizing factor 6	3.47x10^{-24}	G23042.5	2.71x10^{-24}
A0A8B6BSQ3	A0A8W8I947	Coiled-coil domain-containing protein 170	0.00	G13159.9	0.00
A0A8B6FD83	K1PDS7	Collagen alpha-2(I) chain	0.00	G5252.2	5.45x10-67
A0A8B6EZ83	K1RSS9	Collagen alpha-5(VI) chain	7.16x10^{-50}	G3584.8	5.59x10^{-50}
A0A8B6CQJ1	K1QYS9	Reelin domain-containing protein	1.23x10^{-43}	G21989.2	9.60x10^{-44}
A0A8B6CAW5	K1QYS9	Reelin domain-containing protein	1.75x10^{-24}	G21989.2	1.37x10^{-24}
A0A8S3Q3L3	K1RAD3	Cysteine-rich secretory protein LCCL domain-containing 2	2.05x10^{-92}	G1476.1	8.71x10^{-80}
A0A6J8BMS7	A0A8W8J450	SH3 domain-containing protein	0.00	G17159.4	0.00
A0A8S3RLA1	A0A8W8M1D6	CUB domain-containing protein	7.88x10^{-28}	G31121.1	4.47x10^{-26}
A0A8S3RUL4	A0A8W8LT11	cystathionine gamma-lyase	0.00	G2948.1	0.00
A0A8B6HEV7	A0A8W8NFU0	LIM zinc-binding domain-containing protein	3.22x10^{-96}	G5979.9	2.51x10^{-96}
A0A6J8AEC3	A0A8W8LZS3	EGF-like domain-containing protein	1.39x10^{-108}	G30352.4	1.09x10^{-108}
A0A8B6F031	K1QKV1	Cytochrome b-c1 complex subunit 6. mitochondrial	4.13x10^{-12}	G2226.1	3.23x10^{-12}
A0A8B6G281	K1PZT2	Cytochrome c oxidase subunit 5B. mitochondrial	1.34x10^{-14}	G2133.2	1.05x10^{-14}
A0A8B6EWU5	K1PSR5	Fatty acid-binding protein. heart	2.37x10^{-60}	G21524.4	1.85x10^{-60}
A0A8B6H151	K1R406	Death-associated protein 1	8.13x10^{-30}	G25521.2	6.35x10^{-30}
A0A077H0R0	---NA---	---NA---	---NA---	---NA---	---NA---

A0A8B6GTY8	K1QYD8	Trans-1.2-dihydrobenzene-1.2-diol dehydrogenase	$1.65x10^{-103}$	G15600.7	$1.77x10^{-103}$
A0A8B6DQH5	A0A8W8LVQ0	Dihydrolipoyl dehydrogenase	0.00	G29337.12	0.00
A0A6J8BTF0	A0A8W8NK98	33 kDa inner dynein arm light chain. axonemal	$4.58x10^{-170}$	G6194.23	$3.58x10^{-170}$
A0A8B6BL12	A0A8W8K2R5	Dolichyl-diphosphooligosaccharide--protein glycosyltransferase subunit DAD1	$3.21x10^{-63}$	G22173.1	$2.50x10^{-63}$
A0A8B6H0X9	K1R3G2	Drebrin-like protein	$5.52x10^{-151}$	G16042.3	$4.16x10^{-69}$
A0A8B6FMV8	---NA---	---NA---	---NA---	---NA---	---NA---
A0A6J8DUW4	K1PHB6	DUF4476 domain-containing protein	$2.03x10^{-123}$	G22182.3	$1.59x10^{-123}$
A0A6J8BZ13	K1QRD0	Dynein light intermediate chain	0.00	G21392.2	0.00
A0A8B6G9Z7	A0A8W8LSU0	E1 ubiquitin-activating enzyme	0.00	G29533.5	0.00
A0A8B6EYR0	A0A8W8ISY1	EF-hand domain-containing family member C2	0.00	G15272.5	0.00
A0A8B6CHC6	A0A8W8MAQ3	EF-hand domain-containing protein	$4.15x10^{-22}$	G32594.10	$3.24x10^{-22}$
A0A3G1CJM7	Q70MN9	Myosin essential light chain (Fragment)	$2.92x10^{-90}$	G14845.4	$1.19x10^{-89}$
A0A3R5Q145	A0A8W8LLN7	EF-hand domain-containing protein	$3.35x10^{-78}$	G2857.8	$2.61x10^{-78}$
A0A8B6GTM1	A0A8W8NHG0	EF-hand domain-containing protein	$2.79x10^{-163}$	G6194.19	$2.18x10^{-163}$
A0A8B6BHU6	A0A8W8L145	EF-hand domain-containing protein	$7.35x10^{-85}$	G26141.4	$5.74x10^{-85}$
A0A8B6EJE1	K1QAD2	EF-hand domain-containing protein	$1.54x10^{-68}$	G6522.1	$1.20x10^{-68}$
A0A8B6DVI4	A0A8W8K6F9	Fibropellin-1	$1.46x10^{-99}$	G22152.4	$1.14x10^{-99}$
A0A8S3RV51	A0A8W8JQE5	EGF-like domain-containing protein	$1.64x10^{-168}$	G2048.1	$1.28x10^{-168}$
A0A6J8CPT2	A0A8W8HS69	Electron transfer flavoprotein subunit alpha	$7.16x10^{-180}$	G10765.1	$5.59x10^{-180}$
A0A8S3V871	K1PWP8	Echinoderm microtubule-associated protein-like 1	0.00	G22103.1	$4.60x10^{-60}$
A0A8B6C964	K1RU07	Glucose-repressible alcohol dehydrogenase transcriptional effector	$1.71x10^{-149}$	G18106.3	$3.01x10^{-54}$
A0A8B6H228	A0A8W8KVI2	Thioredoxin domain-containing protein	0.00	G25203.7	0.00
A0A8S3VIN2	A0A8W8KCF7	Enolase	$1.10x10^{-70}$	G22912.4	$8.59x10^{-71}$
A0A8B6CMG1	K1QSX8	enoyl-CoA hydratase	0.00	G245.6	0.00
A0A8B6GL66	A0A8W8NDW1	SH3 domain-containing protein	0.00	G6496.3	0.00
A0A6J8D3C7	A0A8W8J6N8	Band 7 domain-containing protein	0.00	G1706.5	0.00
A0A6J8EG86	A0A8W8HZ31	Eukaryotic translation initiation factor 3 subunit B	0.00	G1175.2	0.00
A0A8S3RJH3	A0A8W8MYR0	Eukaryotic translation initiation factor 3 subunit I	0.00	G3659.10	0.00
A0A8S3PS95	A0A8W8KDB3	Eukaryotic translation initiation factor 5A	$1.18x10^{-77}$	G23412.4	$9.25x10^{-78}$
A0A8S3VLQ5	K1PLV6	F-actin-capping protein subunit alpha	$1.48x10^{-172}$	G29303.22	$1.16x10^{-172}$
A0A8B6DPU0	A0A8W8N171	FABP domain-containing protein	$1.58x10^{-20}$	G35511.6	$1.24x10^{-20}$
A0A8B6DQX4	K1QM68	Cytochrome P450 3A29	$4.19x10^{-09}$	G35510.1	$3.28x10^{-09}$
A0A8B6BMA1	K1QM68	Cytochrome P450 3A29	$2.73x10^{-15}$	G35510.1	$2.13x10^{-15}$
A0A8B6E270	K1PXW8	Transforming growth factor-beta-induced protein ig-h3	$5.92x10^{-125}$	G26746.4	$4.62x10^{-125}$
A0A8B6F6T7	K1R2K2	Periostin	$2.17x10^{-104}$	G29366.1	$1.69x10^{-104}$
A0A6J8D3U3	K1QEZ3	Fascin	0.00	G718.5	0.00
A0A8B6G0F8	A0A8W8LV63	Fibronectin type-III domain-containing protein	0.00	G29757.2	0.00
A0A8B6EE15	A0A8W8LWV5	10-formyltetrahydrofolate dehydrogenase	0.00	G29489.1	0.00

A0A8S3UCU2	A0A8W8MM64	Alcohol dehydrogenase class-3	$3.07x10^{-103}$	G3500.19	$2.39x10^{-103}$
A0A8S3TR72	K1RTQ6	Fructose-bisphosphate aldolase	0.00	G34645.19	0.00
A0A8B6BVI8	A0A8W8MQD3	Fructose-bisphosphate aldolase	0.00	G34649.12	0.00
A0A8S3Q6H6	A0A8W8P139	Fumarylacetoacetase	0.00	G8930.1	0.00
A0A0C5Q4G0	A0A8W8I5I4	Galectin	$1.06x10^{-130}$	G12471.17	$8.26x10^{-131}$
A0A8B6CKU6	A0A8W8KLR8	Gasdermin pore forming domain-containing protein	$7.54x10^{-68}$	G24166.4	$5.89x10^{-68}$
A0A8B6D0L8	A0A8W8M5G4	Gelsolin-like domain-containing protein	0.00	G31264.12	0.00
A0A8S3QNI2	K1PTI6	Glucose-6-phosphate isomerase	0.00	G20982.5	0.00
A0A8B6BHJ8	A0A8W8L0L3	Glucosidase 2 subunit beta	0.00	G2609.11	0.00
A0A8B6DQP9	K1QRQ2	glutamate dehydrogenase	0.00	G3271.17	0.00
A0A8S3Q5G9	A0A8W8K6J4	Glutathione S-transferase omega	$1.09x10^{-81}$	G21877.3	$8.48x10^{-82}$
A0A6J8BZR7	K1P979	Glyoxalase domain-containing protein 4	$1.54x10^{-170}$	G21694.2	$1.20x10^{-170}$
A0A8S3VGU6	A0A8W8KIR2	GMP reductase	0.00	G23328.11	0.00
A0A6J8ENV8	K1PWZ3	Guanine nucleotide-binding protein subunit beta	0.00	G22114.37	0.00
A0A8S3R8I4	A0A8W8KX36	PDZ GRASP-type domain-containing protein	$6.98x10^{-137}$	G24970.8	$5.46x10^{-137}$
A0A6J8F5H8	K1RAL0	aspartate transaminase	0.00	G16087.4	0.00
A0A2H4WCW9	K1QAV0	Guanine nucleotide-binding protein subunit alpha	0.00	G9338.1	0.00
A0A6J8CA81	K1QVX4	Glycogen synthase kinase-3 beta	0.00	G25031.10	$3.29x10^{-87}$
A0A0L8I0K2	A0A8W8IC92	GTP-binding protein SAR1b	$1.45x10^{-126}$	G1334.14	$1.13x10^{-126}$
A0A8B6HQH7	A0A8W8KS31	Heat shock 70 kDa protein 4L	0.00	G24810.12	0.00
A0A411G5T5	A0A8W8KZI1	Ig-like domain-containing protein	$2.17x10^{-10}$	G25938.1	$1.70x10^{-10}$
A0A8B6D225	A0A8W8NBT7	RRM domain-containing protein	$3.24x10^{-89}$	G5522.42	$2.53x10^{-89}$
A0A8B6ETA9	A0A8W8MJ95	H15 domain-containing protein	$8.63x10^{-43}$	G34589.4	$6.74x10^{-43}$
Q6WV85	K1R2L4	Histone H2B	$2.04x10^{-68}$	G22555.2	$1.01x10^{-64}$
A0A8S3RBA2	A0A8W8NEU6	RRM domain-containing protein	$1.01x10^{-71}$	G5522.28	$7.86x10^{-72}$
A0A6J8ESL8	A0A8W8HSK9	RRM domain-containing protein	$4.20x10^{-82}$	G1087.10	$3.28x10^{-82}$
A0A8S3U136	A0A8W8N6S3	hydroxyacid-oxoacid transhydrogenase	0.00	G5569.6	0.00
A0A8B6GQQ8	A0A8W8HSI3	Ig-like domain-containing protein	$2.49x10^{-13}$	G10784.34	$1.95x10^{-13}$
A0A8B6DGI1	A0A8W8M908	RRM domain-containing protein	0.00	G31955.1	0.00
A0A8B6CIE6	A0A8W8HL81	Isocitrate dehydrogenase	0.00	G10081.7	0.00
A0A8B6ET27	K1QAG0	Serine-threonine kinase receptor-associated protein	$6.66x10^{-60}$	G17845.1	$8.50x10^{-57}$
A0A6J8A1Y0	K1QJV9	BTB/POZ domain-containing protein KCTD16	$1.25x10^{-153}$	G26898.18	$9.73x10^{-154}$
A0A8B6FVU2	K1QUC7	Kinesin-related protein 1	0.00	G4885.1	0.00
A0A8B6G9Q8	A0A8W8JJV6	Laminin subunit alpha	0.00	G189.5	0.00
A0A8B6CXT1	A0A8W8MMB2	Laminin subunit beta-1	0.00	G35028.4	0.00
A0A8B6CGB4	A0A8W8MU73	Large ribosomal subunit protein uL1	$1.15x10^{-107}$	G34615.1	$9.01x10^{-108}$
A0A8B6F9E7	A0A8W8JVI9	Large ribosomal subunit protein eL22	$4.85x10^{-62}$	G20685.3	$3.79x10^{-62}$
A0A8B6BGD5	A0A8W8IQU1	Ribosomal protein L37	$4.46x10^{-37}$	G1543.3	$3.48x10^{-37}$
A0A8B6GGC2	A0A8W8KZ78	Large ribosomal subunit protein uL18 C-terminal eukaryotes domain-containing protein	$8.25x10^{-166}$	G25620.1	$6.44x10^{-166}$

A0A8B6E6I0	A0A8W8NCD2	60S ribosomal protein L7	9.51x10^{-144}	G5571.10	7.43x10^{-144}
A0A8B6BTE6	A0A8W8N7H9	Large ribosomal subunit protein P1	1.62x10^{-32}	G5649.7	1.26x10^{-32}
A0A6J8B8C6	A0A8W8LLZ8	Lipoma HMGIC fusion partner-like 3 protein	1.87x10^{-134}	G28860.1	1.46x10^{-134}
A0A8B6GHC2	K1P2H1	Fatty acid-binding protein. adipocyte	8.48x10^{-57}	G21693.2	6.63x10^{-57}
A0A0K0YB29	A0A8W8IPH2	Kyphoscoliosis peptidase	6.56x10^{-94}	G15010.18	5.12x10^{-94}
A0A8S3U3J8	K1R6E6	Basement membrane-specific heparan sulfate proteoglycan core protein	9.29x10^{-29}	G21617.9	7.39x10^{-29}
A0A8S3UA31	A0A8W8HVW8	Papilin	1.42x10^{-13}	G11186.1	1.11x10^{-13}
A0A8B6GPG7	A0A8W8J9G8	Membrane glycoprotein lig-1	5.47x10^{-116}	G17951.7	4.27x10^{-116}
A0A8B6G660	K1PG07	Lupus La-like protein	2.01x10^{-127}	G25932.1	1.57x10^{-127}
A0A8B6DCW7	A0A8W8KU66	MAGUK p55 subfamily member 6	0.00	G24992.5	0.00
A0A6J8CXM9	A0A8W8KCS5	Malectin domain-containing protein	1.06x10^{-124}	G23356.6	8.24x10^{-125}
A0A8B6D8H6	A0A8W8I653	Mammalian ependymin-related protein 1	1.13x10^{-74}	G12752.5	8.79x10^{-75}
A0A6J8EFL5	A0A8W8I304	Mammalian ependymin-related protein 1	7.23x10^{-74}	G12308.3	5.65x10^{-74}
A0A8S3RY54	A0A8W8J1M9	Microtubule-associated protein RP/EB family member 1	2.57x10^{-114}	G1666.6	2.01x10^{-114}
A0A8B6BF41	K1PR60	CKLF-like MARVEL transmembrane domain-containing protein 4	1.64x10^{-37}	G21838.6	6.59x10^{-36}
A0A8B6CZ83	K1PQP7	Mesenchyme-specific cell surface glycoprotein	3.48x10^{-128}	G13376.4	6.68x10^{-128}
Q697L7	Q9BIV4	Metallothionein	1.43x10^{-16}	G30623.10	4.47x10^{-15}
A0A8S3QX71	K1R252	methylmalonate-semialdehyde dehydrogenase (CoA acylating)	0.00	G13035.21	0.00
A0A6J8F1I0	A0A8W8MVH8	Mitochondrial carrier-like protein 2	1.19x10^{-86}	G35527.2	9.27x10^{-87}
A0A8B6H0Z6	K1Q1S3	Myosin-VI	0.00	G19685.4	0.00
A0A8B6EU84	A0A8W8MQE5	PDZ domain-containing protein	1.25x10^{-109}	G35457.24	9.80x10^{-110}
A0A8S3U4W4	A0A8W8MK96	NADP-dependent oxidoreductase domain-containing protein	3.54x10^{-175}	G32718.3	2.76x10^{-175}
A0A6J8BDN2	A0A8W8N229	Aldo-keto reductase family 1 member B1	2.84x10^{-170}	G4317.3	2.22x10^{-170}
A0A6J8CRB1	A0A8W8IK45	H/ACA ribonucleoprotein complex subunit 2	1.63x10^{-50}	G14693.2	1.28x10^{-50}
A0A8S3QGN5	K1QZ84	Thioredoxin domain-containing protein 3-like protein	0.00	G26290.2	0.00
A0A8S3PMK0	A0A8W8JP01	Nuclear migration protein nudC	6.71x10^{-125}	G19615.4	5.24x10^{-125}
A0A8B6C4N8	A0A8W8KLV1	Nucleoredoxin	6.96x10^{-57}	G24033.3	5.44x10^{-57}
A0A8S3ULS9	K1RHA5	Nucleoside diphosphate kinase	3.22x10^{-88}	G27377.8	1.12x10^{-72}
A0A8S3UGN8	A0A8W8KM88	Obg-like ATPase 1	0.00	G24214.1	0.00
A0A8B6EVV0	A0A8W8KI25	FHA domain-containing protein	2.56x10^{-137}	G23561.1	2.00x10^{-137}
A0A8B6BMW1	A0A8W8KKU1	Peptidase S1 domain-containing protein	1.61x10^{-65}	G24325.2	1.26x10^{-65}
A0A6J8DW13	A0A8W8NDY3	Thioredoxin domain-containing protein	0.00	G6502.1	0.00
A0A8S3TBS5	A0A8W8K954	PDZ domain-containing protein	7.71x10^{-62}	G22516.2	6.03x10^{-62}
A0A8B6DFV3	K1RHP3	Peptidase	0.00	G6069.10	0.00

https://doi.org/10.3390/xxxxx

A0A8B6D8S4	A0A8W8KK61	Peptidase metallopeptidase domain-containing protein	5.70x10^{-159}	G23496.3	4.45x10^{-159}
A0A8B6BIH5	A0A8W8N8J6	Peptidyl-prolyl cis-trans isomerase	3.77x10^{-90}	G5794.10	2.94x10^{-90}
A0A161I1V8	A0A8W8HZN8	Peptidyl-prolyl cis-trans isomerase	4.97x10^{-107}	G11826.2	3.88x10^{-107}
A0A6J8DKH3	A0A8W8IB78	Peptidyl-prolyl cis-trans isomerase	9.28x10^{-140}	G13223.1	7.25x10^{-140}
A0A8S3SEX5	A0A8W8HZN8	Peptidyl-prolyl cis-trans isomerase	8.07x10^{-121}	G11826.2	6.30x10^{-121}
A0A8B6EBJ6	K1QXK0	peptidylprolyl isomerase	4.43x10^{-44}	G2434.3	3.90x10^{-44}
A0A8S3RID5	A0A8W8N2J5	peptidylprolyl isomerase	6.84x10^{-171}	G4369.3	5.34x10^{-171}
A0A8B6D5H2	A0A8W8J0I5	Peroxidase	1.24x10^{-119}	G16328.2	9.67x10^{-120}
A0A8B6GKK6	K1R7F8	Peroxiredoxin-5	1.93x10^{-86}	G11001.1	9.73x10^{-51}
A0A8S3R7F6	A0A8W8IHJ9	phosphoglycerate mutase (2.3-diphosphoglycerate-dependent)	4.17x10^{-160}	G14096.8	3.26x10^{-160}
A0A8S3S8I1	K1PQD4	phosphoglucomutase (alpha-D-glucose-1.6-bisphosphate-dependent)	0.00	G1842.9	0.00
A0A6J7ZU18	A0A8W8L3H8	Phosphoglycerate kinase	0.00	G26194.4	0.00
A0A6J8AQ45	A0A8W8J208	Phospholipid scramblase	5.25x10^{-142}	G16506.13	4.10x10^{-142}
A0A6J8EPJ2	A0A8W8LD13	Phospholipid scramblase	3.96x10^{-123}	G27559.1	3.09x10^{-123}
A0A8B6DQL6	A0A8W8KBH8	Enolase	0.00	G22912.3	0.00
A0A8S3SL70	K1Q9P5	Mitochondrial-processing peptidase subunit beta	1.12x10^{-125}	G28532.5	1.27x10^{-125}
A0A6J8E914	A0A8W8LLK7	Cyclic nucleotide-binding domain-containing protein	0.00	G28586.3	0.00
A0A8B6FT25	---NA---	---NA---	---NA---	G1982.6	8.10x10^{-04}
A0A8B6CHE6	---NA---	---NA---	---NA---	---NA---	---NA---
A0A8B6D5R1	A0A8W8NKX3	BRO1 domain-containing protein	0.00	G6211.5	0.00
A0A8B6G5S6	A0A8W8L3N4	Prohibitin	5.25x10^{-155}	G26216.2	4.10x10^{-155}
A0A8B6CYK1	A0A8W8I619	Proteasome subunit alpha type	1.38x10^{-161}	G12565.1	1.08x10^{-161}
A0A8S3UDK6	A0A8W8J5Q6	Proteasome subunit alpha type	1.22x10^{-172}	G17351.2	9.55x10^{-173}
A0A8B6DC08	A0A8W8NEZ2	DEK-C domain-containing protein	2.20x10^{-28}	G553.4	1.58x10^{-29}
A0A8B6FXU3	A0A8W8MXV3	Thioredoxin domain-containing protein	0.00	G4424.4	0.00
A0A6J8DCE2	A0A8W8JZC3	Protein RCC2	0.00	G21740.3	0.00
A0A8B6EIQ6	K1PQY0	Protein sleepless	4.44x10^{-34}	G2186.6	3.47x10^{-34}
A0A8S3S9K8	A0A8W8LRP1	Protein SSUH2-like protein	5.01x10^{-158}	G28720.3	3.91x10^{-158}
A0A8B6CNR6	K1QB61	Protocadherin Fat 4	0.00	G2955.1	0.00
A0A8S3UA94	A0A8W8LP70	Proactivator polypeptide	9.39x10^{-164}	G2909.4	7.33x10^{-164}
A0A0G2YLG5	A0A8W8LGM6	Fibronectin type-III domain-containing protein	0.00	G27955.1	0.00
A0A6J8BU32	K1RWS2	Transcriptional activator protein Pur-alpha	2.25x10^{-152}	G6097.5	1.76x10^{-152}

https://doi.org/10.3390/xxxxx

A0A8B6BR62	A0A8W8JG17	Pyruvate dehydrogenase E1 component subunit alpha	0.00	G19054.7	0.00
A0A8S3QRM1	K1PFL3	Dihydropteridine reductase	1.85x10^{-122}	G14476.1	1.45x10^{-122}
A0A8S3RSD5	A0A8W8LN62	Queuosine 5'-phosphate N-glycosylase/hydrolase	7.15x10^{-139}	G28796.1	5.59x10^{-139}
A0A6J8CI59	A0A8W8HWK6	Ras-related protein Rab-1A	4.79x10^{-139}	G11385.6	3.74x10^{-139}
A0A0B7AP29	K1QBM3	Ras-related protein Rab-2	1.18x10^{-140}	G21850.11	9.24x10^{-141}
A0A8B6FT60	A0A8W8IBB4	Radial spoke head protein 4-like protein A	0.00	G13256.3	0.00
A0A8B6GLW0	A0A8W8ICW5	Radial spoke head protein 9 homolog	1.14x10^{-152}	G13591.5	8.87x10^{-153}
A0A2C9JE50	A0A8W8P296	Ras-related protein Rab-11A	1.43x10^{-148}	G8381.5	1.12x10^{-148}
A0A6J8CUS4	K1QC78	Ras-related protein Rab^{-14}	3.22x10^{-160}	G27922.1	2.52x10^{-160}
A0A8B6G941	A0A8W8KBX5	Ras-related protein Rab-35	2.84x10^{-134}	G22846.2	2.22x10^{-134}
A0A6J8F240	A0A8W8HY86	RNA-binding protein 4	1.01x10^{-104}	G11518.6	7.90x10^{-105}
A0A8B6C295	A0A8W8HL97	Receptor expression-enhancing protein	1.73x10^{-89}	G10083.4	1.35x10^{-89}
A0A6J8DY17	K1PYT4	RIB43A-like with coiled-coils protein 2	0.00	G30050.2	0.00
A0A077H3K4	A0A8W8KKZ1	Large ribosomal subunit protein uL23 N-terminal domain-containing protein	5.58x10^{-75}	G24036.6	4.36x10^{-75}
A0A077H3J9	K1PM50	40S ribosomal protein S16	2.53x10^{-94}	G5555.1	5.35x10^{-94}
A0A8S3QYE0	A0A8W8L0E1	Profilin	1.73x10^{-42}	G26073.12	1.35x10^{-42}
A0A8B6FIE8	A0A8W8HY86	RNA-binding protein 4	5.43x10^{-21}	G11518.6	4.24x10^{-21}
A0A6J8A0F1	Q70MN8	Large ribosomal subunit protein eL24 (Fragment)	3.74x10^{-69}	G18702.4	3.41x10^{-69}
A0A8S3UK82	A0A8W8IP93	Large ribosomal subunit protein uL24	3.84x10^{-89}	G15236.1	3.00x10^{-89}
A0A8B6EXY4	A0A8W8MB64	RRM domain-containing protein	0.00	G32666.2	0.00
A0A8B6HNM7	A0A8W8MRK6	S-(hydroxymethyl)glutathione dehydrogenase	4.68x10^{-159}	G3500.16	3.65x10^{-159}
A0A8B6CEB0	A0A8W8KAL0	Saposin B-type domain-containing protein	2.33x10^{-136}	G2310.4	1.82x10^{-136}
A0A6J8DVF9	A0A8W8M8E6	Translocon Sec61/SecY plug domain-containing protein	0.00	G31051.31	0.00
A0A8B6G4G0	A0A8W8JX33	Selenoprotein M	1.46x10^{-18}	G20897.10	1.14x10^{-18}
A0A8S3RKC5	K1RD83	Serine hydroxymethyltransferase	0.00	G3195.5	0.00
A0A8B6H826	A0A8W8IW86	SH3 domain-containing protein	0.00	G15848.1	0.00
A0A8B6GPC3	A0A8W8JB64	SHSP domain-containing protein	2.01x10^{-33}	G17984.2	1.57x10^{-33}
A0A8S3RCR9	K1QR48	Calcium-binding mitochondrial carrier protein SCaMC-2	0.00	G24895.1	0.00
A0A8B6F648	A0A8W8IE99	Small nuclear ribonucleoprotein Sm D2	7.72x10^{-80}	G13575.2	6.03x10^{-80}
A0A8B6CSI5	A0A8W8I406	40S ribosomal protein S17	2.69x10^{-88}	G12279.5	2.10x10^{-88}
A0A8B6HL62	K1QC22	40S ribosomal protein S19	7.29x10^{-81}	G26391.10	5.70x10^{-81}
A0A6J8BWT3	K1Q9D7	Sorting nexin-2	0.00	G10437.1	0.00
A0A8S3VFI8	A0A8W8MZ41	Spermatogenesis-associated protein 6 N-terminal domain-containing protein	4.92x10^{-110}	G3665.4	3.84x10^{-110}
A0A6J8D7K5	K1QTY5	Sulfide:quinone oxidoreductase. mitochondrial	3.59x10^{-171}	G7982.1	5.17x10^{-171}

A0A6J8E460	K1QCQ5	Succinate--CoA ligase	0.00	G16455.1	0.00
A0A8S3R089	A0A8W8INR7	Succinate--CoA ligase	0.00	G15171.5	0.00
A0A8B6CGG6	A0A8W8L0F8	Suppressor of G2 allele of SKP1-like protein	$7.21x10^{-134}$	G25356.4	$5.63x10^{-134}$
A0A8B6EYT5	A0A8W8JCM1	C2 domain-containing protein	$1.50x10^{-170}$	G17776.3	$1.17x10^{-170}$
A0A8B6DIG2	A0A8W8LAX1	T-complex protein 1 subunit alpha	0.00	G27437.1	0.00
A0A8B6E6D8	A0A8W8I1T4	T-complex protein 1 subunit gamma	0.00	G12090.2	0.00
A0A8B6F9J1	K1PXN5	T-complex protein 1 subunit zeta	0.00	G22682.5	0.00
A0A6J8BSI9	A0A8W8KQ90	Peptidase S54 rhomboid domain-containing protein	0.00	G2486.6	0.00
A0A6J8BBL7	A0A8W8N7D1	Tektin	0.00	G4594.6	0.00
A0A8S3SXV7	A0A8W8NSM9	Tetraspanin	$3.52x10^{-73}$	G6559.4	$2.75x10^{-73}$
A0A8B6CJW7	A0A8W8KN44	Tetraspanin	$7.89x10^{-15}$	G24614.1	$6.16x10^{-15}$
A0A8B6DN29	A0A8W8NIE7	Tetraspanin	$1.86x10^{-60}$	G5949.1	$1.45x10^{-60}$
A0A6J8AMT5	K1QIL7	Tetratricopeptide repeat protein 29	0.00	G25787.8	0.00
A0A8B6H5W0	A0A8W8L209	Thioredoxin	$2.59x10^{-37}$	G26313.13	$2.03x10^{-37}$
A0A8S3QJ24	A0A8W8P6T5	Thioredoxin-like protein 1	$2.81x10^{-153}$	G9681.1	$2.20x10^{-153}$
A0A8B6FN66	A0A8W8NVV8	Thyroglobulin type-1 domain-containing protein	$6.64x10^{-89}$	G6992.7	$5.19x10^{-89}$
A0A8B6C4T2	A0A8W8NHK5	Thyroglobulin type-1 domain-containing protein	$2.78x10^{-31}$	G6992.4	$2.17x10^{-31}$
A0A8S3R738	---NA---	---NA---	---NA---	---NA---	---NA---
A0A8S3R9Y2	A0A8W8MM55	NTR domain-containing protein	$2.74x10^{-14}$	G34317.1	$2.14x10^{-14}$
A0A8B6FQW6	A0A8W8J501	Transaldolase	0.00	G17273.18	0.00
A0A8B6FLR4	A0A8W8JW85	Transcription elongation factor	$3.77x10^{-122}$	G2126.2	$2.95x10^{-122}$
A0A8B6CWL2	K1PFT9	Transgelin	$1.80x10^{-62}$	G19321.16	$8.05x10^{-62}$
A0A6J7ZVG6	K1QB11	Transgelin	$2.51x10^{-94}$	G28066.5	$2.00x10^{-94}$
A0A8B6H8C4	A0A8W8IXX2	Tripeptidyl-peptidase 2	0.00	G16345.6	0.00
A0A8B6F7C4	B7XC66	Tropomyosin	$8.32x10^{-106}$	G10110.176	$8.40x10^{-106}$
A0A6J8DNP0	K1QRQ0	60 kDa SS-A/Ro ribonucleoprotein	0.00	G3264.20	0.00
A0A0K2D7M8	A0A8W8HUR9	Protocadherin Fat 4	$2.52x10^{-29}$	G11053.1	$1.97x10^{-29}$
A0A0B6Z2B0	A0A8W8LF37	Tubulin beta chain	$2.70x10^{-44}$	G27780.7	$2.11x10^{-44}$
A0A8B6DWZ2	A0A8W8IRA6	THD domain-containing protein	$4.36x10^{-41}$	G15086.1	$3.40x10^{-41}$
A0A8S3TTF4	A0A8W8N7F2	Tumor protein D54	$1.04x10^{-72}$	G4980.28	$8.11x10^{-73}$
A0A8B6FHC2	U5U0P0	Tyrosinase	$3.93x10^{-91}$	G20964.3	$1.06x10^{-90}$
A0A8B6FCM7	A0A8W8JQE5	EGF-like domain-containing protein	$9.21x10^{-60}$	G2048.1	$7.20x10^{-60}$
A0A8S3SPC1	A0A8W8HU54	Ubiquitin-associated protein 2	$8.01x10^{-156}$	G11062.3	$6.25x10^{-156}$
A0A8S3QZQ4	A0A8W8LWR9	E2 ubiquitin-conjugating enzyme	$2.01x10^{-63}$	G30479.2	$1.57x10^{-63}$
A0A8B6CGQ6	A0A8W8MQ70	Cytochrome b-c1 complex subunit Rieske. mitochondrial	$9.01x10^{-73}$	G35433.9	$7.04x10^{-73}$
A0A0B6Y8N5	K1PD36	Ubiquitin-ribosomal protein eL40 fusion protein	$7.62x10^{-91}$	G26976.2	$5.95x10^{-91}$
A0A6J8CSZ8	A0A8W8IFN0	NAD-dependent epimerase/dehydratase domain-containing protein	0.00	G14021.8	0.00
A0A8B6FG82	K1R1A5	Collagen alpha-1(IV) chain	$4.72x10^{-38}$	G9790.1	$8.19x10^{-36}$
A0A3L5TR65	A0A8W8HUR9	Protocadherin Fat 4	$1.68x10^{-16}$	G11053.1	$1.31x10^{-16}$
A0A8B6FNN6	K1S3Q2	Macrophage asialoglycoprotein-binding protein	$7.62x10^{-05}$	G15878.8	$1.10x10^{-04}$
A0A8B6G8E3	A0A8W8MB95	SCO-spondin	$4.99x10^{-125}$	G32173.6	$3.89x10^{-125}$

A0A8B6GHG6	---NA---	---NA---	---NA---	---NA---	---NA---
A0A8B6CU91	---NA---	---NA---	---NA---	---NA---	---NA---
A0A8B6CEC3	---NA---	---NA---	---NA---	---NA---	---NA---
A0A8B6HNQ5	A0A8W8P090	Uncharacterized protein	3.62x10^{-07}	G9683.12	2.82x10^{-07}
A0A8B6FSV1	A0A8W8J417	Ndr family protein	0.00	G16645.30	0.00
A0A8B6CTG8	---NA---	---NA---	---NA---	---NA---	---NA---
A0A8B6ENM5	---NA---	---NA---	---NA---	---NA---	---NA---
A0A8B6CCA0	K1PRV7	Profilin	2.31x10^{-05}	G16496.5	1.80x10^{-05}
A0A8B6DTT6	K1QFX2	Low-density lipoprotein receptor-related protein 1B	2.50x10^{-25}	G26996.2	4.16x10^{-25}
A0A6J8CD73	K1PYZ9	SCO-spondin	1.36x10^{-27}	G1987.2	4.76x10^{-27}
A0A8S3T9B8	A0A8W8I8Z4	UPAR/Ly6 domain-containing protein	1.13x10^{-25}	G15797.1	8.86x10^{-26}
A0A8S3RZM2	A0A8W8IPH2	Kyphoscoliosis peptidase	6.89x10^{-93}	G15010.18	5.38x10^{-93}
A0A8B6F5H6	---NA---	---NA---	---NA---	---NA---	---NA---
A0A8B6H837	---NA---	---NA---	---NA---	---NA---	---NA---
A0A8B6FQ26	K1PJB8	Guanylate cyclase	2.31x10^{-13}	G5735.2	1.81x10^{-13}
A0A8B6DR40	K1Q894	EF-hand domain-containing protein 1	0.00	G4319.2	0.00
A0A8B6BSD4	A0A8W8NWQ5	Counting factor associated protein D	0.00	G78.3	0.00
A0A8B6CRI3	K1R6E6	Basement membrane-specific heparan sulfate proteoglycan core protein	1.19x10^{-25}	G21617.9	1.40x10^{-25}
A0A8B6CM77	A0A8W8LS81	Ependymin-related protein 1	2.65x10^{-21}	G294.3	2.07x10^{-21}
A0A8B6DHU3	A0A8W8LRT0	Tenascin-X	2.00x10^{-26}	G29424.4	1.57x10^{-26}
A0A8B6CG35	K1Q8V3	Ependymin-related protein 1	1.66x10^{-09}	G21093.10	1.30x10^{-09}
A0A8S3RSI4	---NA---	---NA---	---NA---	---NA---	---NA---
A0A8B6DI09	K1RN59	Laminin-like protein epi-1	0.00	G3871.5	0.00
A0A8B6CVX7	K1PBN9	Importin subunit alpha-2	6.21x10^{-08}	G13492.1	1.83x10^{-06}
A0A8B6HFY8	---NA---	---NA---	---NA---	---NA---	---NA---
A0A8B6F299	A0A167GGF3	9BACLWIAG-tail domain	3.60x10^{-07}	---NA---	---NA---
A0A8B6EZW5	K1RSS9	Collagen alpha-5(VI) chain	1.60x10^{-37}	G25577.1	4.30x10^{-37}
A0A8B6C5E2	K1PX59	Monocarboxylate transporter 12	1.66x10^{-21}	G13874.1	1.30x10^{-21}
A0A6J8CCF5	A0A8W8KT22	NodB homology domain-containing protein	7.72x10-120	G24491.7	6.03x10-120
A0A8S3U5X7	A0A8W8J3I6	Kielin/chordin-like protein	5.07x10^{-41}	G16891.3	8.25x10^{-37}
A0A8B6C9P4	---NA---	---NA---	---NA---	---NA---	---NA---
A0A8B6H7C0	A0A8W8I820	VWFD domain-containing protein	4.19x10^{-92}	G12919.1	3.27x10^{-92}
A0A8B6G6F1	K1RSH8	von Willebrand factor D and EGF domain-containing protein	0.00	G16100.1	0.00
A0A8B6FPE6	---NA---	---NA---	---NA---	---NA---	---NA---
A0A8S3S991	A0A8W8LRI1	Mammalian ependymin-related protein 1	5.94x10^{-22}	G287.2	4.64x10^{-22}
A0A8B6BE54	A0A8W8I3X0	PLAT domain-containing protein	5.27x10^{-07}	G12270.1	4.11x10^{-07}
A0A8B6GK87	A0A8W8MXH1	SCO-spondin	0.00	G35504.1	0.00
A0A8S3SVU1	A0A8W8LYZ9	Sperm microtubule inner protein 1 C-terminal domain-containing protein	1.22x10^{-102}	G30500.1	9.50x10^{-103}
A0A8B6D1E0	K1PWD9	Uncharacterized protein	2.36x10^{-06}	G5080.3	1.84x10^{-06}
A0A8B6CXZ8	A0A8W8JKX2	Ciliary microtubule inner protein 2C	1.32x10^{-124}	G1996.1	1.03x10^{-124}
A0A8B6HI74	A0A8W8ID54	Uncharacterized protein	1.08x10^{-75}	G13413.1	8.43x10^{-76}
A0A6J8EM21	A0A8W8KIJ1	Phosphatidylinositol transfer protein beta	9.11x10^{-155}	G24041.8	7.11x10^{-155}
A0A8S3UJ52	A0A8W8MPX9	Ig-like domain-containing protein	1.11x10^{-36}	G34792.1	8.65x10^{-37}

A0A8B6D1P6	A0A8W8KIA0	Beta-microseminoprotein	8.57×10^{-05}	G24018.2	6.69×10^{-05}
A0A8B6EZY7	A0A8W8MB95	SCO-spondin	0.00	G32173.6	0.00
A0A8S3QP16	A0A8W8J2V0	Uncharacterized protein	9.17×10^{-106}	G16810.7	7.16×10^{-106}
A0A8S3UR85	---NA---	---NA---	---NA---	---NA---	---NA---
A0A8S3SPV4	A0A8W8KQP5	IgGFc-binding protein N-terminal domain-containing protein	3.12×10^{-35}	G24885.2	2.44×10^{-35}
A0A8S3TKR9	K1QAZ7	Coiled-coil domain-containing protein 55	2.86×10^{-11}	G22155.2	3.14×10^{-11}
A0A8B6FV73	A0A8W8J7T4	LIM zinc-binding domain-containing protein	0.00	G17454.23	0.00
A0A8S3S0N2	A0A8W8JQB4	Sushi. von Willebrand factor type A. EGF and pentraxin domain-containing protein 1	0.00	G2047.1	0.00
A0A8S3QG66	---NA---	---NA---	---NA---	---NA---	---NA---
A0A8B6GYD1	K1QW43	Serine/threonine-protein phosphatase 6 regulatory ankyrin repeat subunit B	2.21×10^{-76}	G32429.1	1.73×10^{-76}
A0A8S3U726	K1QJ28	Mammalian ependymin-related protein 1	4.49×10^{-73}	G12308.3	1.04×10^{-58}
A0A8S3QIJ9	A0A8W8MWU0	UspA domain-containing protein	2.61×10^{-87}	G34954.1	2.04×10^{-87}
A0A8B6G9T0	A0A8W8LQ90	UspA domain-containing protein	1.66×10^{-43}	G29172.5	1.29×10^{-43}
A0A8B6ESB0	A0A8W8ICU3	UTP--glucose-1-phosphate uridylyltransferase	0.00	G13586.6	0.00
A0A8S3QEM3	K1QI28	Vacuolar proton pump subunit B	0.00	G15131.8	0.00
A0A6J8DNW3	A5LGH1	Voltage-dependent anion channel	1.73×10^{-137}	G1931.16	1.13×10^{-48}
A0A8B6E1P9	K1QVB4	Vitelline membrane outer layer protein 1	4.00×10^{-46}	G19499.2	1.87×10^{-45}
A0A8B6DLG6	A0A8W8P1D5	Serine carboxypeptidase CPVL	0.00	G8154.4	0.00
A0A8B6C9L4	A0A8W8KJ84	VWFA domain-containing protein	8.69×10^{-106}	G23385.8	6.79×10^{-106}
A0A8B6HDB4	K1Q662	Actin-interacting protein 1	0.00	G15632.1	0.00
A0A8B6F1U6	K1RDT2	Cilia- and flagella-associated protein 52	0.00	G3365.1	0.00
A0A8B6CY00	A0A8W8HLX2	2-oxoglutarate dehydrogenase. mitochondrial	0.00	G1020.16	0.00
A0A210Q521	A0A8W8IDA4	AAA domain-containing protein	0.00	G13469.5	0.00
A0A8B6GS06	A0A8W8IJT2	26S proteasome regulatory subunit RPN2 C-terminal domain-containing protein	3.12×10^{-162}	G14316.3	2.44×10^{-162}
A0A8B6GHX0	A0A8W8MJN0	PCI domain-containing protein	0.00	G32628.3	0.00
A0A8S3VGU8	K1QX22	4-hydroxyphenylpyruvate dioxygenase	0.00	G23380.1	0.00
A0A8S3QVD1	A0A8W8MVB1	Small ribosomal subunit protein uS17	4.13×10^{-79}	G4085.2	3.22×10^{-79}
A0A6J8EWE8	K1R8C6	40S ribosomal protein S12	7.88×10^{-86}	G2853.3	6.16×10^{-86}
A0A2C9JRX0	A0A8W8I248	Small ribosomal subunit protein uS19	7.48×10^{-86}	G11948.8	5.84×10^{-86}
A0A8B6G0N3	Q4H450	Small ribosomal subunit protein uS13	3.05×10^{-103}	G24712.5	2.38×10^{-103}
V4AS57	A0A8W8LCY1	40S ribosomal protein S25	8.88×10^{-36}	G26985.2	6.93×10^{-36}
A0A8B6EK07	A0A8W8LH25	40S ribosomal protein S26	2.40×10^{-69}	G28190.5	1.87×10^{-69}
A0A8B6CVW2	A0A8W8MXY1	Signal peptide peptidase-like 3	9.71×10^{-94}	G35094.6	7.59×10^{-94}
A0A210PU23	Q4H451	Ribosomal protein S5	1.14×10^{-145}	G25192.2	8.87×10^{-146}
A0A3G1CJL1	A0A8W8NHW3	40S ribosomal protein S8	1.90×10^{-129}	G5375.11	1.48×10^{-129}
A0A8B6BQ82	A0A8W8HTG6	AMP-activated protein kinase glycogen-binding domain-containing protein	2.31×10^{-33}	G10958.1	1.80×10^{-33}
A0A8S3RZZ6	K1QWX2	60S acidic ribosomal protein P0	4.33×10^{-169}	G5077.17	3.38×10^{-169}
B6ZCB1	K1Q358	Large ribosomal subunit protein P2	1.66×10^{-33}	G23358.6	4.56×10^{-32}

A0A8S3V934	K1RDM2	60S ribosomal protein L18a	1.84×10^{-105}	G28274.6	1.43×10^{-105}
A0A077GYU2	A0A8W8N1A1	Large ribosomal subunit protein uL14	2.45×10^{-92}	G3885.13	1.91×10^{-92}
A0A077H3N0	A0A8W8M7W6	60S ribosomal protein L27	8.59×10^{-85}	G32202.11	6.71×10^{-85}
A0A077GZK7	A0A8W8LHY8	Large ribosomal subunit protein eL28	4.43×10^{-62}	G27586.1	3.46×10^{-62}
A0A8B6G8D8	A0A8W8L6C3	Large ribosomal subunit protein eL31	2.33×10^{-72}	G26894.6	1.82×10^{-72}
A0A8B6DEA0	A0A8W8NT75	Large ribosomal subunit protein eL34	1.17×10^{-66}	G732.3	9.10×10^{-67}
A0A8S3R313	A0A8W8I5V2	Large ribosomal subunit protein eL36	3.25×10^{-50}	G12711.1	2.54×10^{-50}
A0A8B6EI40	A0A8W8KDU6	Large ribosomal subunit protein eL6	1.80×10^{-116}	G23471.12	1.41×10^{-116}
A0A6J8A5K2	A0A8W8MW71	Large ribosomal subunit protein uL2	1.64×10^{-167}	G35349.1	1.28×10^{-167}
A0A8B6FK68	A0A8W8LFS8	Xylose isomerase	0.00	G2787.2	0.00
A0A8S3UF93	K1R5F2	14-3-3 protein epsilon	2.04×10^{-152}	G23866.50	1.59×10^{-152}

Table S4. Results of BLASTp analysis for DEPs identified in response to 2′-deoxyinosine (**2′**). The table lists homologous proteins from *Magallana gigas*. along with their corresponding accession numbers retrieved from the UniProt and Ensembl Metazoa databases.

Reference Sequence	UniProt			Ensembl	
	Accession number	Protein Name	e-Value	Accession number	e-Value
A0A8B6DSL8	K1RW85	Adenosylhomocysteinase	0.00	G22300.4	0.00
A0A8B6GV47	K1R0Y9	ADP/ATP translocase	0.00	G730.11	0.00
A0A8B6GU32	A0A8W8LM86	ATP synthase subunit gamma. mitochondrial	4.16×10^{-124}	G28739.12	3.25×10^{-124}
A0A8S3Q2E4	K1PQJ9	ATP synthase subunit delta. mitochondrial	2.28×10^{-45}	G269.2	1.78×10^{-45}
A0A6J8EBJ6	A0A8W8L8X3	Flotillin-1	0.00	G26928.23	0.00
A0A8B6GW86	A0A8W8J8Q2	C1q domain-containing protein	1.57×10^{-20}	G17851.1	1.23×10^{-20}
A0A8B6G4E0	A0A8W8KCX1	Calcium-transporting ATPase	0.00	G23365.9	0.00
A0A8B6FIN1	K1QY92	Chitin-binding type-2 domain-containing protein	5.43×10^{-49}	G22619.1	4.24×10^{-49}
A0A8B6C153	A0A8W8MWR9	Citrate synthase	0.00	G35409.40	0.00
A0A8B6ENY2	A0A8W8M8M9	CCHC-type domain-containing protein	2.64×10^{-28}	G31077.33	2.06×10^{-28}
A0A8S3R8F7	A0A8W8JPQ6	Arginine kinase	1.14×10^{-178}	G19705.1	8.89×10^{-179}
D9IWS1	A0A8W8N948	Elongation factor 1-alpha	0.00	G4818.20	0.00
A0A8B6EYD8	A0A8W8HXZ3	Elongation factor 2	0.00	G11483.21	0.00
A0A8S3QB23	K1R2L4	Histone H2B	2.28×10^{-66}	G22555.2	1.03×10^{-62}
A0A8B6DBF4	A0A8W8NN09	Metalloendopeptidase	8.22×10^{-113}	G6474.29	6.42×10^{-113}
A0A8B6G1S8	A0A8W8I1E8	Protein disulfide-isomerase	0.00	G11932.5	0.00
A0A8S3Q9N6	K1QZZ5	Stress-induced protein 1	1.00×10^{-33}	G17984.2	1.30×10^{-33}
A0A8B6D810	K1R401	Spectrin alpha chain	0.00	G25515.3	0.00
Q966V3	A0A8W8LCL2	Transgelin	0.00	G27652.16	0.00

A0A076FIR9	K1R7V7	Tubulin beta chain	0.00	G15286.68	0.00
A0A8B6CEW4	A0A8W8KET0	Uncharacterized protein	9.04x10^{-33}	G23190.43	7.06x10^{-33}
A0A8B6DC97	A0A8W8NN09	Metalloendopeptidase	6.29x10^{-128}	G6474.29	4.91x10^{-128}
A0A8B6BV40	K1PKC1	Collagen alpha-6(VI) chain	1.16x10^{-89}	G4540.1	7.57x10^{-80}
A0A8B6FD83	K1PDS7	Collagen alpha-2(I) chain	0.00	G5252.2	5.45x10^{-67}
A0A8B6G747	A0A8W8KPZ6	Fibrillar collagen NC1 domain-containing protein	0.00	G24458.2	0.00
A0A8S3QII9	K1Q7M2	Cathepsin L	0.00	G9706.23	0.00
A0A8B6HEV7	A0A8W8NFU0	LIM zinc-binding domain-containing protein	3.22x10^{-96}	G5979.9	2.51x10^{-96}
A0A8B6F031	K1QKV1	Cytochrome b-c1 complex subunit 6. mitochondrial	4.13x10^{-12}	G2226.1	3.23x10^{-12}
A0A8B6D685	A0A8W8I7C9	Dihydrolipoyllysine-residue succinyltransferase component of 2-oxoglutarate dehydrogenase complex. mitochondrial	1.36x10^{-159}	G12941.5	1.06x10^{-159}
A0A409V740	---NA---	---NA---	---NA---	---NA---	---NA---
A0A3L5TQZ0	A0A8W8HZA6	C-type lectin domain-containing protein	3.96x10^{-44}	G11780.1	3.09x10^{-44}
A0A6J8ECB1	K1PTI6	Glucose-6-phosphate isomerase	5.75x10^{-177}	G20982.5	1.04x10^{-176}
A0A8B6DQP9	K1QRQ2	glutamate dehydrogenase	0.00	G3271.17	0.00
A0A8B6H497	A0A8W8MQI2	Granulins domain-containing protein	0.00	G34862.1	0.00
A0A411G5T5	A0A8W8KZI1	Ig-like domain-containing protein	2.17x10^{-10}	G25938.1	1.70x10^{-10}
K1Q324	K1Q324	Heterogeneous nuclear ribonucleoprotein K	0.00	G10728.1	1.06x10^{-101}
A0A0B7AUT8	A0A8W8NPB5	Histone H2A	1.79x10^{-85}	G661.4	1.40x10^{-85}
A0A8B6DFJ0	A0A8W8M908	RRM domain-containing protein	0.00	G31955.2	0.00
A0A8B6BTE6	A0A8W8N7H9	Large ribosomal subunit protein P1	1.62x10^{-32}	G5649.7	1.26x10^{-32}
A0A8S3Q6R0	K1RFA3	Lamin Dm0	0.00	G11465.2	0.00
A0A8B6C2Y2	A0A8W8K8K1	Malate dehydrogenase. mitochondrial	2.45x10^{-162}	G22856.16	1.91x10^{-162}
A0A8B6FW38	A0A8W8J1A5	Stress-70 protein. mitochondrial	0.00	G16610.7	0.00
A0A6J8APW2	A0A8W8IDE4	Nidogen-1	0.00	G13651.1	0.00
A0A8S3ULS9	K1RHA5	Nucleoside diphosphate kinase	3.22x10^{-88}	G27377.8	1.12x10^{-72}
A0A8B6D9K2	---NA---	---NA---	---NA---	---NA---	---NA---
A0A6J8DKH3	A0A8W8IB78	Peptidyl-prolyl cis-trans isomerase	9.28x10^{-140}	G13223.1	7.25x10^{-140}
A0A8S3TQI9	A0A8W8MDY5	EF-hand domain-containing protein	2.18x10^{-82}	G32504.8	1.71x10^{-82}
A0A6J8EGE4	A0A8W8JWQ7	TNF receptor-associated factor 4	0.00	G21164.1	0.00
A0A8S3QSQ2	K1PD36	Ubiquitin-ribosomal protein eL40 fusion protein	2.89x10^{-92}	G26976.2	2.26x10^{-92}
A0A3L5TR65	A0A8W8HUR9	Protocadherin Fat 4	1.68x10^{-16}	G11053.1	1.31x10^{-16}

A0A8B6E104	K1PSR5	Fatty acid-binding protein. heart	3.26x10^{-34}	G21524.4	2.90x10^{-34}
A0A8B6GUE8	---NA---	---NA---	---NA---	---NA---	---NA---
A0A8B6CB58	A0A8W8JRD3	Apextrin C-terminal domain-containing protein	2.36x10^{-08}	G20570.1	1.84x10^{-08}
A0A8B6DFD7	A0A8W8HVW8	Papilin	3.74x10^{-21}	G11186.1	2.92x10^{-21}
A0A8B6BNI7	---NA---	---NA---	---NA---	---NA---	---NA---
A0A8B6DPQ0	K1R459	SCO-spondin	0.00	G35504.1	1.30x10^{-133}
A0A8B6FV73	A0A8W8J7T4	LIM zinc-binding domain-containing protein	0.00	G17454.23	0.00
A0A8B6CCQ9	K1PRV7	Profilin	6.77x10^{-06}	G16496.5	5.29x10^{-06}
A0A8B6H3D0	A0A8W8KJT0	MSP domain-containing protein	2.61x10^{-105}	G23892.18	2.04x10^{-105}
A0A8B6GXU1	A0A8W8JME6	Vitelline membrane outer layer protein 1	2.26x10^{-48}	G19499.2	1.76x10^{-48}
A0A8B6EK07	A0A8W8LH25	40S ribosomal protein S26	2.40x10^{-69}	G28190.5	1.87x10^{-69}
A0A3G1CJL1	A0A8W8NHW3	40S ribosomal protein S8	1.90x10^{-129}	G5375.11	1.48x10^{-129}
A0A8S3QVR3	A0A8W8MQU1	Small ribosomal subunit protein uS2	1.03x10^{-14}3	G3387.29	8.05x10^{-144}
A0A6J8B1B1	A0A8W8NW87	ATP-dependent 6-phosphofructokinase	0.00	G7024.1	0.00
A0A8S3RZZ6	K1QWX2	60S acidic ribosomal protein P0	4.33x10^{-169}	G5077.17	3.38x10^{-169}
A0A6J8DYJ1	A0A8W8N1Z8	Large ribosomal subunit protein uL11	1.10x10-94	G4972.15	8.61x10^{-95}
A0A8S3V934	K1RDM2	60S ribosomal protein L18a	1.84x10-105	G28274.6	1.43x10^{-105}
A0A077H0N7	A0A8W8L109	60S ribosomal protein L7a	1.38x10^{-155}	G25420.3	1.08x10^{-155}

Table S3. Functional analysis of DEPs identified in response to hypoxanthine arabinoside (**1′**). Gene Ontology (GO) enrichment was performed using the g:GOSt tool in g:Profiler. The resulting GO terms are categorized into Molecular Functions (MF). Biological Processes (BP). and Cellular Components (CC). Both GO term names. and their corresponding IDs are listed.

**Category**	**GO Name**	**GO ID**	**Adjusted *p*-value**
GO:MF	structural molecule activity	GO:0005198	2.20x10^{-31}
GO:MF	structural constituent of ribosome	GO:0003735	3.90x10^{-25}
GO:MF	actin binding	GO:0003779	2.75x10^{-09}
GO:MF	cytoskeletal protein binding	GO:0008092	4.46x10^{-07}
GO:MF	intramolecular oxidoreductase activity	GO:0016860	5.06x10^{-05}
GO:MF	protein-containing complex binding	GO:0044877	6.73x10^{-05}
GO:MF	isomerase activity	GO:0016853	6.73x10^{-05}
GO:MF	calcium ion binding	GO:0005509	1.69x10^{-04}
GO:MF	actin filament binding	GO:0051015	2.00x10^{-04}
GO:MF	ribonucleoside triphosphate phosphatase activity	GO:0017111	5.89x10^{-04}

GO:MF	intramolecular oxidoreductase activity. transposing S-S bonds	GO:0016864	$6.05x10^{-04}$
GO:MF	proton-transporting ATP synthase activity. rotational mechanism	GO:0046933	$6.05x10^{-04}$
GO:MF	protein disulfide isomerase activity	GO:0003756	$6.05x10^{-04}$
GO:MF	oxidoreductase activity. acting on the aldehyde or oxo group of donors	GO:0016903	$6.97x10^{-04}$
GO:MF	ATPase-coupled monoatomic cation transmembrane transporter activity	GO:0019829	$1.02x10^{-03}$
GO:MF	pyrophosphatase activity	GO:0016462	$1.06x10^{-03}$
GO:MF	hydrolase activity. acting on acid anhydrides. in phosphorus-containing anhydrides	GO:0016818	$1.32x10^{-03}$
GO:MF	hydrolase activity. acting on acid anhydrides	GO:0016817	$1.32x10^{-03}$
GO:MF	unfolded protein binding	GO:0051082	$1.37x10^{-03}$
GO:MF	proton transmembrane transporter activity	GO:0015078	$2.90x10^{-03}$
GO:MF	peroxiredoxin activity	GO:0051920	$4.79x10^{-03}$
GO:MF	oxidoreductase activity	GO:0016491	0.01
GO:MF	heterocyclic compound binding	GO:1901363	0.01
GO:MF	RNA binding	GO:0003723	0.01
GO:MF	alcohol dehydrogenase (NAD+) activity	GO:0004022	0.01
GO:MF	fructose-bisphosphate aldolase activity	GO:0004332	0.01
GO:MF	alcohol dehydrogenase [NAD(P)+] activity	GO:0018455	0.01
GO:MF	L-malate dehydrogenase (NAD+) activity	GO:0030060	0.01
GO:MF	translation elongation factor activity	GO:0003746	0.01
GO:MF	anion binding	GO:0043168	0.01
GO:MF	active monoatomic ion transmembrane transporter activity	GO:0022853	0.01
GO:MF	extracellular matrix structural constituent	GO:0005201	0.01
GO:MF	P-type ion transporter activity	GO:0015662	0.01
GO:MF	intramolecular oxidoreductase activity. interconverting aldoses and ketoses	GO:0016861	0.01
GO:MF	oxidoreductase activity. acting on the aldehyde or oxo group of donors. NAD or NADP as acceptor	GO:0016620	0.01
GO:MF	phosphotransferase activity. phosphate group as acceptor	GO:0016776	0.01
GO:MF	P-type transmembrane transporter activity	GO:0140358	0.02
GO:MF	P-type calcium transporter activity	GO:0005388	0.02
GO:MF	aldehyde-lyase activity	GO:0016832	0.02
GO:MF	oxidoreductase activity. acting on the CH-OH group of donors. NAD or NADP as acceptor	GO:0016616	0.02
GO:MF	GTPase activity	GO:0003924	0.02
GO:MF	vitamin binding	GO:0019842	0.02
GO:MF	pyridoxal phosphate binding	GO:0030170	0.02
GO:MF	vitamin B6 binding	GO:0070279	0.02
GO:MF	ATP hydrolysis activity	GO:0016887	0.02
GO:MF	nucleotide binding	GO:0000166	0.02
GO:MF	intramolecular phosphotransferase activity	GO:0016868	0.02
GO:MF	nucleoside phosphate binding	GO:1901265	0.02
GO:MF	cAMP-dependent protein kinase regulator activity	GO:0008603	0.03
GO:MF	ribosome binding	GO:0043022	0.03
GO:MF	peptidyl-prolyl cis-trans isomerase activity	GO:0003755	0.03
GO:MF	cis-trans isomerase activity	GO:0016859	0.04
GO:MF	oxidoreductase activity. acting on the CH-NH group of donors. NAD or NADP as acceptor	GO:0016646	0.04
GO:MF	malate dehydrogenase activity	GO:0016615	0.04
GO:MF	cAMP binding	GO:0030552	0.04
GO:MF	oxidoreductase activity. acting on the aldehyde or oxo group of donors. disulfide as acceptor	GO:0016624	0.04

GO:MF	antioxidant activity	GO:0016209	0.04
GO:MF	nucleobase-containing compound kinase activity	GO:0019205	0.05
GO:MF	phospholipid transporter activity	GO:0005548	0.05
GO:MF	purine ribonucleotide binding	GO:0032555	0.05
GO:MF	purine nucleotide binding	GO:0017076	0.05
GO:BP	translation	GO:0006412	$2.40x10^{-17}$
GO:BP	purine ribonucleoside triphosphate metabolic process	GO:0009205	$6.85x10^{-15}$
GO:BP	purine nucleoside triphosphate metabolic process	GO:0009144	$6.85x10^{-15}$
GO:BP	ribonucleoside triphosphate metabolic process	GO:0009199	$1.21x10^{-14}$
GO:BP	generation of precursor metabolites and energy	GO:0006091	$4.02x10^{-14}$
GO:BP	ATP metabolic process	GO:0046034	$6.13x10^{-14}$
GO:BP	nucleoside triphosphate metabolic process	GO:0009141	$1.02x10^{-13}$
GO:BP	nucleoside phosphate metabolic process	GO:0006753	$9.66x10^{-10}$
GO:BP	purine-containing compound metabolic process	GO:0072521	$1.79x10^{-09}$
GO:BP	purine nucleotide metabolic process	GO:0006163	$3.02x10^{-09}$
GO:BP	small molecule metabolic process	GO:0044281	$3.31x10^{-09}$
GO:BP	nucleotide metabolic process	GO:0009117	$4.35x10^{-09}$
GO:BP	nucleobase-containing small molecule metabolic process	GO:0055086	$4.35x10^{-09}$
GO:BP	purine ribonucleotide metabolic process	GO:0009150	$1.84x10^{-08}$
GO:BP	ribonucleoside diphosphate catabolic process	GO:0009191	$3.69x10^{-08}$
GO:BP	ADP catabolic process	GO:0046032	$3.69x10^{-08}$
GO:BP	ribonucleotide metabolic process	GO:0009259	$3.69x10^{-08}$
GO:BP	purine nucleoside diphosphate catabolic process	GO:0009137	$3.69x10^{-08}$
GO:BP	ribose phosphate metabolic process	GO:0019693	$3.69x10^{-08}$
GO:BP	pyruvate metabolic process	GO:0006090	$3.69x10^{-08}$
GO:BP	glycolytic process	GO:0006096	$3.69x10^{-08}$
GO:BP	nucleoside diphosphate catabolic process	GO:0009134	$3.69x10^{-08}$
GO:BP	purine ribonucleoside diphosphate catabolic process	GO:0009181	$3.69x10^{-08}$
GO:BP	purine ribonucleotide catabolic process	GO:0009154	$4.88x10^{-08}$
GO:BP	ribonucleotide catabolic process	GO:0009261	$4.88x10^{-08}$
GO:BP	ADP metabolic process	GO:0046031	$4.88x10^{-08}$
GO:BP	pyridine nucleotide catabolic process	GO:0019364	$4.88x10^{-08}$
GO:BP	pyridine-containing compound catabolic process	GO:0072526	$4.88x10^{-08}$
GO:BP	carboxylic acid metabolic process	GO:0019752	$6.37x10^{-08}$
GO:BP	purine nucleotide catabolic process	GO:0006195	$7.12x10^{-08}$
GO:BP	oxoacid metabolic process	GO:0043436	$9.36x10^{-08}$
GO:BP	organic acid metabolic process	GO:0006082	$1.31x10^{-07}$
GO:BP	purine nucleoside diphosphate metabolic process	GO:0009135	$1.32x10^{-07}$
GO:BP	tricarboxylic acid cycle	GO:0006099	$1.32x10^{-07}$
GO:BP	purine ribonucleoside diphosphate metabolic process	GO:0009179	$1.32x10^{-07}$
GO:BP	energy derivation by oxidation of organic compounds	GO:0015980	$1.32x10^{-07}$
GO:BP	ribonucleoside diphosphate metabolic process	GO:0009185	$1.32x10^{-07}$
GO:BP	nicotinamide nucleotide metabolic process	GO:0046496	$2.13x10^{-07}$
GO:BP	nucleoside diphosphate metabolic process	GO:0009132	$2.59x10^{-07}$
GO:BP	aerobic respiration	GO:0009060	$2.59x10^{-07}$
GO:BP	pyridine nucleotide metabolic process	GO:0019362	$3.20x10^{-07}$
GO:BP	purine-containing compound catabolic process	GO:0072523	$4.88x10^{-07}$
GO:BP	purine ribonucleoside triphosphate biosynthetic process	GO:0009206	$1.11x10^{-06}$
GO:BP	purine nucleoside triphosphate biosynthetic process	GO:0009145	$1.11x10^{-06}$
GO:BP	organophosphate metabolic process	GO:0019637	$1.29x10^{-06}$
GO:BP	cellular respiration	GO:0045333	$1.29x10^{-06}$

GO:BP	pyridine-containing compound metabolic process	GO:0072524	$1.54x10^{-06}$
GO:BP	ribonucleoside triphosphate biosynthetic process	GO:0009201	$2.06x10^{-06}$
GO:BP	nucleoside phosphate catabolic process	GO:1901292	$2.06x10^{-06}$
GO:BP	nucleoside triphosphate biosynthetic process	GO:0009142	$2.75x10^{-06}$
GO:BP	nucleotide catabolic process	GO:0009166	$4.89x10^{-06}$
GO:BP	organophosphate catabolic process	GO:0046434	$7.97x10^{-06}$
GO:BP	proton motive force-driven ATP synthesis	GO:0015986	$1.23x10^{-05}$
GO:BP	ATP biosynthetic process	GO:0006754	$1.89x10^{-05}$
GO:BP	cilium movement involved in cell motility	GO:0060294	$2.65x10^{-05}$
GO:BP	carbohydrate catabolic process	GO:0016052	$3.47x10^{-05}$
GO:BP	cilium or flagellum-dependent cell motility	GO:0001539	$4.51x10^{-05}$
GO:BP	cilium-dependent cell motility	GO:0060285	$4.51x10^{-05}$
GO:BP	monocarboxylic acid metabolic process	GO:0032787	$6.54x10^{-05}$
GO:BP	cytoskeleton organization	GO:0007010	$1.01x10^{-04}$
GO:BP	biosynthetic process	GO:0009058	$1.65x10^{-04}$
GO:BP	protein metabolic process	GO:0019538	$2.19x10^{-04}$
GO:BP	actin cytoskeleton organization	GO:0030036	$2.45x10^{-04}$
GO:BP	actin filament-based process	GO:0030029	$2.70x10^{-04}$
GO:BP	cilium movement	GO:0003341	$6.46x10^{-04}$
GO:BP	actin filament organization	GO:0007015	$1.01x10^{-03}$
GO:BP	organic acid catabolic process	GO:0016054	$1.08x10^{-03}$
GO:BP	carboxylic acid catabolic process	GO:0046395	$1.08x10^{-03}$
GO:BP	regulation of protein-containing complex disassembly	GO:0043244	$1.08x10^{-03}$
GO:BP	translational elongation	GO:0006414	$1.18x10^{-03}$
GO:BP	supramolecular fiber organization	GO:0097435	$1.38x10^{-03}$
GO:BP	carbohydrate derivative catabolic process	GO:1901136	$1.55x10^{-03}$
GO:BP	nucleobase-containing compound catabolic process	GO:0034655	$1.65x10^{-03}$
GO:BP	cell motility	GO:0048870	$2.01x10^{-03}$
GO:BP	primary metabolic process	GO:0044238	$2.63x10^{-03}$
GO:BP	glucose metabolic process	GO:0006006	$2.81x10^{-03}$
GO:BP	protein-containing complex disassembly	GO:0032984	$3.55x10^{-03}$
GO:BP	actin filament depolymerization	GO:0030042	$3.57x10^{-03}$
GO:BP	protein depolymerization	GO:0051261	$3.57x10^{-03}$
GO:BP	organelle organization	GO:0006996	$3.57x10^{-03}$
GO:BP	cellular catabolic process	GO:0044248	$3.62x10^{-03}$
GO:BP	small molecule catabolic process	GO:0044282	$3.62x10^{-03}$
GO:BP	lysine catabolic process	GO:0006554	$4.80x10^{-03}$
GO:BP	gene expression	GO:0010467	$4.80x10^{-03}$
GO:BP	L-lysine catabolic process to acetyl-CoA via saccharopine	GO:0033512	$4.80x10^{-03}$
GO:BP	L-lysine catabolic process to acetyl-CoA	GO:0019474	$4.80x10^{-03}$
GO:BP	L-lysine catabolic process	GO:0019477	$4.80x10^{-03}$
GO:BP	L-lysine metabolic process	GO:0046440	$4.80x10^{-03}$
GO:BP	nucleotide biosynthetic process	GO:0009165	$4.86x10^{-03}$
GO:BP	proteinogenic amino acid catabolic process	GO:0170040	0.01
GO:BP	carbohydrate biosynthetic process	GO:0016051	0.01
GO:BP	nucleoside phosphate biosynthetic process	GO:1901293	0.01
GO:BP	carbohydrate derivative metabolic process	GO:1901135	0.01
GO:BP	cellular component disassembly	GO:0022411	0.01
GO:BP	purine-containing compound biosynthetic process	GO:0072522	0.01
GO:BP	carbohydrate metabolic process	GO:0005975	0.01
GO:BP	L-amino acid catabolic process	GO:0170035	0.01

GO:BP	protein folding	GO:0006457	0.01
GO:BP	metabolic process	GO:0008152	0.01
GO:BP	purine nucleotide biosynthetic process	GO:0006164	0.01
GO:BP	catabolic process	GO:0009056	0.01
GO:BP	purine ribonucleotide biosynthetic process	GO:0009152	0.01
GO:BP	hexose biosynthetic process	GO:0019319	0.01
GO:BP	monosaccharide biosynthetic process	GO:0046364	0.01
GO:BP	actin filament capping	GO:0051693	0.01
GO:BP	negative regulation of protein depolymerization	GO:1901880	0.01
GO:BP	regulation of protein depolymerization	GO:1901879	0.01
GO:BP	gluconeogenesis	GO:0006094	0.01
GO:BP	regulation of actin filament depolymerization	GO:0030834	0.01
GO:BP	negative regulation of protein-containing complex disassembly	GO:0043242	0.01
GO:BP	negative regulation of actin filament depolymerization	GO:0030835	0.01
GO:BP	ribonucleotide biosynthetic process	GO:0009260	0.01
GO:BP	ribose phosphate biosynthetic process	GO:0046390	0.01
GO:BP	proton transmembrane transport	GO:1902600	0.01
GO:BP	regulation of protein metabolic process	GO:0051246	0.02
GO:BP	macromolecule biosynthetic process	GO:0009059	0.02
GO:BP	acetyl-CoA metabolic process	GO:0006084	0.02
GO:BP	one-carbon metabolic process	GO:0006730	0.02
GO:BP	aspartate family amino acid catabolic process	GO:0009068	0.02
GO:BP	actin polymerization or depolymerization	GO:0008154	0.02
GO:BP	negative regulation of actin filament polymerization	GO:0030837	0.03
GO:BP	fatty acid beta-oxidation	GO:0006635	0.03
GO:BP	negative regulation of protein polymerization	GO:0032272	0.03
GO:BP	negative regulation of protein-containing complex assembly	GO:0031333	0.03
GO:BP	negative regulation of supramolecular fiber organization	GO:1902904	0.03
GO:BP	lysine metabolic process	GO:0006553	0.03
GO:BP	cell adhesion	GO:0007155	0.03
GO:BP	microtubule-based process	GO:0007017	0.04
GO:BP	fatty acid oxidation	GO:0019395	0.04
GO:BP	alpha-amino acid catabolic process	GO:1901606	0.04
GO:BP	negative regulation of cytoskeleton organization	GO:0051494	0.04
GO:BP	cellular component organization or biogenesis	GO:0071840	0.04
GO:BP	proteinogenic amino acid metabolic process	GO:0170039	0.04
GO:BP	tyrosine catabolic process	GO:0006572	0.04
GO:BP	regulation of protein catabolic process	GO:0042176	0.04
GO:BP	L-amino acid metabolic process	GO:0170033	0.04
GO:BP	protein polymerization	GO:0051258	0.04
GO:BP	fatty acid catabolic process	GO:0009062	0.05
GO:BP	organophosphate biosynthetic process	GO:0090407	0.05
GO:BP	plasma membrane bounded cell projection organization	GO:0120036	0.05
GO:CC	ribosome	GO:0005840	$8.34x10^{-26}$
GO:CC	ribonucleoprotein complex	GO:1990904	$1.09x10^{-18}$
GO:CC	protein-containing complex	GO:0032991	$2.29x10^{-16}$
GO:CC	membraneless organelle	GO:0043228	$4.66x10^{-15}$
GO:CC	intracellular membraneless organelle	GO:0043232	$4.66x10^{-15}$
GO:CC	intracellular anatomical structure	GO:0005622	$4.52x10^{-10}$
GO:CC	cytosolic ribosome	GO:0022626	$5.70x10^{-08}$
GO:CC	ribosomal subunit	GO:0044391	$6.15x10^{-08}$

GO:CC	organelle	GO:0043226	7.97x10^{-08}
GO:CC	cytosol	GO:0005829	1.21x10^{-06}
GO:CC	cytoplasm	GO:0005737	1.21x10^{-06}
GO:CC	intracellular organelle	GO:0043229	1.21x10^{-06}
GO:CC	proton-transporting ATP synthase complex. catalytic core F(1)	GO:0045261	9.98x10^{-06}
GO:CC	proton-transporting two-sector ATPase complex. catalytic domain	GO:0033178	1.02x10^{-05}
GO:CC	large ribosomal subunit	GO:0015934	1.49x10^{-05}
GO:CC	proton-transporting ATP synthase complex	GO:0045259	5.03x10^{-05}
GO:CC	proton-transporting two-sector ATPase complex	GO:0016469	8.71x10^{-05}
GO:CC	extracellular matrix	GO:0031012	1.44x10^{-04}
GO:CC	external encapsulating structure	GO:0030312	1.44x10^{-04}
GO:CC	respiratory chain complex	GO:0098803	2.91x10^{-04}
GO:CC	endoplasmic reticulum lumen	GO:0005788	4.07x10^{-04}
GO:CC	basement membrane	GO:0005604	1.08x10^{-03}
GO:CC	cytoskeleton	GO:0005856	1.23x10^{-03}
GO:CC	collagen-containing extracellular matrix	GO:0062023	2.24x10^{-03}
GO:CC	cytosolic large ribosomal subunit	GO:0022625	3.52x10^{-03}
GO:CC	small ribosomal subunit	GO:0015935	3.78x10^{-03}
GO:CC	membrane protein complex	GO:0098796	3.89x10^{-03}
GO:CC	plasma membrane bounded cell projection cytoplasm	GO:0032838	0.01
GO:CC	ciliary plasm	GO:0097014	0.01
GO:CC	axoneme	GO:0005930	0.01
GO:CC	cytoplasmic region	GO:0099568	0.01
GO:CC	mitochondrial inner membrane	GO:0005743	0.01
GO:CC	organelle inner membrane	GO:0019866	0.01
GO:CC	cAMP-dependent protein kinase complex	GO:0005952	0.01
GO:CC	intermediate filament	GO:0005882	0.01
GO:CC	radial spoke	GO:0001534	0.01
GO:CC	intermediate filament cytoskeleton	GO:0045111	0.01
GO:CC	actin cytoskeleton	GO:0015629	0.02
GO:CC	motile cilium	GO:0031514	0.02
GO:CC	collagen trimer	GO:0005581	0.02
GO:CC	oligosaccharyltransferase complex	GO:0008250	0.02
GO:CC	Golgi-associated vesicle membrane	GO:0030660	0.02
GO:CC	Golgi-associated vesicle	GO:0005798	0.02
GO:CC	catalytic complex	GO:1902494	0.02
GO:CC	polymeric cytoskeletal fiber	GO:0099513	0.03
GO:CC	COPI vesicle coat	GO:0030126	0.03
GO:CC	cation channel complex	GO:0034703	0.03
GO:CC	oxidoreductase complex	GO:1990204	0.04
GO:CC	mitochondrial envelope	GO:0005740	0.04
GO:CC	supramolecular fiber	GO:0099512	0.04
GO:CC	coated membrane	GO:0048475	0.04
GO:CC	supramolecular polymer	GO:0099081	0.04
GO:CC	cilium	GO:0005929	0.04
GO:CC	vesicle coat	GO:0030120	0.04
GO:CC	membrane coat	GO:0030117	0.04
GO:CC	plasma membrane bounded cell projection	GO:0120025	0.05

Table S4. Functional analysis of DEPs identified in response to 2′-deoxyinosine (**2′**). Gene Ontology (GO) enrichment was performed using the g:GOSt tool in g:Profiler. The resulting GO terms are

https://doi.org/10.3390/xxxxx

categorized into Molecular Functions (MF). Biological Processes (BP). and Cellular Components (CC). Both GO term names. and their corresponding IDs are listed.

Category	GO Name	GO ID	Adjusted *p*-value
GO:MF	structural molecule activity	GO:0005198	$7.30x10^{-10}$
GO:MF	structural constituent of ribosome	GO:0003735	$2.77x10^{-05}$
GO:MF	proton-transporting ATP synthase activity. rotational mechanism	GO:0046933	0.01
GO:MF	extracellular matrix structural constituent	GO:0005201	0.01
GO:MF	structural constituent of chromatin	GO:0030527	0.04
GO:MF	adenosylhomocysteinase activity	GO:0004013	0.04
GO:MF	citrate (Si)-synthase activity	GO:0004108	0.04
GO:MF	dihydrolipoyllysine-residue succinyltransferase activity	GO:0004149	0.04
GO:MF	glutamate dehydrogenase (NADP+) activity	GO:0004354	0.04
GO:MF	S-succinyltransferase activity	GO:0016751	0.04
GO:MF	intramolecular oxidoreductase activity	GO:0016860	0.04
GO:MF	hydrolase activity. acting on carbon-sulfur bonds	GO:0046508	0.04
GO:MF	glutamate dehydrogenase (NAD+) activity	GO:0004352	0.04
GO:MF	citrate synthase activity	GO:0036440	0.04
GO:MF	translation elongation factor activity	GO:0003746	0.04
GO:MF	L-malate dehydrogenase (NAD+) activity	GO:0030060	0.05
GO:MF	succinyltransferase activity	GO:0016748	0.05
GO:MF	proton channel activity	GO:0015252	0.05
GO:MF	glutamate dehydrogenase [NAD(P)+] activity	GO:0004353	0.05
GO:MF	glucose-6-phosphate isomerase activity	GO:0004347	0.05
GO:MF	6-phosphofructokinase activity	GO:0003872	$4.82x10^{-02}$
GO:BP	purine nucleoside triphosphate metabolic process	GO:0009144	$7.56x10^{-05}$
GO:BP	ribonucleoside triphosphate metabolic process	GO:0009199	$7.56x10^{-05}$
GO:BP	purine ribonucleoside triphosphate metabolic process	GO:0009205	$7.56x10^{-05}$
GO:BP	translation	GO:0006412	$9.53x10^{-05}$
GO:BP	nucleoside triphosphate metabolic process	GO:0009141	$9.53x10^{-05}$
GO:BP	generation of precursor metabolites and energy	GO:0006091	$1.11x10^{-04}$
GO:BP	ATP metabolic process	GO:0046034	$4.59x10^{-04}$
GO:BP	aerobic respiration	GO:0009060	$7.70x10^{-04}$
GO:BP	cellular respiration	GO:0045333	$1.15x10^{-03}$
GO:BP	purine-containing compound metabolic process	GO:0072521	$1.15x10^{-03}$
GO:BP	purine ribonucleotide metabolic process	GO:0009150	$1.22x10^{-03}$
GO:BP	ribonucleotide metabolic process	GO:0009259	$1.37x10^{-03}$
GO:BP	ribose phosphate metabolic process	GO:0019693	$1.37x10^{-03}$
GO:BP	energy derivation by oxidation of organic compounds	GO:0015980	$1.48x10^{-03}$
GO:BP	purine ribonucleoside triphosphate biosynthetic process	GO:0009206	$1.48x10^{-03}$
GO:BP	purine nucleoside triphosphate biosynthetic process	GO:0009145	$1.48x10^{-03}$
GO:BP	tricarboxylic acid cycle	GO:0006099	$1.63x10^{-03}$
GO:BP	ribonucleoside triphosphate biosynthetic process	GO:0009201	$1.63x10^{-03}$
GO:BP	nucleoside triphosphate biosynthetic process	GO:0009142	$1.74x10^{-03}$
GO:BP	purine nucleotide metabolic process	GO:0006163	$1.90x10^{-03}$
GO:BP	nucleoside phosphate metabolic process	GO:0006753	$2.55x10^{-03}$
GO:BP	small molecule metabolic process	GO:0044281	$3.65x10^{-03}$
GO:BP	nucleobase-containing small molecule metabolic process	GO:0055086	$3.65x10^{-03}$
GO:BP	organophosphate metabolic process	GO:0019637	$3.85x10^{-03}$
GO:BP	nucleotide metabolic process	GO:0009117	$6.48x10^{-03}$

GO:BP	proton motive force-driven ATP synthesis	GO:0015986	0.01
GO:BP	ATP biosynthetic process	GO:0006754	0.02
GO:BP	ADP metabolic process	GO:0046031	0.02
GO:BP	purine ribonucleotide catabolic process	GO:0009154	0.02
GO:BP	ribonucleotide catabolic process	GO:0009261	0.02
GO:BP	nucleoside diphosphate catabolic process	GO:0009134	0.02
GO:BP	purine nucleoside diphosphate catabolic process	GO:0009137	0.02
GO:BP	glycolytic process	GO:0006096	0.02
GO:BP	ribonucleoside diphosphate catabolic process	GO:0009191	0.02
GO:BP	purine ribonucleoside diphosphate catabolic process	GO:0009181	0.02
GO:BP	pyridine nucleotide catabolic process	GO:0019364	0.02
GO:BP	pyridine-containing compound catabolic process	GO:0072526	0.02
GO:BP	ADP catabolic process	GO:0046032	0.02
GO:BP	organic acid metabolic process	GO:0006082	0.02
GO:BP	purine ribonucleotide biosynthetic process	GO:0009152	0.02
GO:BP	ribonucleoside diphosphate metabolic process	GO:0009185	0.02
GO:BP	purine nucleoside diphosphate metabolic process	GO:0009135	0.02
GO:BP	carboxylic acid metabolic process	GO:0019752	0.02
GO:BP	oxoacid metabolic process	GO:0043436	0.02
GO:BP	purine nucleotide catabolic process	GO:0006195	0.02
GO:BP	ribosomal small subunit assembly	GO:0000028	0.02
GO:BP	purine ribonucleoside diphosphate metabolic process	GO:0009179	0.02
GO:BP	ribonucleotide biosynthetic process	GO:0009260	0.03
GO:BP	nucleoside diphosphate metabolic process	GO:0009132	0.03
GO:BP	ribose phosphate biosynthetic process	GO:0046390	0.03
GO:BP	pyruvate metabolic process	GO:0006090	0.03
GO:BP	purine-containing compound catabolic process	GO:0072523	0.03
GO:BP	purine nucleotide biosynthetic process	GO:0006164	0.03
GO:BP	translational elongation	GO:0006414	0.03
GO:BP	ribosome biogenesis	GO:0042254	0.03
GO:BP	glycolytic process through fructose-6-phosphate	GO:0061615	0.03
GO:BP	L-lysine catabolic process	GO:0019477	0.03
GO:BP	L-lysine metabolic process	GO:0046440	0.03
GO:BP	L-lysine catabolic process to acetyl-CoA via saccharopine	GO:0033512	0.03
GO:BP	fructose 6-phosphate metabolic process	GO:0006002	0.03
GO:BP	citrate metabolic process	GO:0006101	0.03
GO:BP	L-lysine catabolic process to acetyl-CoA	GO:0019474	0.03
GO:BP	lysine catabolic process	GO:0006554	0.03
GO:BP	nucleotide catabolic process	GO:0009166	0.04
GO:BP	purine-containing compound biosynthetic process	GO:0072522	0.04
GO:BP	biosynthetic process	GO:0009058	0.04
GO:BP	phosphagen biosynthetic process	GO:0042396	0.04
GO:BP	phosphocreatine metabolic process	GO:0006603	0.04
GO:BP	phosphagen metabolic process	GO:0006599	0.04
GO:BP	phosphocreatine biosynthetic process	GO:0046314	0.04
GO:BP	organophosphate biosynthetic process	GO:0090407	0.05
GO:BP	nucleoside phosphate catabolic process	GO:1901292	0.05
GO:BP	nicotinamide nucleotide metabolic process	GO:0046496	0.05
GO:BP	carbohydrate catabolic process	GO:0016052	0.05
GO:BP	mitochondrial ADP transmembrane transport	GO:0140021	0.05
GO:BP	purine-containing compound transmembrane transport	GO:0072530	0.05

GO:BP	tricarboxylic acid metabolic process	GO:0072350	0.05
GO:BP	adenine nucleotide transport	GO:0051503	0.05
GO:BP	mitochondrial ATP transmembrane transport	GO:1990544	0.05
GO:BP	nucleotide transmembrane transport	GO:1901679	0.05
GO:BP	aspartate family amino acid catabolic process	GO:0009068	0.05
GO:BP	nucleotide biosynthetic process	GO:0009165	0.05
GO:BP	purine nucleotide transport	GO:0015865	0.05
GO:BP	ribonucleoprotein complex biogenesis	GO:0022613	0.05
GO:BP	ADP transport	GO:0015866	0.05
GO:BP	ATP transport	GO:0015867	0.05
GO:BP	purine ribonucleotide transport	GO:0015868	0.05
GO:BP	pyridine nucleotide metabolic process	GO:0019362	0.05
GO:BP	organophosphate catabolic process	GO:0046434	0.05
GO:CC	ribosome	GO:0005840	$1.91x10^{-06}$
GO:CC	ribonucleoprotein complex	GO:1990904	$7.31x10^{-06}$
GO:CC	intracellular membraneless organelle	GO:0043232	$1.73x10^{-04}$
GO:CC	membraneless organelle	GO:0043228	$1.73x10^{-04}$
GO:CC	protein-containing complex	GO:0032991	$3.32x10^{-04}$
GO:CC	cytosolic ribosome	GO:0022626	$4.50x10^{-04}$
GO:CC	proton-transporting ATP synthase complex. catalytic core F(1)	GO:0045261	$1.28x10^{-03}$
GO:CC	proton-transporting ATP synthase complex	GO:0045259	$8.26x10^{-03}$
GO:CC	ribosomal subunit	GO:0044391	$8.56x10^{-03}$
GO:CC	proton-transporting two-sector ATPase complex. catalytic domain	GO:0033178	0.01
GO:CC	respiratory chain complex	GO:0098803	0.01
GO:CC	intracellular organelle	GO:0043229	0.02
GO:CC	cytosol	GO:0005829	0.02
GO:CC	large ribosomal subunit	GO:0015934	0.02
GO:CC	organelle	GO:0043226	0.02
GO:CC	oxoglutarate dehydrogenase complex	GO:0045252	0.02
GO:CC	intracellular anatomical structure	GO:0005622	0.02
GO:CC	proton-transporting two-sector ATPase complex	GO:0016469	0.03
GO:CC	nucleosome	GO:0000786	0.03
GO:CC	mitochondrial inner membrane	GO:0005743	0.03
GO:CC	tricarboxylic acid cycle heteromeric enzyme complex	GO:0045239	0.03
GO:CC	organelle inner membrane	GO:0019866	0.03
GO:CC	sarcoplasmic reticulum membrane	GO:0033017	0.03
GO:CC	protein-DNA complex	GO:0032993	0.04
GO:CC	intermediate filament cytoskeleton	GO:0045111	0.04
GO:CC	alpha-ketoacid dehydrogenase complex	GO:0045240	0.04
GO:CC	intermediate filament	GO:0005882	0.04
GO:CC	cation channel complex	GO:0034703	0.05
GO:CC	cytosolic small ribosomal subunit	GO:0022627	0.05
GO:CC	sarcoplasmic reticulum	GO:0016529	0.05
